# Many-body interactions resolve halide distribution at the air/water interface

**DOI:** 10.1038/s41467-026-77205-9

**Published:** 2026-08-29

**Authors:** H. Agnew, R. Rashmi, X. Zhu, S. Dasgupta, F. Paesani

**Affiliations:** 1 Department of Chemistry, University of California, San Diego, La Jolla, CA USA; 2 Materials Science and Engineering, University of California, San Diego, La Jolla, CA USA; 3 Halicioğlu Data Science Institute, University of California, San Diego, La Jolla, CA USA; 4 San Diego Supercomputer Center, University of California, San Diego, La Jolla, CA USA

**Keywords:** Computational chemistry, Thermodynamics, Atmospheric chemistry, Statistical mechanics, Molecular dynamics

## Abstract

Whether dissolved ions approach or avoid the air/water interface remains unsettled, despite its importance for atmospheric and interfacial chemistry. Here we show, using chemically accurate data-driven many-body models and a controlled hierarchy of interaction descriptions, that the intrinsic surface propensity of halide ions depends critically on short-range many-body interactions beyond classical polarization. A conventional polarizable model predicts strong iodide adsorption and weaker stabilization of bromide and chloride. When short-range two- and three-body quantum-mechanical interactions are included, adsorption is strongly suppressed: fluoride, chloride, and bromide remain bulk-favored, while iodide retains only a shallow free-energy minimum comparable to thermal energy. Energy decomposition reveals that iodide’s residual preference reflects localized reorganization of interfacial water that offsets the cost of partial dehydration, but is opposed by entropy. These results unify classical electrostatic exclusion and molecular many-body descriptions, showing that halide distributions are controlled not by ion size or polarizability alone, but by a finely balanced collective response of the ion–water system.

## Abstract (web summary)

How halide ions distribute at the surface of water has long been debated. Here, the authors show that cooperative molecular interactions suppress adsorption, leaving only iodide with a weak surface preference.

## Introduction

Ions at aqueous interfaces play pivotal roles in diverse chemical and biological processes^1^. For example, the distributions of ions near the air/water interface can influence atmospheric chemistry and climate^2^, while specific ion effects in aqueous environments are key to several biological processes ranging from membrane interactions to protein aggregation^3^. In this context, halide ions offer a stringent test of any theory of specific ion effects because their interactions with water span diverse length scales from local hydration to collective fluctuations of the surrounding hydrogen-bond network. Despite long-standing efforts, fundamental questions persist about how halides partition between bulk water and the air/water interface^4,5^.

Early experimental studies of electrolyte solutions at the air/water interface revealed pronounced ion-specific signatures. In particular, surface tension measurements showed a strong anion dependence consistent with an inverse Hofmeister trend, with iodide producing the smallest increase and fluoride the largest^6^, while electrostatic potential differences across the air/water interface were found to be more negative for halide solutions than for neat water (with fluoride solutions as an exception)^7^. These observations implied an enhanced interfacial presence of the heavier halides (Br^−^ and I^−^), directly challenging Debye–Hückel theory^8^ and classical image-charge arguments advanced independently by Wagner^9^ and by Onsager and Samaras^10^, which predict generic ion exclusion.

Advanced experimental probes, including vibrational sum-frequency generation (vSFG) and photoelectron (PE) spectroscopy, have offered rich but sometimes conflicting interpretations: vSFG measurements range from substantial spectral changes consistent with halide accumulation to negligible changes in the free OH peak upon isotopic dilution^11,12^, while PE studies indicate enhanced near-surface concentrations of Br^−^ and I^−^^13^. More recent vSFG studies have reached opposing conclusions, ranging from reports of extreme surface propensity^14^ to models of a stratified interface in which ions occupy subsurface layers beneath an ion-depleted topmost layer^15^.

Molecular dynamics (MD) simulations have yielded model-dependent predictions for halides at the air/water interface. For simple alkali-halide salts, conventional fixed-charge, non-polarizable force fields generally predict little or no halide surface affinity, whereas empirical polarizable models often stabilize the larger, softer anions in the interfacial region^2,16–22^.

Ab initio molecular dynamics (AIMD) simulations based on density functional theory (DFT) have added nuance but also variability^23^, as predicted surface propensities can depend sensitively on the chosen exchange–correlation functional, with well-documented functional- and density-driven errors complicating quantitative interpretation, particularly for anions^24–26^. Combining MD simulations with polarizable force fields with electronic-structure calculations of vertical ionization energies has suggested that changes in extended hydrogen-bond networks can contribute to interfacial stabilization^27^.

Several theoretical models have also been proposed, including dielectric-continuum descriptions of polarizable anions^28^ and subsequent extensions that combine ionic polarizability with cavitation and interfacial solvation effects^29^, as well as fluctuation-based descriptions that emphasize interfacial density and electric-field fluctuations^30,31^, offering complementary perspectives on how molecular-scale structure informs macroscopic observables.

A quantitative molecular description of halide distributions at the air/water interface requires a water model that is accurate not only in the bulk but also in the highly heterogeneous interfacial environment. In this regard, MB-pol^32–35^ is distinguished by its demonstrated ability to reproduce structural, thermodynamic, dynamical, and spectroscopic properties of water from small clusters to liquid water and ice^36–38^, including vapor–liquid coexistence and interfacial properties^39^. Earlier MB-pol studies also reproduced the vSFG spectrum of the air/water interface and its temperature and polarization dependence^40–43^, and recent work further showed that MB-pol accurately captures polarization-resolved signatures of free OH groups and links them to orientation-dependent local electric fields, providing a realistic description of interfacial heterogeneity^44^.

Recently, data-driven many-body energy (MB-nrg) potentials have extended the MB-pol framework to ions in water by rigorously representing the many-body expansion (MBE) of the energy through machine-learned short-range ion–water *n*-body terms trained on “gold-standard” coupled-cluster electronic-structure data, combined with physics-based representations of ion–water many-body polarization^45,46^. Within MB-nrg, the short-range machine-learned terms encode quantum-mechanical effects that are absent from classical force fields, including exchange-repulsion, charge penetration, and charge transfer^47,48^. Across the halide series, MB-nrg potentials have accurately reproduced interaction energies, tunneling pathways, and vibrational spectra from the dimer to larger gas-phase clusters^49–52^, as well as hydration structure, vibrational and X-ray spectra, and free energies in solution^53–56^.

Leveraging the accuracy and predictive power of MB-nrg potentials, we pinpoint the mechanisms that govern specific halide effects at the air/water interface. By systematically comparing a hierarchy of molecular models, we separate the full ion–water contribution, which encompasses pairwise interactions, permanent electrostatics, many-body polarization, and short-range quantum many-body effects, from the corresponding water cavity/reorganization contribution. This analysis reveals that only I^−^ retains a slight interfacial preference, with the associated energetic signature localized in a narrow minimum of the water cavity/reorganization contribution that compensates the ion–water desolvation penalty near the interface.

## Results

### A many-body hierarchy of halide surface propensity

To resolve the physical origin of halide surface propensities, we examine a hierarchy of ion–water models built on top of MB-pol^32–38^. The Thole-type-model energy (TTM-nrg) potentials^57^ couple MB-pol with an ion–water polarizable model that includes permanent electrostatics and classical many-body polarization via an extended Thole scheme^58,59^, together with pairwise terms representing repulsion and dispersion interactions, similar to conventional polarizable models used in previous studies^2,16–21^.

The MB-nrg potentials^45^ refine ion–water interactions using the established 2B/3B/NB notation^53,60^: “2B” denotes explicit 2-body ion–water terms, “3B” denotes the explicit short-range 3-body ion–water–water term, and “NB” denotes the classical N-body polarization contribution represented through the extended Thole scheme. Accordingly, (i) (2B)-MB-nrg includes a machine-learned short-range 2-body ion–water term, together with permanent electrostatics and pairwise dispersion of the same functional forms used in TTM-nrg; (ii) (2B+NB)-MB-nrg augments (2B)-MB-nrg with the NB many-body polarization term; and (iii) (2B+3B+NB)-MB-nrg further adds the explicit machine-learned 3-body ion–water–water term that captures short-range nonadditive quantum effects.

All potentials were trained on electronic-structure data computed at the coupled-cluster level of theory, including single, double, and perturbative triple excitations, i.e., CCSD(T)^61^, which is widely regarded as the “gold standard” for chemical accuracy^62^. Specific details about the TTM-nrg and MB-nrg potentials are provided in the Supporting Information.

**Fig. 1:**
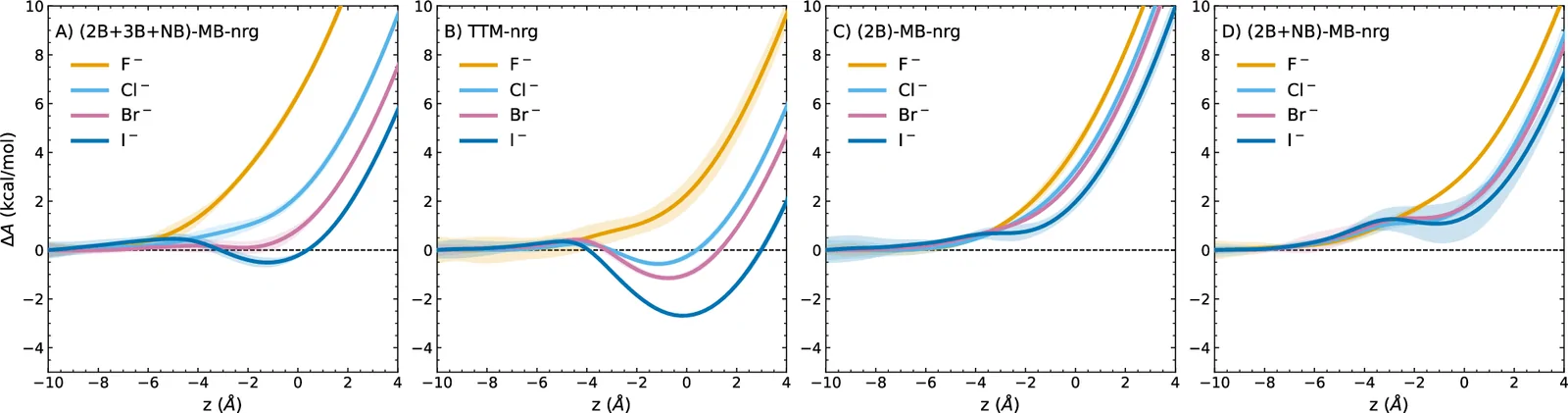
Many-body effects suppress halide adsorption. Helmholtz free-energy profiles (Δ*A*) calculated with the different ion--water potentials along the surface normal referenced to the GDS at *z* = 0. **A** (2B+3B+NB)-MB-nrg; **B** TTM-nrg; **C** (2B)-MB-nrg; **D** (2B+NB)-MB-nrg. The reported PMF is the mean of the three 4-ns block-specific estimates, and the shaded bands represent two-sided 95% Student’s *t* confidence intervals calculated from these estimates. Fully many-body (2B+3B+NB)-MB-nrg predicts that only I^−^ (blue) retains an interfacial minimum of order  ~ k_B_*T*, whereas Br^−^ (reddish purple), Cl^−^ (sky blue), and F^−^ (orange) are not stabilized at the interface. TTM-nrg recovers the strong adsorption of I^−^ and non-negligible minima for Br^−^ and Cl^−^ typical of empirical polarizable force fields. Pairwise additive (2B)-MB-nrg yields repulsive Δ*A* for all ions, in agreement with classical theories, and adding classical many-body polarization generates only shallow local minima near the GDS that remain marginal compared to bulk hydration. Analogous analyses based on the corresponding Willard–Chandler instantaneous interface^82^ are reported in Supplementary Fig. S4 of the Supporting Information. Source data are provided as a tarball.

Figure 1 reports the Helmholtz free-energy profiles, Δ*A*(*z*), calculated along the surface-normal coordinate for the four halides across the model hierarchy discussed above, with the Gibbs dividing surface (GDS) at *z* = 0. Figure 1A shows the central result: with the fully many-body (2B+3B+NB)-MB-nrg potentials, only I^−^ exhibits a shallow interfacial minimum centered at the GDS, whereas F^−^, Cl^−^, and Br^−^ remain at or above the bulk reference throughout the interfacial region and are not stabilized at the surface. Thus, in contrast to the pronounced adsorption predicted by previous studies with polarizable force fields^2,16–21^, accurately accounting for many-body interactions across the hierarchy of the MBE strongly suppresses interfacial adsorption across the halide series, leaving at most a slight preference of order k_B_*T* for I^−^, where k_B_ is Boltzmann’s constant.

To corroborate that the strong interfacial propensity reported with polarizable models stems from their conventional parameterization of ion–water interactions, Figure 1B shows that the polarizable TTM-nrg potential indeed yields a pronounced interfacial free-energy minimum for I^−^, smaller but distinct minima for Br^−^ and Cl^−^, and repulsion for F^−^. This is the same qualitative trend reported by empirical polarizable force fields^2,16–21^. Figures 1A, B therefore show that the strong adsorption predicted by polarizable models vanishes once *n*-body ion–water interactions are included with chemical accuracy, as in the (2B+3B+NB)-MB-nrg potentials. This comparison identifies ion–water many-body effects as the decisive factor controlling halide distributions at the air/water interface.

Figures 1C, D isolate how pairwise additivity, classical many-body polarization, and explicit short-range many-body effects shape halide behavior at the air/water interface within the MB-nrg hierarchy. When ion–water interactions are described by the purely pairwise-additive (2B)-MB-nrg potentials, all free-energy profiles are repulsive across the interface (*ΔA*≥0). Introducing many-body polarization in the (2B+NB)-MB-nrg potentials generates only shallow local minima near the GDS for Cl^−^, Br^−^, and I^−^, while F^−^ remains repelled although the bulk state remains thermodynamically preferred in all cases. When explicit short-range 3-body ion–water interactions are further included, as in the (2B+3B+NB)-MB-nrg potentials, the minima for Cl^−^ and Br^−^ disappear and the I^−^ well compresses to a near-thermal depth. These results indicate that the weak interfacial stabilization observed only for I^−^ emerges from a subtle interplay between the collective response of interfacial water cavity/reorganization effects and short-range, nonadditive ion–water interactions.

The model hierarchy also clarifies connections to classical and continuum theories. The purely pairwise-additive (2B)-MB-nrg description reproduces the generic exclusion predicted by Wagner and by Onsager–Samaras at a dielectric discontinuity. By contrast, combining many-body polarization with conventional pairwise-additive repulsion and dispersion, as in TTM-nrg, yields a pronounced interfacial minimum for I^−^ and smaller minima for Br^−^ and Cl^−^, qualitatively consistent with the original dielectric-continuum treatment of polarizable anions, in which charge redistribution within a polarizable ion lowers the electrostatic penalty for approaching the dielectric interface^28^. When many-body polarization is integrated with an explicit 2-body ion–water term, as in (2B+NB)-MB-nrg, the resulting profiles are everywhere non-negative, with only shallow near-surface local minima, in qualitative agreement with the extended continuum theory in which ionic polarizability is combined with cavitation and interfacial solvation effects^29^. In this sense, the full (2B+3B+NB)-MB-nrg potential provides a microscopic, many-body realization of the cavity/reorganization physics that enters continuum descriptions phenomenologically: the explicit short-range 2-body and 3-body ion–water terms do not constitute a cavitation term per se, but instead determine how the surrounding water environment reorganizes and thereby modulate the effective cavity/reorganization contribution. The fact that (2B+NB)-MB-nrg already resembles the extended continuum picture indicates that accurate short-range 2-body ion–water physics captures much of the same net effect represented phenomenologically as cavitation, while the further changes induced by explicit 3-body interactions show that this cavity/reorganization physics is itself modulated by higher-order many-body effects absent from continuum models. Importantly, the disappearance of interfacial minima for Cl^−^ and Br^−^ upon inclusion of explicit 3-body interactions highlights a key limitation of continuum approaches, namely the absence of quantum-mechanical many-body effects that ultimately modulate halide surface propensity.

In this context, it is worth noting that the near-thermal interfacial adsorption predicted by (2B+3B+NB)-MB-nrg for I^−^ is qualitatively consistent with AIMD simulations performed with the revPBE-D3 functional^23^. That agreement should, however, be interpreted cautiously. DFT descriptions of aqueous systems are strongly dependent on the chosen functional^63,64^ and can suffer from both functional-driven and density-driven errors^24,65^. In particular, analyses of water^25,66,67^ and halide–water^26,45,47,68^ interactions have shown that common density functionals often fail to capture short-range quantum-mechanical effects associated with large intermolecular orbital overlap, including self-interaction, charge penetration, charge transfer, and delocalization errors. These findings have recently prompted a reassessment of the predictive power of widely used DFT models for aqueous systems^69,70^.

To provide a complementary spectroscopic signature of the many-body hierarchy observed in the Helmholtz free-energy profiles, we calculated the imaginary component of the resonant heterodyne-detected vSFG (HD-vSFG) response, ${{{\rm{Im}}}}\,{\chi }_{XXZ}^{(2)}(\omega )$, for the same single-halide air/water interfaces and ion–water potentials used in Fig. 1. The calculated HD-vSFG spectra, shown in Supplementary Fig. 17, are not intended for direct quantitative comparison with finite-concentration vSFG measurements of electrolyte solutions^11,12,14,15^ because the present simulations probe the dilute single-ion limit and therefore exclude counterions, ion–ion correlations, ion pairing, and concentration-dependent interfacial restructuring. Nevertheless, the full (2B+3B+NB)-MB-nrg spectra do not support the extreme-propensity interpretation proposed in ref. ^14^, according to which the heavier halides exhibit strong surface propensity. Instead, they are qualitatively consistent with the more recent HD-vSFG measurements of electrolyte solutions reported in ref. ^15^, in the sense that they do not show spectral signatures of strong halide accumulation in the topmost interfacial layer, but only an iodide-specific response. The interpretation, however, is different: in the present single-ion limit, the spectra reflect the intrinsic thermodynamic propensity of each halide for the air/water interface, whereas finite-concentration experiments may include additional effects from counterions, ion–ion correlations, and concentration-dependent stratification.

The calculated spectra closely follow the free-energy hierarchy. Consistent with Figure 1, TTM-nrg produces a positive hydrogen-bonded O–H feature near 3400 cm^−1^, accompanied by a decrease in the free O–H peak near 3700 cm^−1^, for I^−^, Br^−^, and Cl^−^, reflecting the enhanced surface propensity predicted by the polarizable baseline. In contrast, the full (2B+3B+NB)-MB-nrg potential yields an appreciable surface-specific hydrogen-bonded O–H response only for I^−^. The (2B)-MB-nrg spectra show no positive hydrogen-bonded O–H feature for any halide ion, whereas the (2B+NB)-MB-nrg spectra show only a weak positive feature near 3400 cm^−1^ for I^−^ within statistical uncertainty. Thus, the interfacial hydrogen-bonded O–H stretch response mirrors the same many-body hierarchy obtained from the PMFs, providing an independent spectroscopic consistency check of the predicted halide surface propensities.

### Desolvation versus water cavity/reorganization

To rationalize the full many-body results in Fig. 1A, we decompose the (2B+3B+NB)-MB-nrg Helmholtz free energy as

1 $$\Delta A=\Delta U-T\Delta S=\Delta {U}_{{{{\rm{ion-water}}}}}+\Delta {U}_{{{{\rm{water-water}}}}}-T\Delta S,$$

where *ΔU*_ion-water_ and *ΔU*_water-water_ denote the corresponding contributions to the total configurational energy within the MB-nrg Hamiltonian. Eq. (1) is therefore used here as a mechanistic decomposition of the total energy along the adsorption coordinate, rather than as a unique thermodynamic partition of the adsorption free energy into independent ion–water and water–water free-energy terms. Within the MB-nrg framework, *ΔU*_water-water_ is obtained by removing the ion from the sampled interfacial water configurations and evaluating the corresponding water energy. It therefore represents a water cavity/reorganization contribution associated with reorganizing the interfacial water environment and accommodating the cavity occupied by the ion at a given position. Conversely, ion-induced polarization and the explicit short-range ion–water 2-body and ion–water–water 3-body terms in the (2B+3B+NB)-MB-nrg potentials are included in *ΔU*_ion-water_. This decomposition is thus distinct from the direct/indirect thermodynamic partition introduced in refs. ^71^ and^ 72^.

**Fig. 2:**
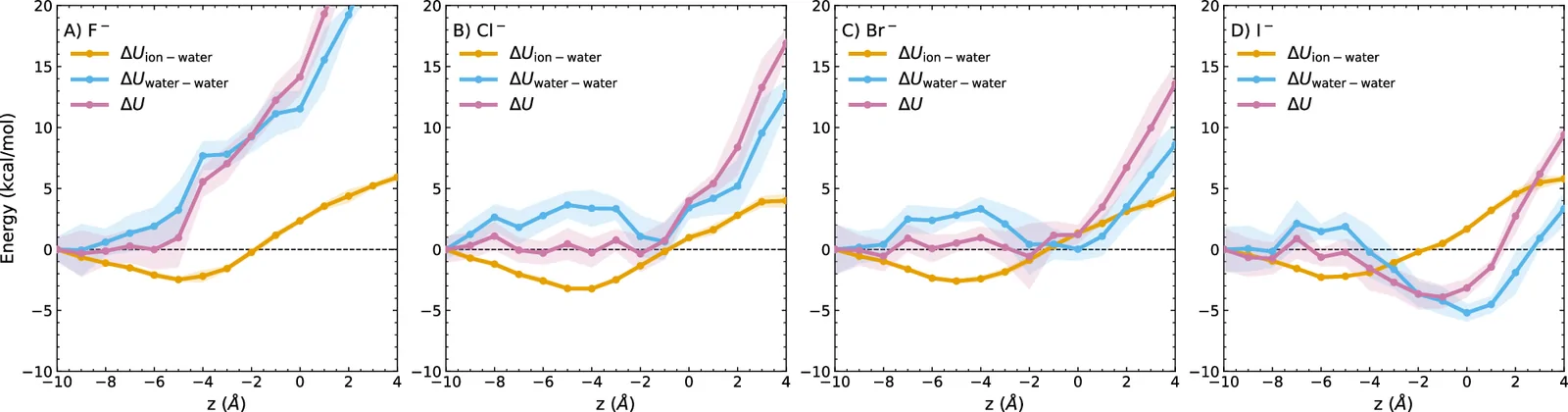
Competition between desolvation and water reorganization. Configurational energy decompositions calculated with the (2B+3B+NB)-MB-nrg potentials along the surface-normal coordinate referenced to the GDS at *z* = 0. Shown are the ion--water contribution *ΔU*_ion-water_ (orange), the water--water contribution *ΔU*_water-water_ (sky blue), and their sum *ΔU* (reddish purple) for (**A**) F^−^, (**B**) Cl^−^, (**C**) Br^−^, and (**D**) I^−^. Negative values denote stabilization relative to the bulk reference. The reported PMF is the mean of the three 4-ns block-specific estimates, and the shaded bands represent two-sided 95% Student’s *t* confidence intervals calculated from these estimates. All ions display a subsurface minimum in *ΔU*_ion-water_ (orange). The water cavity/reorganization contribution (*ΔU*_water-water_) differentiates the series: it rises monotonically and becomes strongly positive near the interface for F^−^; it is non-monotonic with a local minimum (higher than the bulk reference value) near the GDS for Cl^−^ and Br^−^; it exhibits a clear minimum at the GDS only for I^−^, which drives a distinct minimum in the corresponding *ΔU* profile. The Supporting Information reports analogous analyses referenced to the instantaneous Willard-Chandler interface (Supplementary Fig. S5) and, for comparison at the GDS, results obtained with TTM-nrg (Supplementary Fig. S8), (2B)-MB-nrg (Supplementary Fig. S11), and (2B+NB)-MB-nrg (Supplementary Fig. S14). Source data are provided as a tarball.

Figure 2 displays *ΔU*_ion-water_, *ΔU*_water-water_, and their sum *ΔU* for each halide. Across the series, *ΔU*_ion-water_ exhibits a common motif: a broad subsurface minimum located at *z* ∈ [ − 6, − 2] Å, with depths clustered around  − 3 ± 1kcal/mol. The curvature of the minimum decreases slightly along the series, i.e., the well becomes broader and softer for the heavier anions. Approaching the interface, *ΔU*_ion-water_ rises as coordinating water molecules are lost and becomes positive near the GDS, reflecting a desolvation penalty that is similar across the halide series.

In sharp contrast, the water cavity/reorganization contribution *ΔU*_water-water_ differentiates the series. For F^−^, it increases nearly monotonically from the bulk and becomes strongly positive in the interfacial region (exceeding  + 15 kcal/mol), consistent with the energetic cost of forcing a rigid hydration shell into the hydrogen-bond-deficient topmost layer. For Cl^−^ and Br^−^, *ΔU*_water-water_ is non-monotonic: a subsurface maximum is followed by a shallow minimum near the GDS before rising steeply as the ion protrudes into air. Remarkably, only I^−^ exhibits a distinct minimum at the GDS, preceded by a subsurface maximum, signaling a transient lowering of the water cavity/reorganization contribution unique to iodide. Consistent with this ion-specific stabilization of the water cavity/reorganization contribution under reduced coordination, quantum dynamical simulations of halide–dihydrate complexes X^−^(H_2_O)_2_ show that as the halide is varied from Cl^−^ to I^−^, the halide–water hydrogen bond weakens while the intra-dimer water–water hydrogen bond strengthens, providing a minimal undercoordinated motif that mirrors the interfacial environment^50,51^.

The total configurational energy, *ΔU*, reflects the balance between ion–water desolvation and water cavity/reorganization contributions. For F^−^, Cl^−^, and Br^−^, cancellation between *ΔU*_ion-water_ and *ΔU*_water-water_ in the bulk and subsurface regions keeps *ΔU* near or above zero across the water slab, precluding energetic stabilization at the interface. By contrast, for I^−^ the desolvation penalty in *ΔU*_ion-water_ near the interface is offset by the concomitant minimum in *ΔU*_water-water_, producing a clear minimum in *ΔU* centered at the GDS with a depth of approximately  − 4kcal/mol. Within the present many-body decomposition, this minimum should therefore be viewed as the energetic signature of iodide-associated reorganization of the surrounding water environment, rather than as an independent solvent-only driving force.

### Microscopic origins of iodide selectivity

**Fig. 3:**
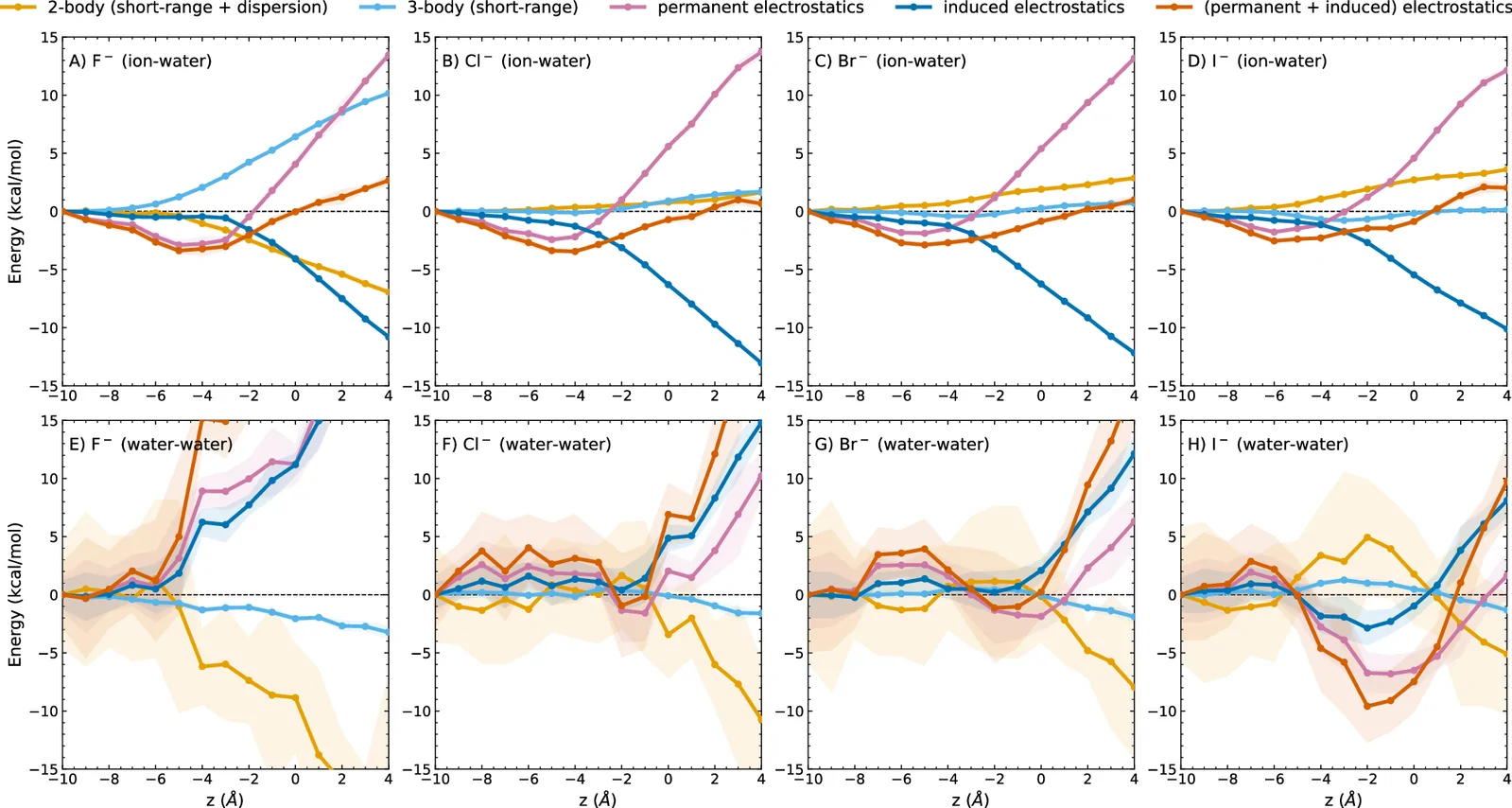
Many-body origin of iodide selectivity. Configurational energy contributions calculated with the (2B+3B+NB)-MB-nrg potentials along the surface normal referenced to the GDS at *z* = 0. Top row (**A**–**D**): ion–water contributions; bottom row (E–H): water--water contributions. Negative values denote stabilization relative to the bulk reference. The reported PMF is the mean of the three 4-ns block-specific estimates, and the shaded bands represent two-sided 95% Student’s *t* confidence intervals calculated from these estimates. Curves resolve 2-body (exchange-repulsion + charge penetration/transfer + dispersion) [orange], short-range 3-body (sky blue), and permanent (reddish purple) and induced electrostatics (blue), as well as total (permanent + induced) electrostatics [blue], as indicated in the legends. The Supporting Information reports analogous analyses referenced to the instantaneous Willard-Chandler interface (Supplementary Fig. S6) and, for comparison at the GDS, results obtained with TTM-nrg (Supplementary Fig. S9), (2B)-MB-nrg (Supplementary Fig. S12), and (2B+NB)-MB-nrg (Supplementary Fig. S15). Source data are provided as a tarball.

Figure 3 further resolves the configurational energy, *ΔU*, into ion–water (top row) and water cavity/reorganization (bottom row) contributions, thereby rationalizing the shapes of *ΔU*_ion-water_, *ΔU*_water-water_, and their sum in Fig. 2. For all halides, electrostatics dominates the subsurface well in *ΔU*_ion-water_: the permanent term becomes strongly positive as the ion approaches and crosses the GDS (desolvation), whereas induced electrostatics remains favorable throughout. Their sum yields a broad subsurface minimum that shifts modestly toward the GDS from F^−^ to I^−^. The 2-body term, which comprises the machine-learned short-range quantum-mechanical contribution together with dispersion, stabilizes F^−^ in the subsurface but becomes progressively destabilizing for Cl^−^, Br^−^, and I^−^ as first-shell water molecules are lost.

The explicit, machine-learned 3-body ion–water term softens systematically across the series: it is significantly positive for F^−^ throughout the subsurface and interfacial regions, becomes progressively less repulsive for Cl^−^ and Br^−^, and for I^−^ develops a shallow stabilizing minimum (≤1 kcal mol^−1^) in the subsurface. This trend indicates that short-range quantum-mechanical nonadditivity involving the ion and two water molecules strongly penalizes the rigid, tightly coordinated fluoride environment, but is progressively mitigated for the larger, softer halides and can even weakly stabilize iodide. The same trend parallels ion-mediated rearrangement propensities in halide–dihydrate complexes for which quantum dynamics simulations showed that the tunneling splitting for the halide–water hydrogen-bond bifurcation increases markedly from Cl^−^ to I^−^, while the splitting for the water–water bifurcation decreases along the same series^50,51^. Together with the strengthening of the water–water hydrogen bond from Cl^−^(H_2_O)_2_ to I^−^(H_2_O)_2_, these cluster-level specific-ion effects are consistent with the reduced short-range 3-body penalty and the selective stabilization of iodide near the interface. This analysis of the ion–water components thus directly rationalizes the subsurface minimum and subsequent rise in *ΔU*_ion-water_ shown in Fig. 2.

The water cavity/reorganization contribution discriminates the ions even more sharply. For F^−^, water–water electrostatics (both permanent and induced) grows strongly positive on approach to the interface and, although the water–water 2-body term becomes increasingly stabilizing, the net water cavity/reorganization contribution is highly destabilizing near the GDS. For Cl^−^, the water–water 2-body stabilization turns on closer to the GDS but is still offset by a positive electrostatic contribution near the interface. The heavier halides invert this balance: for Br^−^ and especially I^−^, water–water electrostatics becomes negative in a narrow window near the GDS (with I^−^ also exhibiting a negative induced component), while the water–water 2-body term is transiently positive in the same region, reflecting disruption of optimal pairwise hydrogen bonding by the larger, softer anions. The net result is a distinct minimum in *ΔU*_water-water_ for I^−^ at the GDS, preceded by a subsurface maximum (Fig. 2).

Adding *ΔU*_ion-water_ and *ΔU*_water-water_ makes the balance transparent: for F^−^, Cl^−^, and Br^−^, cancellations in the bulk and subsurface keep *ΔU* near or above zero across the interface, whereas for I^−^ the narrow water cavity/reorganization minimum near *z* ≈ 0 overcompensates the ion–water desolvation penalty, producing a clear minimum in *ΔU* centered at the GDS. Within the MB-nrg decomposition, this localized water cavity/reorganization minimum is the distinctive energetic signature associated with iodide’s weak interfacial preference.

These trends indicate that iodide is distinguished not by a more favorable ion–water interaction near the interface, but by an ion-specific minimum in the water cavity/reorganization contribution localized at the GDS. In the decomposition of Fig. 3, this minimum appears explicitly as a narrow lowering of the water–water electrostatic contribution that is absent for F^−^ and much weaker for Cl^−^ and Br^−^. Within the MB-nrg many-body decomposition, this feature should therefore be interpreted as the energetic signature of iodide-associated reorganization of the surrounding water environment, not as evidence that a solvent-only term independently drives adsorption.

### Energy-entropy balance of weak iodide adsorption

**Fig. 4:**
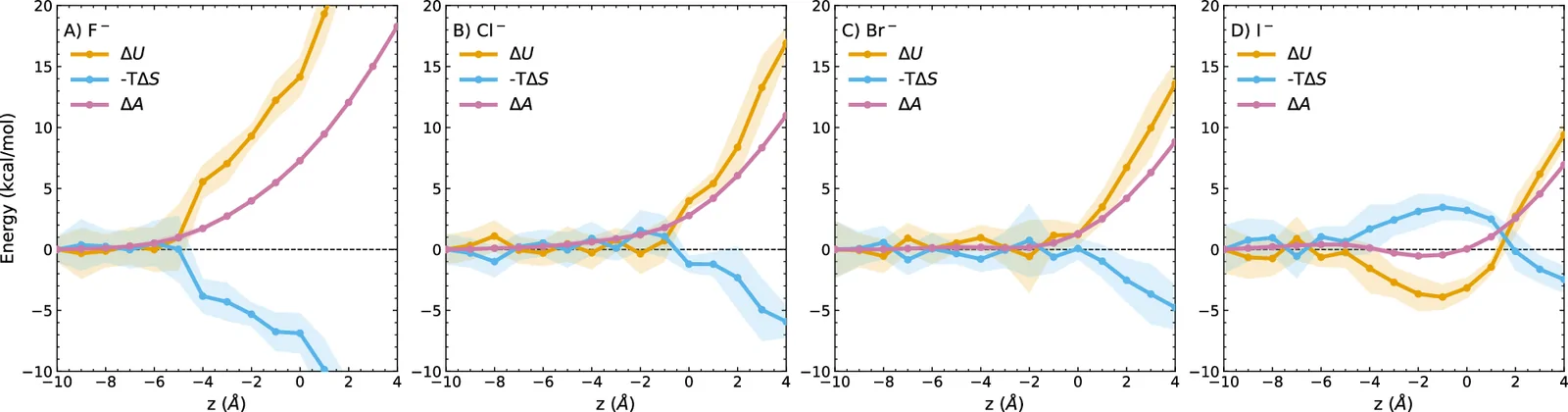
Energy-entropy balance limits iodide adsorption. Free-energy decompositions calculated with the (2B+3B+NB)-MB-nrg potentials along the surface-normal coordinate referenced to the GDS at *z* = 0. Helmholtz free energy *ΔA* (reddish purple) is decomposed into the configurational (potential) energy *ΔU* (orange) and the entropic contribution  − *TΔS* (sky blue) for (**A**) F^−^, (**B**) Cl^−^, (**C**) Br^−^, and (**D**) I^−^. Negative values indicate stabilization relative to the bulk reference. The reported PMF is the mean of the three 4-ns block-specific estimates, and the shaded bands represent two-sided 95% Student’s *t* confidence intervals calculated from these estimates. For F^−^, Cl^−^, and Br^−^, *ΔU* becomes positive near the interface and dominates over the entropic term, yielding *ΔA* ≳ 0 across the interfacial region. For I^−^, a distinct minimum in *ΔU* at the GDS is partially offset by an entropic penalty ( − *TΔS* > 0), compressing the net free-energy well to a shallow minimum of order k_B_*T* near *z* = 0. The Supporting Information reports analogous analyses referenced to the instantaneous Willard-Chandler interface (Supplementary Fig. S7) and, for comparison at the GDS, results obtained with TTM-nrg (Supplementary Fig. S10), (2B)-MB-nrg (Supplementary Fig. S13), and (2B+NB)-MB-nrg (Supplementary Fig. S16). Source data are provided as a tarball.

Finally, Fig. 4 resolves the Helmholtz free energy across the water slab into its energetic (*ΔU*) and entropic ( − *TΔS*) components (Eq. (1)). For F^−^, Cl^−^, and Br^−^, *ΔU* remains near or above the bulk reference throughout the interfacial region, while the entropic contribution provides only modest modulation near the GDS. Consequently, *ΔA* ≳ 0 across the interface for these ions.

Iodide exhibits a distinct thermodynamic balance. As established in Fig. 2, *ΔU* for I^−^ develops a clear minimum at the GDS, associated within the MB-nrg many-body decomposition with a favorable water cavity/reorganization contribution near the GDS, while  − *TΔS* is positive in the same region, indicating an entropic penalty for placing I^−^ at the surface. The partial cancellation between this energetic gain and the opposing entropic cost compresses the net free-energy well to a shallow minimum of order k_B_*T*.

This energy-driven yet entropy-opposed stabilization is qualitatively consistent with previous thermodynamic analyses of interfacial ion adsorption, which likewise found concurrent energetic stabilization and entropic penalty near the interface^30,31^. At the same time, the present results do not require assigning the entropy penalty specifically to capillary-wave suppression. Rather, within the MB-nrg decomposition, they indicate that iodide’s weak surface preference reflects a localized energetic stabilization associated with interfacial solvent reorganization, together with an opposing loss of interfacial configurational freedom. This interpretation is also consistent with thermodynamic descriptions that treat adsorption-induced changes in water–water interactions as part of the collective solvent response to the ion, rather than as an independent solvent-only driving force^71,72^. The modest entropic modulation for Cl^−^ and Br^−^, together with the localized energetic gain unique to I^−^, is also consistent with quantum simulations of halide–dihydrate complexes showing that undercoordinated topologies permit ion-specific rearrangements most readily for the softer halides^51^.

The present many-body analyses performed within the MB-nrg framework thus suggest a middle-ground picture of halides at the air/water interface.

Because the present calculations probe the dilute single-ion limit, they are not directly comparable to finite-concentration measurements and simulations, nor can the resulting PMFs be directly converted into surface-tension increments for finite-concentration electrolyte solutions. Through the Gibbs adsorption equation, electrolyte surface tensions depend on the surface excess of the electroneutral salt and therefore require both cation and anion distributions, ion–ion correlations, possible ion pairing, and concentration-dependent interfacial restructuring. The shallow iodide minimum found here should therefore be interpreted as an intrinsic single-ion propensity that can reduce the surface-tension increment of alkali-metal iodide solutions relative to corresponding salts of less surface-active halides, rather than as evidence for a negative surface-tension increment of alkali-metal iodide solutions. Consistent with this interpretation, NaI solutions experimentally increase the surface tension with concentration, but less strongly than NaCl solutions^73^. Within this single-ion scope, the present results do not indicate either the extreme surface propensity suggested by early polarizable-model simulations^2^ and inferred from some vSFG measurements^14^, or the ion-depleted topmost interfacial layer inferred in more recent stratification analyses^15^. Instead, they are most consistent with modest iodide surface activity, while Br^−^ and Cl^−^ remain thermodynamically bulk-favored. In this sense, the present findings remain broadly consistent with theoretical descriptions that incorporate ionic polarizability and cavitation^28,29^ and are qualitatively compatible with photoelectron evidence for near-surface enrichment of the heavier halides^13^.

## Discussion

We have shown that halide distributions at the air/water interface are controlled by a delicate balance between short-range ion–water interactions, which are quantum mechanical in origin, and water cavity/reorganization effects at the interface. Using data-driven many-body MB-nrg potentials that reproduce ion–water and water–water interactions with chemical accuracy across length scales, we analyzed a systematically controlled hierarchy of ion–water models. Within this hierarchy, purely pairwise-additive interactions exclude all halides, consistent with Wagner and Onsager–Samaras image-charge arguments, whereas classical polarization combined with conventional repulsion and dispersion over-stabilizes the heavier halides, as in empirical polarizable force fields. The full (2B+3B+NB)-MB-nrg potentials instead yield an intermediate regime in which interfacial stabilization is strongly suppressed for F^−^, Cl^−^, and Br^−^, and reduced to a near-thermal minimum for I^−^.

Within the MB-nrg decomposition, I^−^ is distinctive because a narrow minimum in the water cavity/reorganization contribution at the GDS offsets the ion–water desolvation penalty in the configurational energy. The resulting free-energy well nevertheless remains shallow because this energetic gain is opposed by the entropic cost of interfacial structuring. In this way, our results reconcile the qualitative insights of polarizable-anion continuum models with the more restrictive classical image-charge picture: halide surface propensity is not a simple consequence of ionic softness, but the outcome of a finely balanced many-body competition between ion–water interactions and collective water reorganization. The MB-nrg framework therefore provides a rigorous and mechanistically transparent benchmark for interpreting surface-sensitive measurements.

## Methods

All molecular dynamics (MD) simulations were performed using the Large-scale Atomic/Molecular Massively Parallel Simulator (LAMMPS)^74^ coupled with PLUMED^75^ and interfaced with the MBX C++ library for both the MB-nrg and TTM-nrg PEFs^76,77^. Specifically, we employed the latest version of the MB-nrg PEFs for halide–water interactions as implemented in MBX v1.3^77^. Following prior MB-pol simulations of the air/water interface^39–44^, all MD simulations were performed at 298 K in the canonical (*NVT*, i.e., constant number of particles, volume, and temperature) ensemble using a slab geometry consisting of 511 water molecules and a single halide ion in a simulation box of dimensions 20 Å × 20 Å × 100 Å. The velocity-Verlet algorithm^78^ was used to propagate the equations of motion with a time step of 2.0 fs. The temperature was controlled with a global Nosé–Hoover chain of three thermostats with a characteristic time of 1 ps^79^. The initial configurations were taken from previous MB-nrg simulations and further equilibrated using the corresponding TTM-nrg and MB-nrg potentials for at least 100 ps. All results presented in the manuscript were obtained by constraining the water geometry with SHAKE^80^ to the average MB-pol geometry obtained from quantum path-integral molecular dynamics (PIMD) simulations^34^, corresponding to an OH bond length of 0.988 Å and an HOH bond angle of 105.5^∘^. The potentials of mean force (PMFs) were calculated from MD simulations performed using the on-the-fly probability enhanced sampling (OPES) method^81^ with 8 walkers sharing a common time-dependent bias and a BARRIER of 20 kcal/mol. Each walker was simulated for 12 ns, giving a total trajectory time of 96 ns per simulation. For uncertainty estimation, the 12-ns production period was partitioned into three consecutive 4-ns time blocks. Within each block, data from all eight walkers were combined and reweighted to obtain one block-specific PMF. The reported PMF is the mean of the three block-specific estimates, and the shaded bands represent two-sided 95% Student’s *t* confidence intervals calculated from these estimates. The 4-ns block length was selected based on convergence of the block-specific PMFs and to reduce temporal correlation between successive block estimates. Supplementary Fig. 2 shows the distribution of the ion positions of these walkers throughout the simulation trajectories, indicating that these walkers reversibly traverse the region of interest. The instantaneous Willard-Chandler interface^82^ was calculated using the DISTANCE_FROM_CONTOUR implementation within PLUMED^83^. The PLUMED input used for the OPES_METAD is included as Supplementary Fig. 1. PLUMED was also used to reweight the collective variables (CVs) to obtain the PMFs as a function of either the static or instantaneous surface. A comparison between the PMFs calculated with rigid and flexible MB-pol water molecules is reported in Figs. S1 of the Supporting Information. The results presented in Figs. 1–4 were obtained with respect to the mean (static) Gibbs dividing surface (GDS), while analogous analyses based on the corresponding Willard–Chandler instantaneous interface^82^ are provided in Figs. S4–S7 of the Supporting Information. Analyses analogous to those shown in Figs. 2–4 for the (2B+3B+NB)-MB-nrg potential are provided in the Supporting Information for the TTM-nrg (Supplementary Figs. S8–S10), (2B)-MB-nrg (Supplementary Figs. S11–S13), and (2B+NB)-MB-nrg (Supplementary Figs. S14–S16) potentials. Specific details about all many-body potentials and MD simulations are provided in the Supporting Information.

### Reporting summary

Further information on research design is available in the Nature Portfolio Reporting Summary linked to this article.

## Supplementary information

Supplementary Information

Reporting Summary

Transparent Peer Review file

## Source data

Source Data

## Data Availability

Source data for the figures are provided with this paper as a tarball. MD trajectories for the (2B+3B+NB)-MB-nrg potentials are publicly available on *Zenodo*^84^https://doi.org/10.5281/zenodo.19446735. Input files for all other trajectories are also available in the same Zenodo repository. The raw SFG trajectories are not available due to their enormous data size of 4.6 Terabytes, but the input files are available on Zenodo to reproduce the trajectories. All other MD trajectories as well as any other data analyzed for this study are available from the authors upon request. Source data are provided with this paper.

## Code availability

All MD simulations were carried out with LAMMPS^74^ and i-PI^85^ patched with the MBX v1.3 software for data-driven many-body simulations plugin^74,75^. The specific version of MBX+LAMMPS used is available as a tarball through Zenodo^84^https://doi.org/10.5281/zenodo.19446735, though newer versions of these software may have improved performance. The MBX+LAMMPS integration is publicly documented at https://docs.lammps.org/pair_mbx.html, and the source code is available at http://github.com/paesanilab/MBX.
