# Supplementary material for: Many-body interactions resolve halide distribution at the air/water interface
Source: Nat Commun. 2026 Aug 29;17:10313. doi: 10.1038/s41467-026-77205-9 (PMC13620159; doi:10.1038/s41467-026-77205-9)
Supplement: Supplementary file 1 [file 41467_2026_77205_MOESM1_ESM.pdf]

Supplementary Information

Many-Body Interactions Govern Halide Distribution at the Air/Water Interface

H. Agnew et al.

Contents

Supplementary Note 1	Many-body potential energy functions	3
Supplementary Note 2	Molecular dynamics simulations and analysis	6
Supplementary Note 3	Gibbs dividing surface (GDS)	8
Supplementary Note 4	Instantaneous Willard–Chandler interface (WCI)	9
Supplementary Note 5	Gibbs dividing surface (GDS): TTM-nrg	13
Supplementary Note 6	Gibbs dividing surface (GDS): (2B)-MB-nrg	16
Supplementary Note 7	Gibbs dividing surface (GDS): (2B+NB)-MB-nrg	19
Supplementary Note 8	vSFG spectra at the air/water interface with halide ions	22

Supplementary Note 1: Many-body potential energy functions

The energy, E_N, of a system with N atomic or molecular monomers can be expressed through the many-body expansion (MBE)[1] as a summation of individual n-body energies, ϵ_{nB}, with $n \leq N$,[2]

$$E_N(1,...,N) = \sum_{i=1}^{N} \epsilon^{1\mathrm{B}}(i) + \sum_{i<j}^{N} \epsilon^{2\mathrm{B}}(i,j) + \sum_{i<j<k}^{N} \epsilon^{3\mathrm{B}}(i,j,k) + ... + \epsilon^{\mathrm{NB}}(1,\ldots,N) \quad (1)$$

where the 1-body ($\epsilon^{1\mathrm{B}}$) energy corresponds to the monomer distortion energy in the case of molecular monomers. The higher-body terms are defined recursively as

$$\epsilon^{n\mathrm{B}} = E_n(1,...,n) - \sum_{i=1}^{N} \epsilon^{1\mathrm{B}} - \sum_{i<j}^{N} \epsilon^{2\mathrm{B}}(i,j) - ... - \sum_{i<j<k...}^{N} \epsilon^{(n-1)\mathrm{B}}(i,j,k,...) \quad (2)$$

Eq. 1 serves as the foundation for the MB-pol[3–8] potential energy function (PEF) describing the water–water interactions, and also for the TTM-nrg[9,10] and MB-nrg[11,12] PEFs describing ion–water interactions. Both TTM-nrg and MB-nrg ion–water PEFs are built on top of the MB-pol water–water PEF and, their most general forms, approximate Eq. 1 for a single ion interacting with N water molecules as

$$E_N = V^{2\mathrm{B}} + V^{3\mathrm{B}} + \ldots + V^{n\mathrm{B}} + V_{\mathrm{elec}} \quad (3)$$

where $n \leq N$ is the n-body level after which the explicit ion–water terms are truncated and V_{elec} is an electrostatic term based on a modified version of the Thole model[13] introduced in Ref. 14 and further refined for the MB-pol PEF.[3,4] V_{elec} represents permanent electrostatics by a sum of Coulomb interactions between smeared partial charges located on the ion and each water molecule (V_{perm}) as well as induced electrostatics (up to dipoles) by an implicit many-body polarization term (V_{pol}):

$$V_{\mathrm{elec}} = V_{\mathrm{perm}} + V_{\mathrm{pol}} \quad (4)$$

In the following, we briefly summarize the theoretical and computational aspects of the TTM-nrg and MB-nrg PEFs used in this study. The interested reader is directed to the original publications about the TTM-nrg[9,10] and MB-nrg[11,12] as well as the MBX software[15,16] for a more thorough description and specific details.

TTM-nrg potentials. TTM-nrg truncates the $V^{n\mathrm{B}}$ ion–water terms in Eq. 3 at $V^{2\mathrm{B}}$, and represents the total ion–water interactions as

$$E_N^{\mathrm{TTM-nrg}} = V^{2\mathrm{B}} + V_{\mathrm{elec}} \quad (5)$$

where $V_{\mathrm{elec}} = V_{\mathrm{perm}} + V_{\mathrm{pol}}$ as discussed above and $V^{2\mathrm{B}}$ is expressed as[9,10]

$$V^{2\mathrm{B}} = V^{2\mathrm{B}}_{\mathrm{short}} + V^{2\mathrm{B}}_{\mathrm{disp}} \quad (6)$$

Here, $V_{\rm short}^{\rm 2B}$ and $V_{\rm disp}^{\rm 2B}$ are the total 2-body repulsion and dispersion energies, respectively, between the ion and the oxygen and hydrogen atoms of each water molecule. $V_{\rm short}^{\rm 2B}$ is calculated as a sum of pairwise-additive Born-Mayer potentials:

$$V_{\rm short}^{\rm 2B} = \sum_{i=1}^{N} \left[\sum_{k \in {\rm W}_i} A_{Xk} e^{-\delta_{6,Xk} R_{Xk}} \right] \tag{7}$$

where R_{Xk} is the distance between the ion (X) and the oxygen and hydrogen atoms (k) of the ith water molecule (W$_i$), A_{Xk} and δ_{Xk} are fitting parameters optimized to reproduce 2-body reference energies calculated at the coupled cluster level of theory, including single, double, and perturbative triple excitations, i.e., CCSD(T). $V_{\rm disp}^{\rm 2B}$ is similarly calculated as a sum of pairwise-additive contributions:

$$V_{\rm disp}^{\rm 2B} = \sum_{i=1}^{N} \left[\sum_{k \in {\rm W}_i} -f(b_{Xk} R_{Xk}) \frac{C_{6,Xk}}{R_{Xk}^{6}} \right] \tag{8}$$

where R_{Xk} are the same interatomic distances as in Eq. 7, $C_{6,Xk}$ are the corresponding dispersion coefficients calculated using the exchange-hole dipole model (XDM),[17–19] and $f(b_{Xk} R_{Xk})$ are the Tang-Toennies damping functions,[20]

$$f(\delta_{Xk}, R_{Xk}) = 1 - \exp(-\delta_{Xk} R_{Xk}) \sum_{n=0}^{6} \frac{(\delta_{Xk} R_{kl})^n}{n!} \tag{9}$$

Here, δ_{Xk} are the same fitting parameters of Eq. 7.

(2B)-MB-nrg potentials. The (2B)-MB-nrg potentials are strictly pairwise additive, truncating the $V^{n\rm B}$ ion–water terms in Eq. 3 at $V^{\rm 2B}$ and neglecting the polarization term in the representation of electrostatic interactions:

$$E_N^{\rm (2B)-MB-nrg} = V^{\rm 2B} + V_{\rm perm} \tag{10}$$

$V^{\rm 2B}$ includes a 2-body machine-learned term ($V_{\rm ML}^{\rm 2B}$) along with the same $V_{\rm disp}^{\rm 2B}$,

$$V^{\rm 2B} = \sum_{i=1}^{N} V_{\rm ML}^{\rm 2B}(X, {\rm W}_i) + V_{\rm disp}^{\rm 2B} \tag{11}$$

Here, $V_{\rm ML}^{\rm 2B}$ is expressed as a product of a switching function and a permutationally invariant polynomial (PIP),[21]

$$V_{\rm ML}^{\rm 2B} = s^{\rm 2B} V_{\rm PIP}^{\rm 2B} \tag{12}$$

The switching function, $s^{\rm 2B}$, ensures that the contribution from the associated $V_{\rm ML}^{\rm 2B}$ term goes to zero whenever any subset of 1-mers is separated from the corresponding 2-mer. The linear and nonlinear parameters of $V_{\rm PIP}^{\rm 2B}$ were optimized by using Eq. 10 to reproduce a large set of ion–water dimer energies calculated at the CCSD(T) level of theory.[11]

(2B+NB)-MB-nrg potentials. The (2B+NB)-MB-nrg potentials truncate the $V^{n\mathrm{B}}$ ion–water terms in Eq. 3 at $V^{2\mathrm{B}}$ but, differently from (2B)-MB-nrg include many-body effects through the same polarization term used by TTM-nrg:

$$E_N^{(2\mathrm{B+NB})-\mathrm{MB-nrg}} = V^{2\mathrm{B}} + V_{\mathrm{perm}} + V_{\mathrm{pol}} \tag{13}$$

$V^{2\mathrm{B}}$ includes a 2-body machine-learned term (Eq. 12) and the same $V_{\mathrm{disp}}^{2\mathrm{B}}$ as in (2B)-MB-nrg. The linear and nonlinear parameters of the (2B+NB)-MB-nrg $V_{\mathrm{PIP}}^{2\mathrm{B}}$ term were optimized by using Eq. 13 to reproduce a large set of ion–water dimer energies calculated at the CCSD(T) level of theory.[11]

(2B+3B+NB)-MB-nrg potentials. The (2B+3B+NB)-MB-nrg potentials adopt the same functional form and the (2B+NB)-MB-nrg potentials but add an explicit $V^{3\mathrm{B}}$ term:

$$E_N^{(2\mathrm{B+3B+NB})-\mathrm{MB-nrg}} = V^{2\mathrm{B}} + V^{3\mathrm{B}} + V_{\mathrm{perm}} + V_{\mathrm{pol}} \tag{14}$$

$V^{3\mathrm{B}}$ is fully represented by a 3-body machine-learned term:

$$V^{3\mathrm{B}} = \sum_{\substack{i=1 \\ j>i}}^{N} V_{\mathrm{ML}}^{3\mathrm{B}}\left(X, \mathrm{W}_i, \mathrm{W}_j\right) \tag{15}$$

where $V_{\mathrm{ML}}^{3\mathrm{B}}$ is expressed as a product of a switching function and a PIP,[21]

$$V_{\mathrm{ML}}^{3\mathrm{B}} = s^{3\mathrm{B}} V_{\mathrm{PIP}}^{3\mathrm{B}} \tag{16}$$

The switching function, $s^{3\mathrm{B}}$, ensures that the contribution from the associated $V_{\mathrm{ML}}^{3\mathrm{B}}$ term goes to zero whenever any subset of the ion and the two water molecules is separated from the ion–water–water trimer. The linear and nonlinear parameters of $V_{\mathrm{PIP}}^{3\mathrm{B}}$ were optimized by using Eq. 14 to reproduce a large set of ion–water–water trimer energies calculated at the CCSD(T) level of theory.[11]

Supplementary Note 2: Molecular dynamics simulations and analysis

All molecular dynamics (MD) simulations were performed using the Large-scale Atomic/ Molecular Massively Parallel Simulator (LAMMPS) [22] coupled with PLUMED[23] and interfaced with the MBX C++ library for both the MB-nrg and TTM-nrg PEFs.[15,16] Specifically, we employed the latest version of the MB-nrg PEFs for halide–water interactions, as implemented in MBX v1.3.[16] The MD simulations were performed in the canonical (*NVT*) ensemble at 298 K. The simulation box was of dimensions 20 Å $\times$ 20 Å $\times$ 100 Å and contained a single ion and 511 water molecules. The initial configurations were taken from previous MB-nrg simulations and further equilibrated using the corresponding TTM-nrg or MB-nrg potential for at least $\sim$100 ps.

The potentials of mean force (PMFs) were calculated from MD simulations performed using the on-the-fly probability enhanced sampling (OPES) method[24] with 8 walkers sharing a common time-dependent bias and a BARRIER of 20 kcal/mol. Each walker was simulated for 12 ns, giving a total trajectory time of 96 ns per simulation. The instantaneous Willard-Chandler interface[25] was calculated using the DISTANCE_FROM_CONTOUR implementation within PLUMED.[26] The PLUMED input used for the OPES_METAD is included as Supplementary Figure 1. PLUMED was also used to reweight the collective variables (CVs) to obtain the PMFs as a function of either the static or instantaneous surface.

```
UNITS LENGTH=A ENERGY=kcal/mol TIME=ps

# w is the center of mass of bulk water
COM ATOMS=2-1534:3 LABEL=w NOPBC

# calculate distance for GDS
cl: GROUP ATOMS=1
dd: DISTANCE ATOMS=w,cl COMPONENTS NOPBC

md: OPES_METAD ARG=dd.z PACE=1000 BARRIER=20 TEMP=298 WALKERS_MPI
lw: LOWER_WALLS ARG=dd.z AT=9.0 KAPPA=5.0 EXP=2

# calculate contour for Willard-Chandler interface
id1: GROUP ATOMS=2-1534:3
dc: DISTANCE_FROM_CONTOUR POSITIONS=id1 ATOM=1 BANDWIDTH=2.4,2.4,2.4 DIR=z
    CONTOUR=0.016

bias: COMBINE ARG=*.bias PERIODIC=NO
PRINT STRIDE=50 ARG=dc.*,md.*,dd.z,bias,lw.bias FILE=COLVAR

FLUSH STRIDE=1000
```

Supplementary Figure 1: PLUMED input used for the metadynamics simulations carried out using the OPES algorithm.

For uncertainty estimation, the 12-ns production period was partitioned into three consecutive 4-ns time blocks. Within each block, data from all eight walkers were combined and reweighted to obtain one block-specific PMF. The reported PMF is the mean of the three block-specific estimates, and the shaded bands represent two-sided 95% Student's t confidence intervals calculated from

these estimates. The 4-ns block length was selected based on convergence of the block-specific PMFs and to reduce temporal correlation between successive block estimates. Supplementary Figure 2 shows the distribution of the ion positions of these walkers throughout the simulation trajectories, indicating that these walkers reversibly traverse the region of interest.

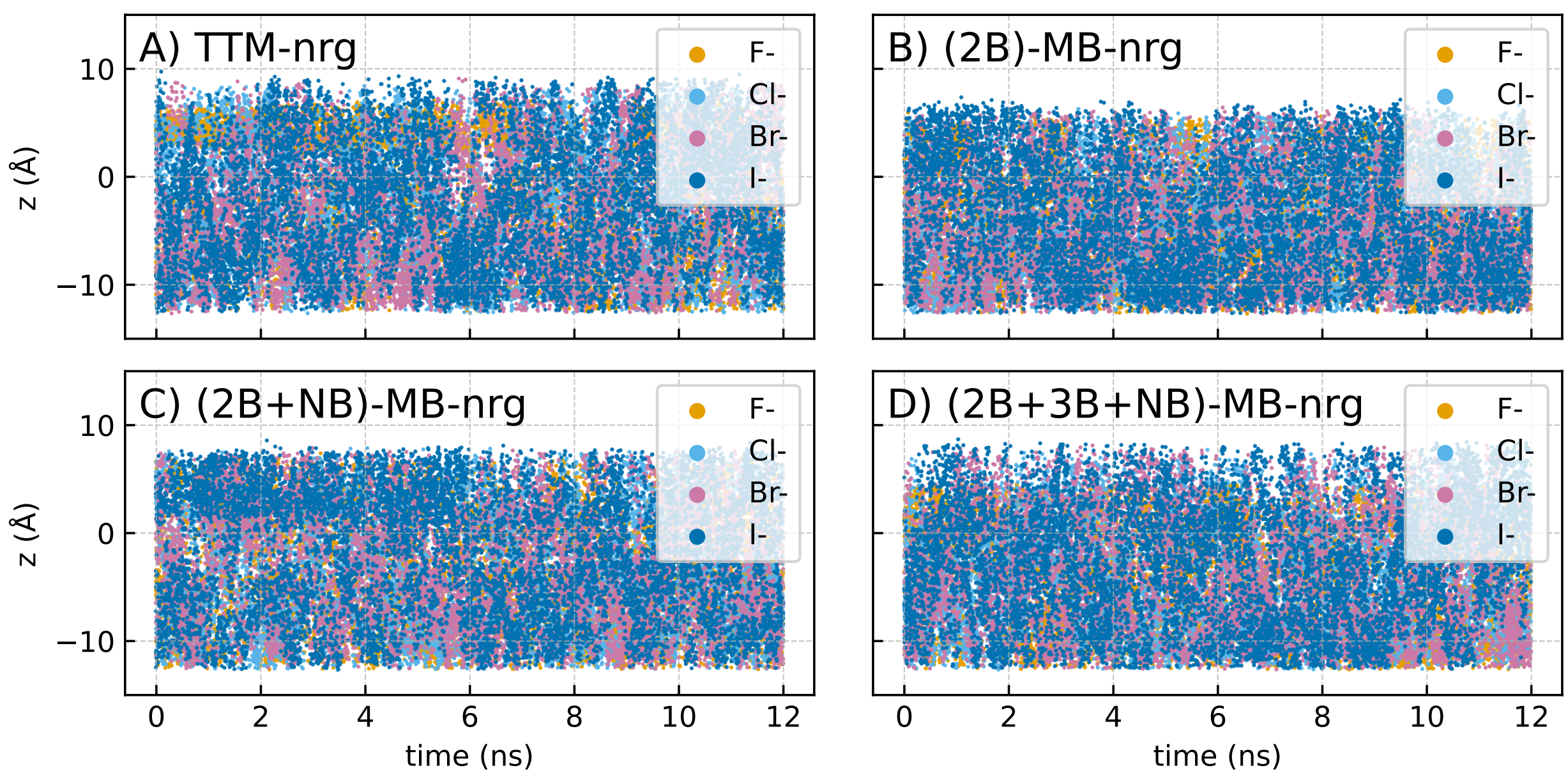

Supplementary Figure 2: Distribution of ion positions relative to the GDS throughout the simulation trajectories obtained with the TTM-nrg (A), (2B)-MB-nrg (B), (2B+NB)-MB-nrg (C), and (2B+3B+NB)-MB-nrg (D) potentials.

To speed up the MD simulations and improve the amount of sampling, the water geometry was constrained using SHAKE[27] to the average MB-pol geometry obtained from quantum path-integral molecular dynamics (PIMD) simulations, corresponding to an OH bond length of 0.988 Å and a HOH bond angle of 105.5°. The velocity-Verlet algorithm[28] was used to propagate the equations of motion with a timestep of 2.0 fs. The temperature was controlled by a global Nosé–Hoover chain of three thermostats with a characteristic time of 1 ps.[29]

Supplementary Note 3: Gibbs dividing surface (GDS)

This section validates the methodological choices adopted in the main text for the fully many-body (2B+3B+NB)-MB-nrg potential. In particular, Supplementary Figure S3 compares Helmholtz free-energy profiles obtained with OPES metadynamics, rigid-geometry umbrella sampling, and flexible-geometry umbrella sampling, showing that the resulting PMFs are very similar. These comparisons indicate that constraining the water geometry does not qualitatively alter the interfacial free-energy trends discussed in the main text.

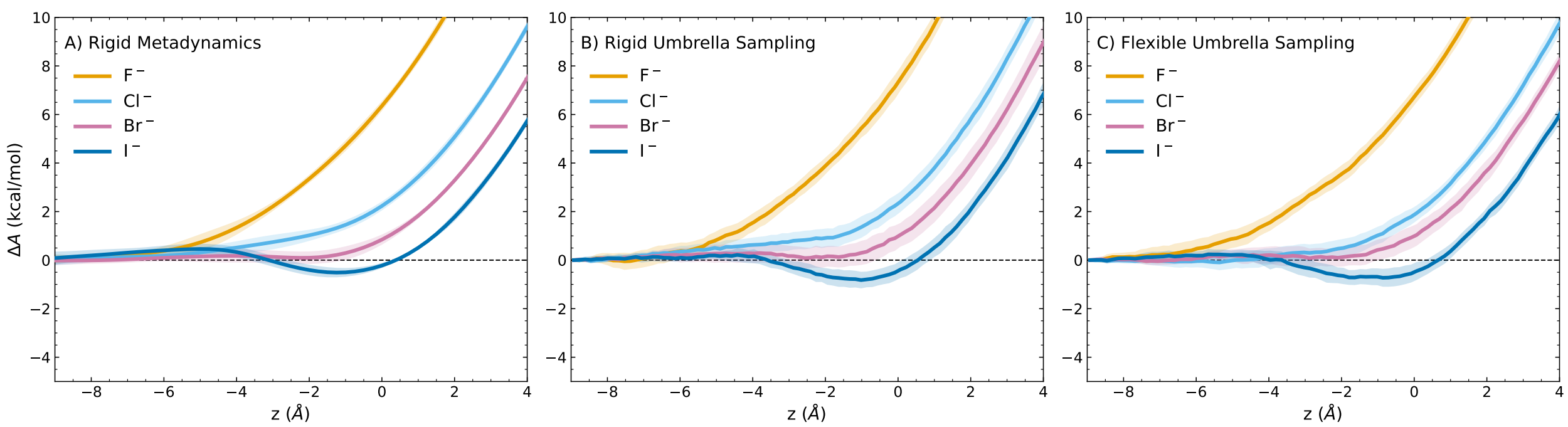

Supplementary Figure 3: Helmholtz free-energy profiles (ΔA) calculated of (2B+3B+NB)-MB-nrg along the surface-normal coordinate referenced to the GDS at $z = 0$ using (A) OPES Metadynamics, (B) rigid-geometry Umbrella Sampling[30], and (C) flexible-geometry Umbrella Sampling. For (A-B), the water geometry was constrained using the SHAKE algorithm. All ΔA profiles are similar, regardless of which enhanced-sampling method was used or whether the geometry of the water molecule was constrained or not.

Supplementary Note 4: Instantaneous Willard–Chandler interface (WCI)

This section reports analyses analogous to those presented in the main text, but referenced to the instantaneous Willard–Chandler interface (WCI)[25] rather than to the mean Gibbs dividing surface (GDS). Supplementary Figures S4–S7 show that the same qualitative conclusions are recovered for the fully many-body (2B+3B+NB)-MB-nrg potential and across the model hierarchy, while highlighting somewhat sharper near-surface variations associated with local interfacial fluctuations. These results demonstrate that the mechanistic picture described in the main text is robust with respect to the specific interfacial reference adopted.

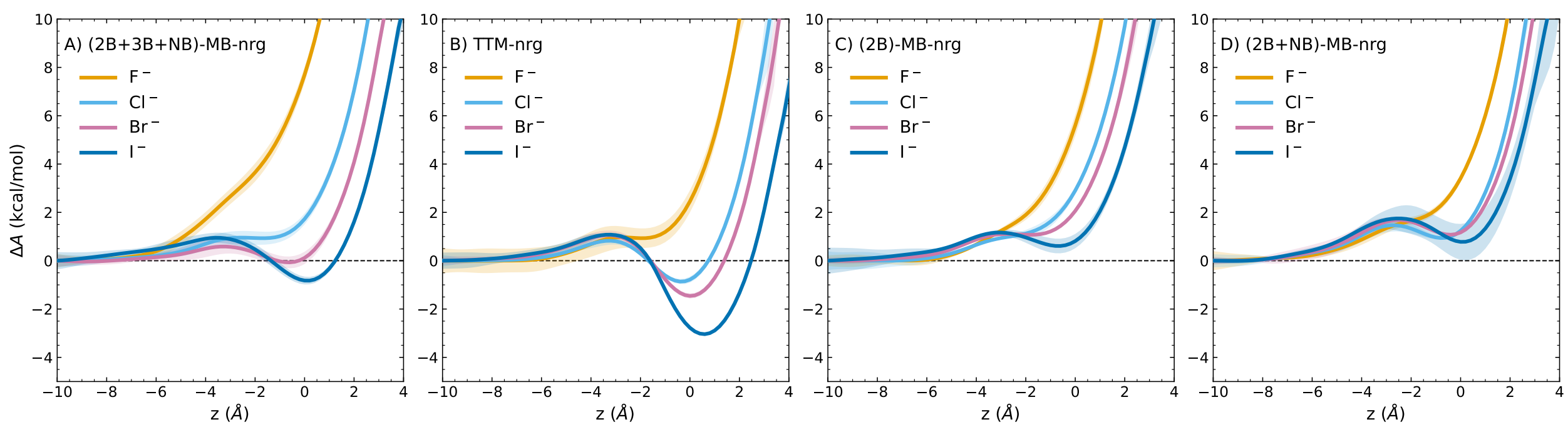

Supplementary Figure 4: Helmholtz free-energy profiles (ΔA) calculated with different ion–water models along the surface normal referenced to the WCI[25] at $z = 0$. (A) (2B+3B+NB)-MB-nrg; (B) TTM-nrg; (C) (2B)-MB-nrg; (D) (2B+NB)-MB-nrg. Negative values denote stabilization relative to the bulk reference. All ΔA profiles are similar to those shown relative to the Gibbs dividing surface (GDS) in Figure 1 of the main text. Specifically, fully many-body (2B+3B+NB)-MB-nrg predicts that only I^- retains an interfacial minimum of order $\sim \mathrm{k_B}T$, whereas Br^-, Cl^-, and F^- are not stabilized at the interface. TTM-nrg recovers strong adsorption of I^- and smaller minima for Br^- and Cl^- typical of empirical polarizable force fields. Pairwise additive (2B)-MB-nrg yields repulsive ΔA for all ions, in agreement with classical theories, and adding classical many-body polarization generates local minima near the WCI that remain marginal relative to bulk hydration. Compared to the GDS-referenced profiles in Figure 1 of the main text, the barrier upon approach to the interface is more pronounced when referenced to the instantaneous WCI, reflecting local interfacial fluctuations. Shaded bands denote the 95% confidence intervals.

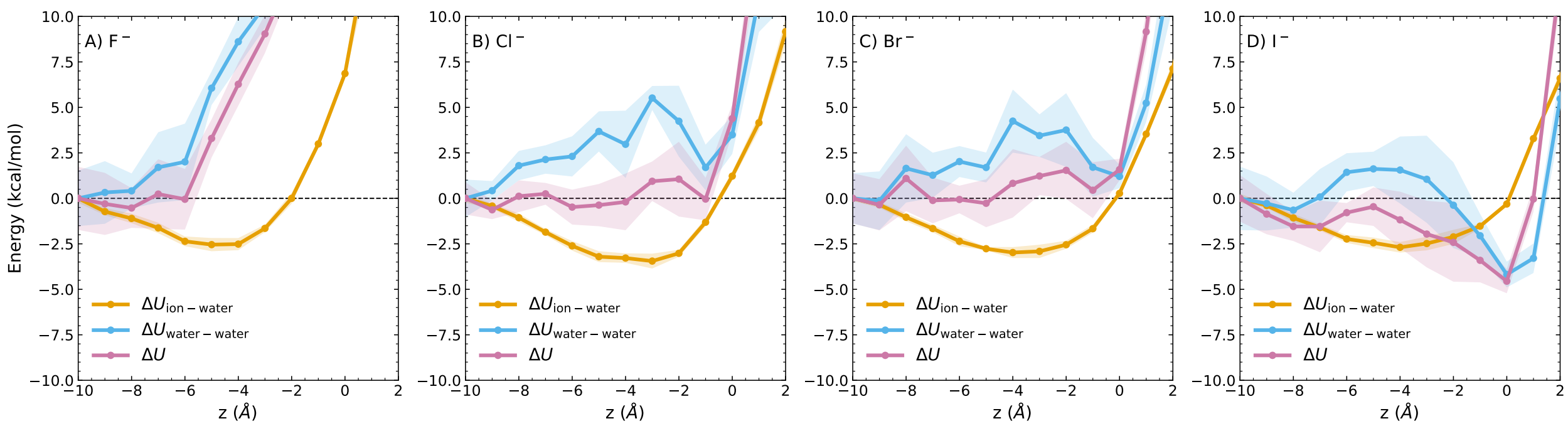

Supplementary Figure 5: Configurational energy decompositions calculated with the (2B+3B+NB)-MB-nrg potentials along the surface-normal coordinate referenced to the WCI[25] at $z = 0$. Shown are the ion–water contribution $\Delta U_{\mathrm{ion-water}}$ (orange), the water–water contribution $\Delta U_{\mathrm{water-water}}$ (sky blue), and their sum ΔU (reddish purple) for (A) F^-, (B) Cl^-, (C) Br^-, and (D) I^-. Negative values denote stabilization relative to the bulk reference. All energy profiles are similar to those shown relative to the Gibbs dividing surface (GDS) in Figure 2 of the main text. Specifically, all ions display a subsurface minimum in $\Delta U_{\mathrm{ion-water}}$ (orange). As in Figure 2, $\Delta U_{\mathrm{water-water}}$ differentiates the series: it rises monotonically and becomes strongly positive near the interface for F^-; it is non-monotonic with a local minimum (higher than the bulk reference value) near the WCI for Cl^- and Br^-; it exhibits a clear minimum at the WCI only for I^-, which drives a distinct minimum in the corresponding ΔU profile. Compared to the GDS-referenced profiles in Figure 2, the near-surface rise is sharper when referenced to the instantaneous WCI, reflecting local interfacial fluctuations. Shaded bands denote the 95% confidence intervals.

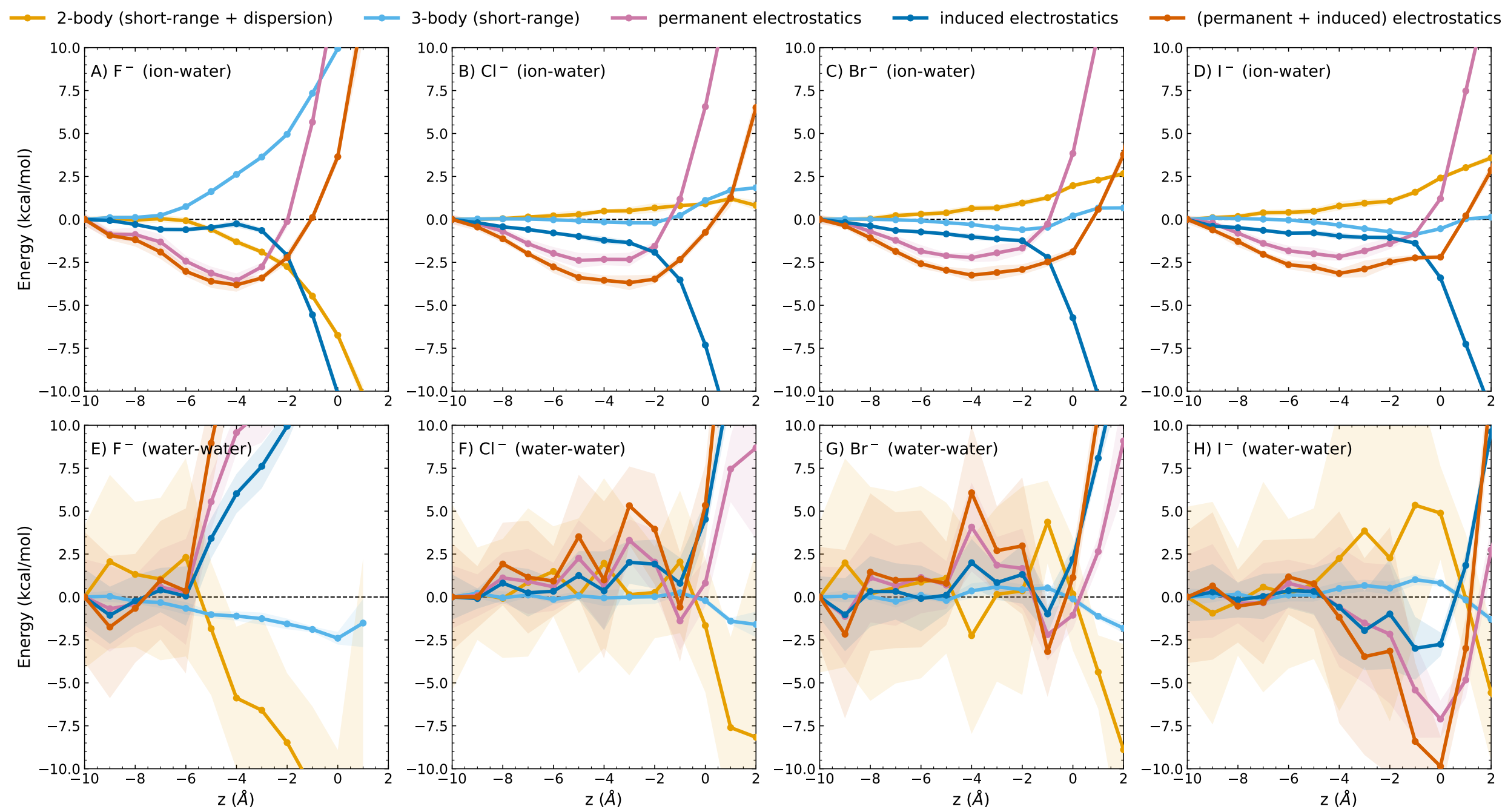

Supplementary Figure 6: Configurational energy contributions calculated with the (2B+3B+NB)-MB-nrg potentials along the surface normal referenced to the WCI[25] at $z = 0$. Top row (A–D): ion–water contributions; bottom row (E–H): water–water contributions. Negative values denote stabilization relative to the bulk reference. Curves resolve 2-body (exchange–repulsion + charge penetration/transfer + dispersion), short-range 3-body, and permanent and induced electrostatics, and permanent and induced electrostatics, along with the total (permanent + induced) electrostatics, as indicated in the legends. All trends qualitatively mirror those referenced to the GDS in Figure 3. However, when referenced to the instantaneous WCI, ion–water electrostatic contributions are relatively larger in the subsurface region and vary more abruptly near $z = 0$ (top row). On the other hand, water–water contributions (bottom row) exhibit noticeably larger noise, reflecting local interfacial fluctuations and reduced configurational averaging. Shaded bands denote the 95% confidence intervals.

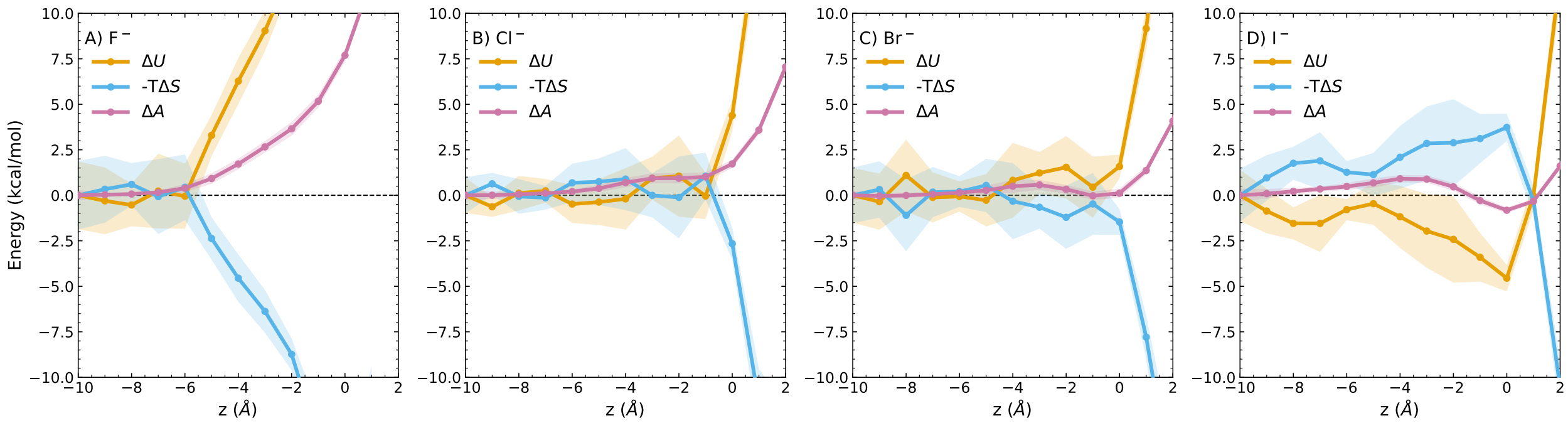

Supplementary Figure 7: Free-energy decompositions calculated with the (2B+3B+NB)-MB-nrg potentials along the surface-normal coordinate referenced to the WCI[25] at $z = 0$. Helmholtz free energy ΔA (reddish purple) is decomposed into the configurational (potential) energy ΔU (orange) and the entropic contribution $-T\Delta S$ (sky blue) for (A) F^-, (B) Cl^-, (C) Br^-, and (D) I^-. Negative values indicate stabilization relative to the bulk reference. For F^-, Cl^-, and Br^-, ΔU becomes positive near the interface and dominates over the entropic term, yielding $\Delta A \gtrsim 0$ across the interfacial region. For I^-, a distinct minimum in ΔU at the WCI is partially offset by an entropic penalty (positive)$-T\Delta S > 0$), compressing the net free-energy well to a shallow minimum of order $k_B T$ near $z = 0$. Compared to the GDS-referenced profiles in Figure 4, the near-surface variations are more abrupt when referenced to the instantaneous WCI, reflecting local interfacial fluctuations. Shaded bands denote the 95% confidence intervals.

Supplementary Note 5: Gibbs dividing surface (GDS): TTM-nrg

This section presents the GDS-referenced configurational-energy and free-energy decompositions obtained with the TTM-nrg potential. Supplementary Figures S8–S10 show that, relative to the fully many-body (2B+3B+NB)-MB-nrg results, TTM-nrg predicts systematically more favorable near-surface solvent-response contributions and consequently stronger interfacial stabilization for the heavier halides. These analyses help clarify why classical polarizable descriptions recover pronounced adsorption for I^- and smaller minima for Br^- and Cl^-.

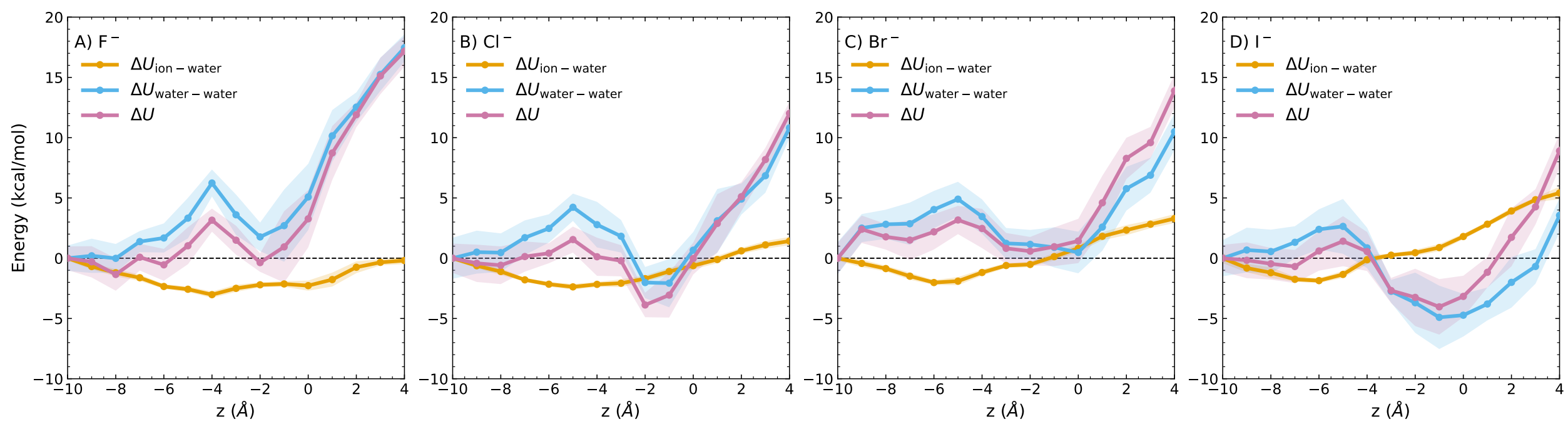

Supplementary Figure 8: Configurational energy decompositions calculated with the TTM-nrg potentials along the surface-normal coordinate referenced to the GDS at $z = 0$. Shown are the ion–water contribution $\Delta U_{\text{ion-water}}$ (orange), the water–water contribution $\Delta U_{\text{water-water}}$ (sky blue), and their sum ΔU (reddish purple) for (A) F^-, (B) Cl^-, (C) Br^-, and (D) I^-. Negative values denote stabilization relative to the bulk reference. All ions display a relatively broad subsurface minimum in $\Delta U_{\text{ion-water}}$. Compared to the (2B+3B+NB)-MB-nrg results in Figure 2, $\Delta U_{\text{water-water}}$ for F^- is non-monotonic, exhibiting a local minimum near the GDS. Similar trends to (2B+3B+NB)-MB-nrg are instead found for Cl^-, Br^-, and I^-, although TTM-nrg systematically predicts more favorable water–water contributions as the ions approach the GDS. Shaded bands denote the 95% confidence intervals.

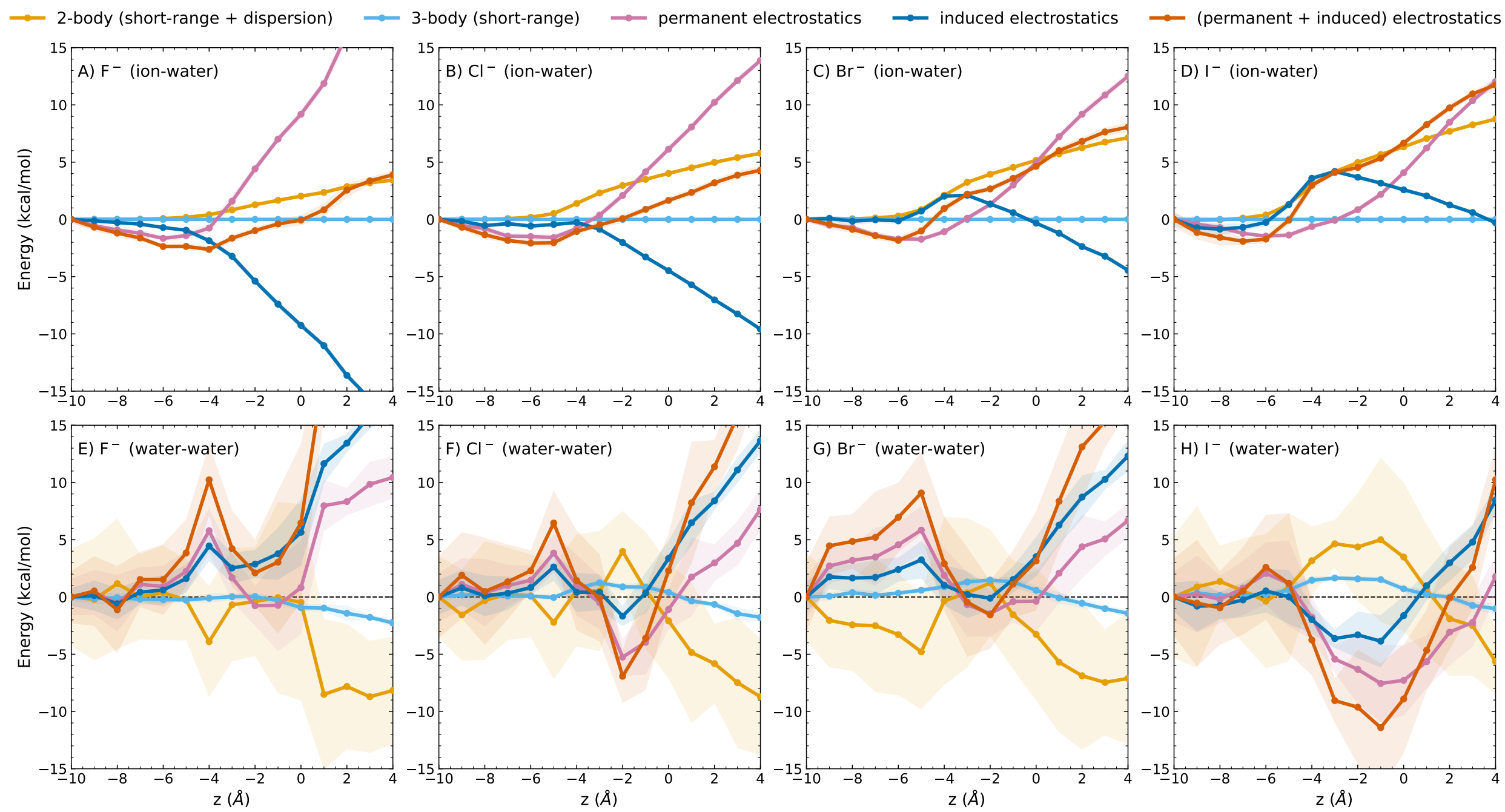

Supplementary Figure 9: Configurational energy contributions calculated with the TTM-nrg potentials along the surface normal referenced to the GDS at $z = 0$. Top row (A–D): ion–water contributions; bottom row (E–H): water–water contributions. Curves resolve 2-body (exchange–repulsion + charge penetration/transfer + dispersion), short-range 3-body, and permanent and induced electrostatics, along with the total (permanent + induced) electrostatics, as indicated in the legends. Negative values denote stabilization relative to the bulk reference. Relative to the (2B+3B+NB)-MB-nrg results in Figure 3, induced electrostatics is favorable across the entire slab only for F^- and Cl^-, but becomes positive in the subsurface region for Br^- and I^-, indicating that the TTM-nrg potentials predict different interfacial ion hydration structures compared to (2B+3B+NB)-MB-nrg. All TTM-nrg water–water terms exhibit the same trends seen for (2B+3B+NB)-MB-nrg, although they are systematically more favorable. Shaded bands denote the 95% confidence intervals.

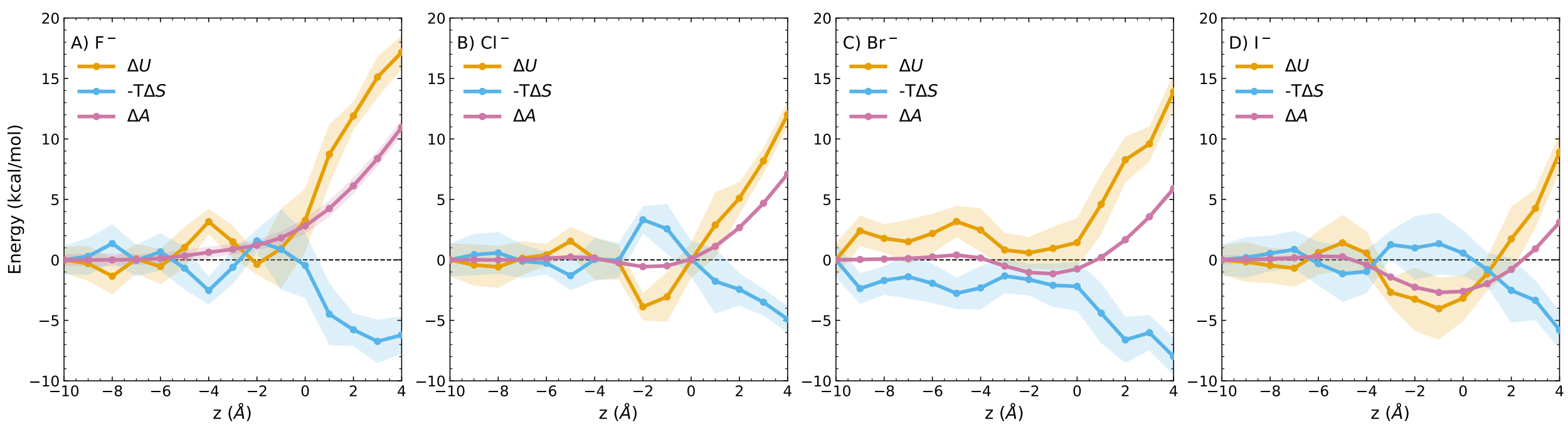

Supplementary Figure 10: Free-energy decompositions calculated with the TTM-nrg potentials along the surface-normal coordinate referenced to the GDS at $z = 0$. Helmholtz free energy ΔA (reddish purple) is decomposed into the total configurational (potential) energy ΔU (orange) and the total entropic contribution $-T\Delta S$ (sky blue) for (A) F^-, (B) Cl^-, (C) Br^-, and (D) I^-. Negative values indicate stabilization relative to the bulk reference. While ΔU remains positive across the entire interfacial region for F^-, it exhibits a minimum near the GDS for the other halides, which is only partially compensated by the entropic term. As a result, TTM-nrg predicts $\Delta A < 0$ near $z = 0$ not only for I^- but also for Cl^- and Br^-. Shaded bands denote the 95% confidence intervals.

Supplementary Note 6: Gibbs dividing surface (GDS): (2B)-MB-nrg

This section reports the corresponding GDS-referenced analyses for the purely pairwise-additive (2B)-MB-nrg potential. Supplementary Figures S11–S13 show that, in the absence of many-body polarization and explicit short-range 3-body terms, the total configurational energy remains near or above the bulk reference across the interfacial region for all halides, and no interfacial minimum appears in the free-energy profiles. These results are consistent with the generic ion exclusion expected from a predominantly pairwise-additive description.

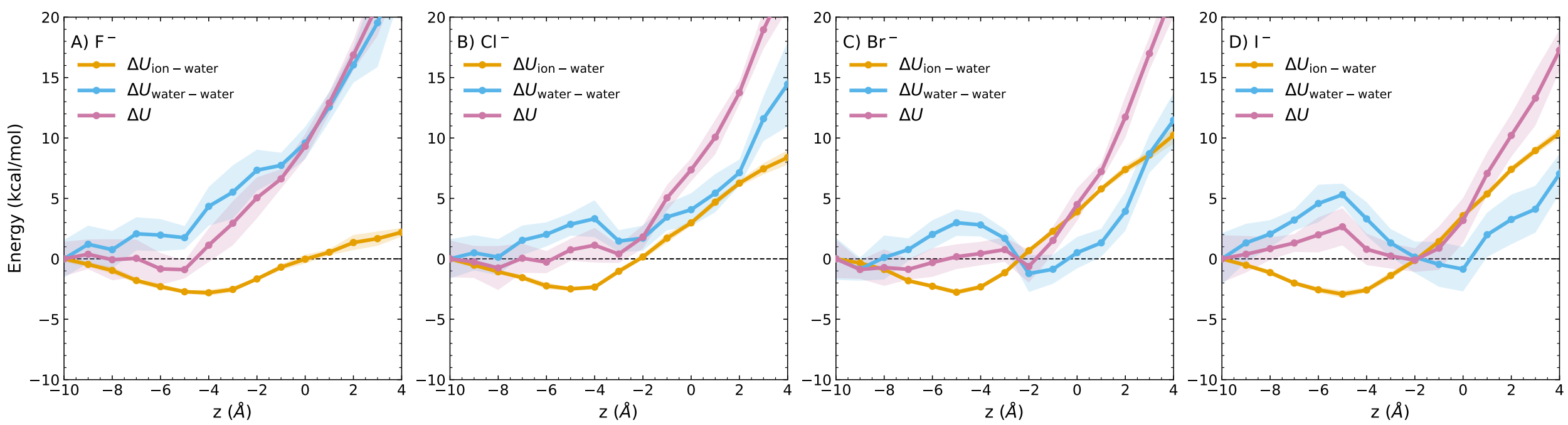

Supplementary Figure 11: Configurational energy decompositions calculated with the (2B)-MB-nrg potentials along the surface-normal coordinate referenced to the GDS at $z = 0$. Shown are the ion–water contribution $\Delta U_{\text{ion}-\text{water}}$ (orange), the water–water contribution $\Delta U_{\text{water}-\text{water}}$ (sky blue), and their sum ΔU (reddish purple) for (A) F^-, (B) Cl^-, (C) Br^-, and (D) I^-. Negative values denote stabilization relative to the bulk reference. All ions display a subsurface minimum in $\Delta U_{\text{ion}-\text{water}}$, but the wells are slightly shallower than in the (2B+3B+NB)-MB-nrg case (Figure 2) and shift slightly toward the bulk. $\Delta U_{\text{water}-\text{water}}$ calculated with (2B)-MB-nrg differs quantitatively from the (2B+3B+NB)-MB-nrg predictions. In particular, while $\Delta U_{\text{water}-\text{water}}$ for (2B)-MB-nrg increases nearly monotonically and becomes very positive near the GDS for F^-, it is increasingly non-monotonic for Cl^-, Br^-, and I^-, with no distinct minimum at the GDS even for iodide. As a result, in the (2B)-MB-nrg case, the total configurational energy ΔU remains near or above zero across the interfacial region for all ions, including I^-. Shaded bands denote the 95% confidence intervals.

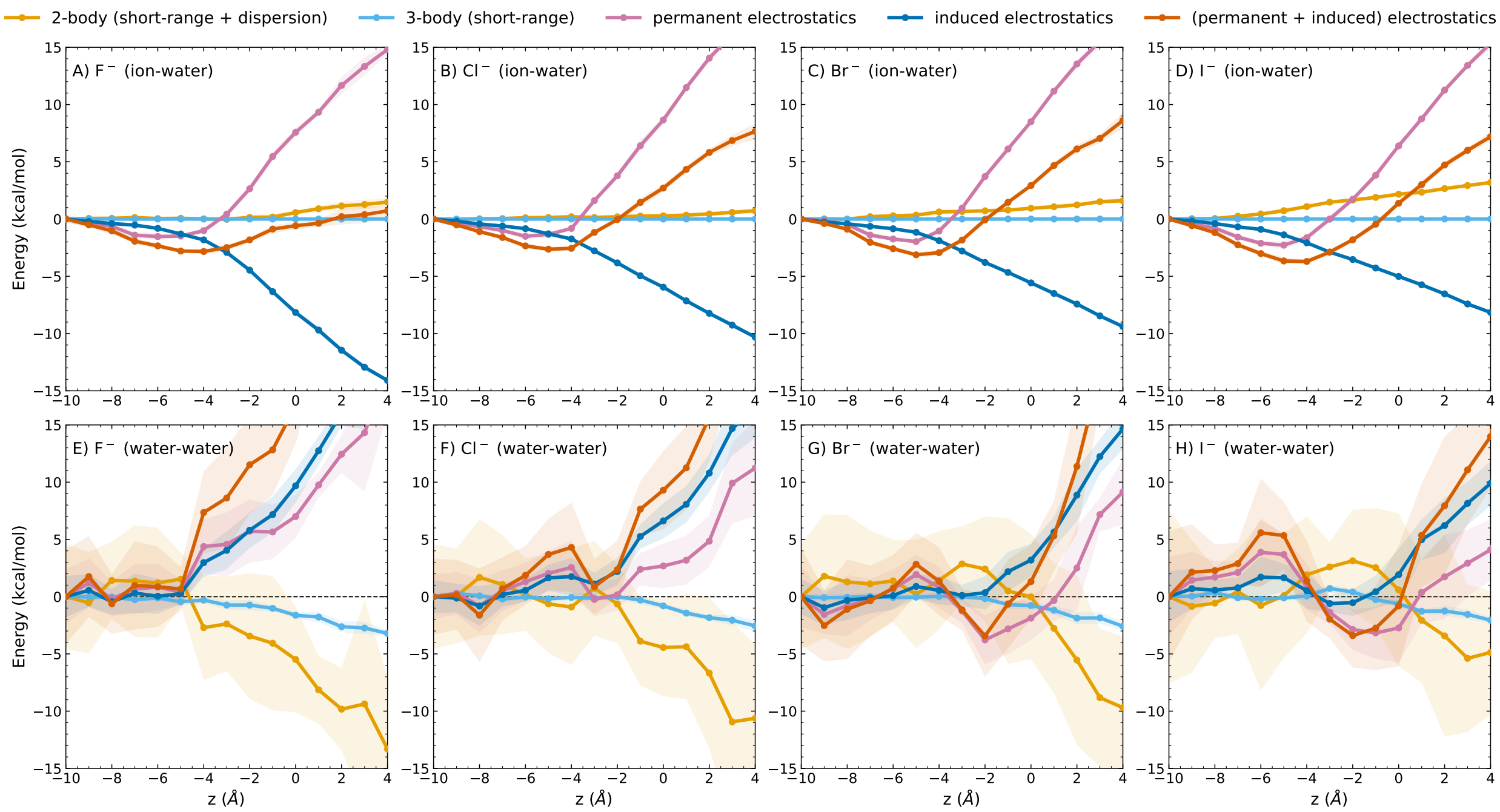

Supplementary Figure 12: Configurational energy contributions calculated with the (2B)-MB-nrg potentials along the surface normal referenced to the GDS at $z = 0$. Top row (A–D): ion–water contributions; bottom row (E–H): water–water contributions. Curves resolve 2-body (exchange–repulsion + charge penetration/transfer + dispersion), short-range 3-body, and permanent and induced electrostatics, induced electrostatics, as well as the total (permanent + induced) electrostatics, as indicated in the legends. Negative values denote stabilization relative to the bulk reference. By construction, the short-range 3-body ion–water term in (2B)-MB-nrg is zero (top-row brown curves), so the ion–water balance is governed by 2-body interactions and electrostatics. Relative to the (2B+3B+NB)-MB-nrg results in Figure 3, the subsurface 2-body stabilization is reduced and the 2-body term becomes increasingly repulsive as the ion approaches the GDS (especially for Br^- and I^-). The total ion–water electrostatics (permanent + induced) remains positive at the GDS for all ions. On the solvent side, water–water electrostatics does not develop the iodide-specific minimum resolved with the fully many-body (2B+3B+NB)-MB-nrg potential, and the 2-body term is broader and more weakly stabilizing near the GDS. Shaded bands denote the 95% confidence intervals.

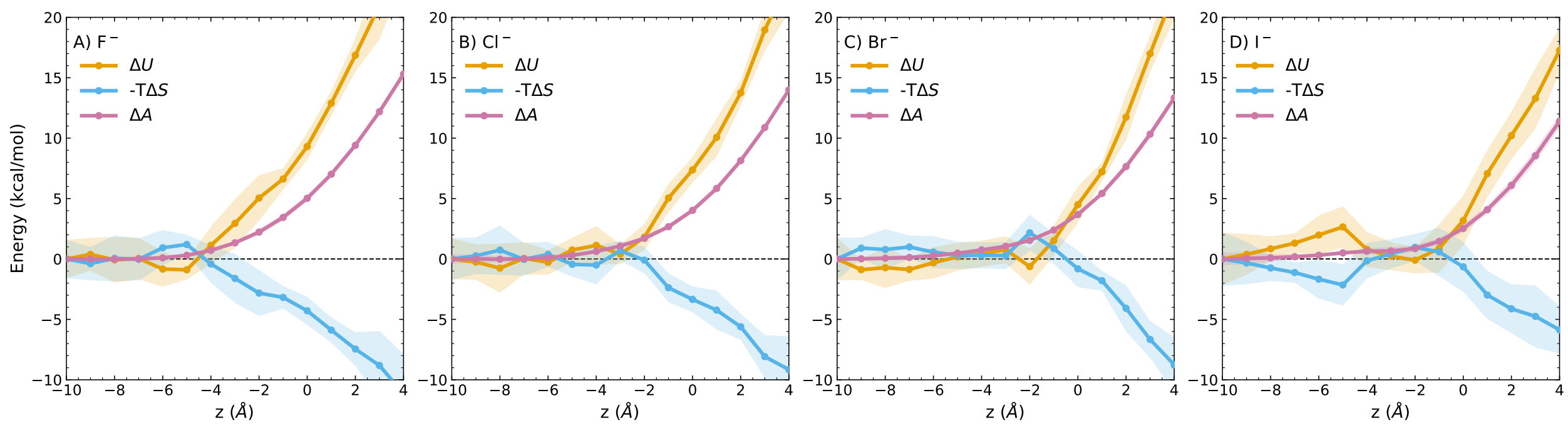

Supplementary Figure 13: Free-energy decompositions calculated with the (2B)-MB-nrg potentials along the surface-normal coordinate referenced to the GDS at $z = 0$. Helmholtz free energy ΔA (reddish purple) is decomposed into the configurational (potential) energy ΔU (orange) and the entropic contribution $-T\Delta S$ (sky blue) for (A) F^-, (B) Cl^-, (C) Br^-, and (D) I^-. Negative values indicate stabilization relative to the bulk reference. For all ions, $-T\Delta S$ becomes negative near the interface (entropic stabilization), but ΔU rises sharply and dominates, yielding $\Delta A \gtrsim 0$ across the entire interfacial region. Unlike the fully many-body (2B+3B+NB)-MB-nrg potential in Figure 4, I^- does not exhibit a distinct minimum in ΔU at the GDS and the entropic term remains stabilizing rather than penalizing. Consequently, (2B)-MB-nrg does not predict interfacial minima in ΔA for any of the halides. Overall, ΔU is more positive relative to the (2B+3B+NB)-MB-nrg results in Figure 4, consistent with the absence of explicit 3-body and many-body polarization terms in (2B)-MB-nrg. Shaded bands denote the 95% confidence intervals.

Supplementary Note 7: Gibbs dividing surface (GDS): (2B+NB)-MB-nrg

This section presents the GDS-referenced decompositions obtained with the (2B+NB)-MB-nrg potential, which combines an explicit 2-body machine-learned ion–water term with classical many-body polarization. Supplementary Figures S14–S16 show that adding polarization generates shallow local minima near the GDS for the heavier halides, but these remain insufficient to produce true thermodynamic stabilization relative to the bulk. Comparison with the fully many-body (2B+3B+NB)-MB-nrg results thus highlights the critical role of explicit short-range 3-body effects in compressing halide surface propensity to the weak, iodide-specific regime discussed in the main text.

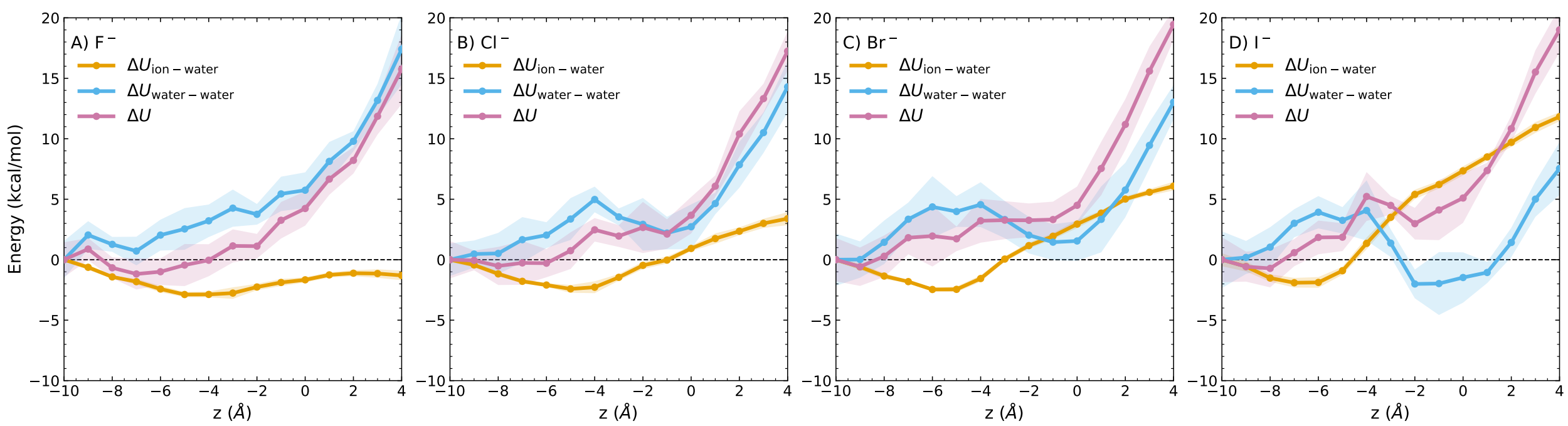

Supplementary Figure 14: Configurational energy decompositions calculated with the (2B+NB)-MB-nrg potentials along the surface-normal coordinate referenced to the GDS at $z = 0$. Shown are the ion–water contribution $\Delta U_{\mathrm{ion-water}}$ (orange), the water–water contribution $\Delta U_{\mathrm{water-water}}$ (sky blue), and their sum ΔU (reddish purple) for (A) F^-, (B) Cl^-, (C) Br^-, and (D) I^-. Negative values denote stabilization relative to the bulk reference. All ions display a subsurface minimum in $\Delta U_{\mathrm{ion-water}}$, but the wells are slightly shallower than in the (2B+3B+NB)-MB-nrg case (Figure 2) and shift slightly toward the bulk. $\Delta U_{\mathrm{water-water}}$ calculated with (2B+NB)-MB-nrg differs quantitatively from the (2B+3B+NB)-MB-nrg predictions. In particular, while $\Delta U_{\mathrm{water-water}}$ for (2B+NB)-MB-nrg increases nearly monotonically and becomes very positive near the GDS for F^-, it is increasingly non-monotonic for Cl^-, Br^-, and I^-, displaying shallow minima at the GDS, which, however, are only local for Cl^- and Br^-. Due to the very repulsive $\Delta U_{\mathrm{ion-water}}$ contribution, which dominates over $\Delta U_{\mathrm{water-water}}$ near the GDS, the total configurational energy ΔU predicted by (2B+NB)-MB-nrg remains near or above zero across the interfacial region for all ions, including I^- for which it only exhibits a local minimum above the bulk reference value. Shaded bands denote the 95% confidence intervals.

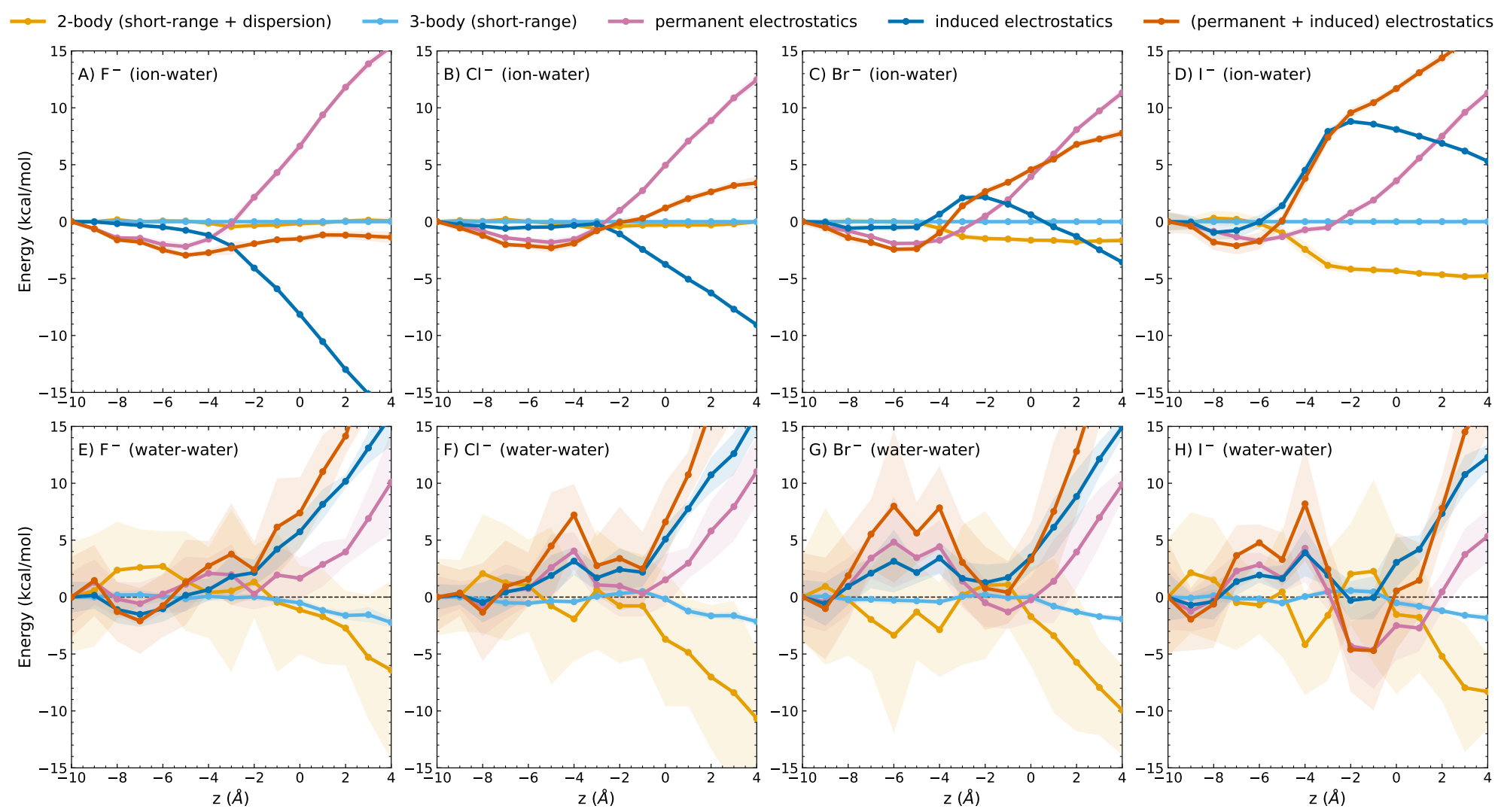

Supplementary Figure 15: Configurational energy contributions calculated with the (2B+NB)-MB-nrg potentials along the surface normal referenced to the GDS at $z = 0$. Top row (A–D): ion–water contributions; bottom row (E–H): water–water contributions. Curves resolve 2-body (exchange–repulsion + charge penetration/transfer + dispersion), short-range 3-body, and permanent and induced electrostatics, as well as total (permanent + induced) electrostatics, as indicated in the legends. Negative values denote stabilization relative to the bulk reference. By construction, the short-range 3-body ion–water term is zero. Relative to the fully many-body (2B+3B+NB)-MB-nrg results in Figure 3, the ion–water 2-body term remains stabilizing as the ion approaches the interface for the heavier halides (especially I^-), whereas in the (2B+3B+NB)-MB-nrg case it becomes increasingly repulsive. Contrary to (2B+3B+NB)-MB-nrg but similar to TTM-nrg, the ion–water induced electrostatics becomes positive as the heavier halides (Br^- and I^-) approach the GDS, resulting in a significantly more positive total ion–water electrostatics near the GDS than in the (2B+3B+NB)-MB-nrg case. On the solvent side, water–water electrostatics lacks the well-defined iodide-specific minimum at the GDS resolved with the fully many-body (2B+3B+NB)-MB-nrg potential and instead exhibits only a shallow modulation near the interface. The water–water 2-body term is generally stabilizing but largely compensated by the electrostatics term, leaving limited net solvent-driven stabilization. Shaded bands denote the 95% confidence intervals.

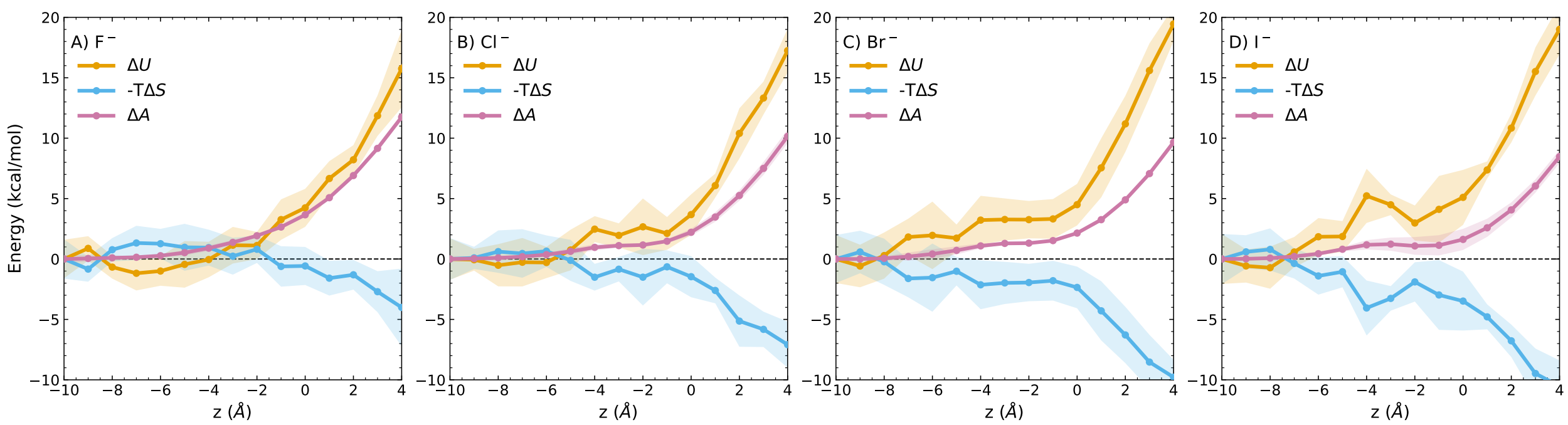

Supplementary Figure 16: Free-energy decompositions calculated with the (2B+NB)-MB-nrg potentials along the surface-normal coordinate referenced to the GDS at $z = 0$. Helmholtz free energy ΔA (reddish purple) is decomposed into the configurational (potential) energy ΔU (orange) and the entropic contribution $-T\Delta S$ (sky blue) for (A) F^-, (B) Cl^-, (C) Br^-, and (D) I^-. Negative values indicate stabilization relative to the bulk reference. For all halides, ΔU rises and dominates over the entropic term, yielding $\Delta A \gtrsim 0$ across the entire interfacial region, with only a shallow, local minimum near the GDS for Br^- and I^-. Compared to the fully many-body (2B+3B+NB)-MB-nrg results in Figure 4, ΔU for I^- does not exhibit the well-defined minimum at the GDS responsible for the shallow minimum of order $\mathrm{k_B}T$ in ΔA near $z = 0$. Shaded bands denote the 95% confidence intervals.

Supplementary Note 8: vSFG spectra at the air/water interface with halide ions

Supplementary Figure 17 shows the heterodyne-detected vibrational sum-frequency generation (HD-vSFG) spectra in the XXZ polarization combination for the air/water interface containing a single halide ion using all four ion–water potentials considered in the manuscript: (A) (2B+3B+NB)-MB-nrg, (B) TTM-nrg, (C) (2B)-MB-nrg, and (D) (2B+NB)-MB-nrg. These spectra are not intended for direct comparison with finite-concentration vSFG measurements,[31] since the present simulations probe the dilute single-ion limit. Nevertheless, the evolution of the interfacial O–H stretch response across the different models provides a complementary spectroscopic signature of the many-body hierarchy observed in the free-energy profiles.

At the neat air/water interface, the positive peak near 3700 cm^{-1} arises from free O–H groups, whereas the negative band in the 3200–3600 cm^{-1} region arises from hydrogen-bonded O–H groups. In the presence of ions, a decrease in the free O–H peak intensity, together with the appearance or enhancement of a positive feature in the hydrogen-bonded region, is consistent with increased ion population at the interface. This spectral response reflects the formation of an asymmetric and incomplete solvation shell around interfacial ions: water molecules remain predominantly on the liquid side of the halide ion and orient their O–H groups toward the halide ion and the vapor phase, without a compensating layer of oppositely oriented water molecules on the air side. This imbalance in water orientation gives rise to a net positive vSFG response in the hydrogen-bonded O–H stretching region.

Consistent with the Helmholtz free-energy profiles shown in Figure 1 of the main text, the vSFG spectra shown in Supplementary Figure 17, computed using TTM-nrg exhibit a positive feature near 3400 cm^{-1}, accompanied by a decrease in the free O–H peak intensity for I^-, Br^-, and Cl^-, indicating an enhanced surface propensity for these ions. In contrast, the vSFG spectra computed using (2B+3B+NB)-MB-nrg show appreciable surface propensity only for I^-, consistent with the shallow iodide minimum in the corresponding PMF. The spectra obtained with the purely pairwise-additive (2B)-MB-nrg do not exhibit a positive hydrogen-bonded O–H feature for any of the halide ions, again consistent with the repulsive PMFs. Finally, the spectra computed with (2B+NB)-MB-nrg show only a weak positive feature near 3400 cm^{-1} for I^- within statistical uncertainty, in line with the shallow local minima seen in the corresponding free energy profile. These results demonstrate that the hierarchy of ion surface propensity inferred from the PMFs is also reflected in the interfacial O–H stretch vibrational response.

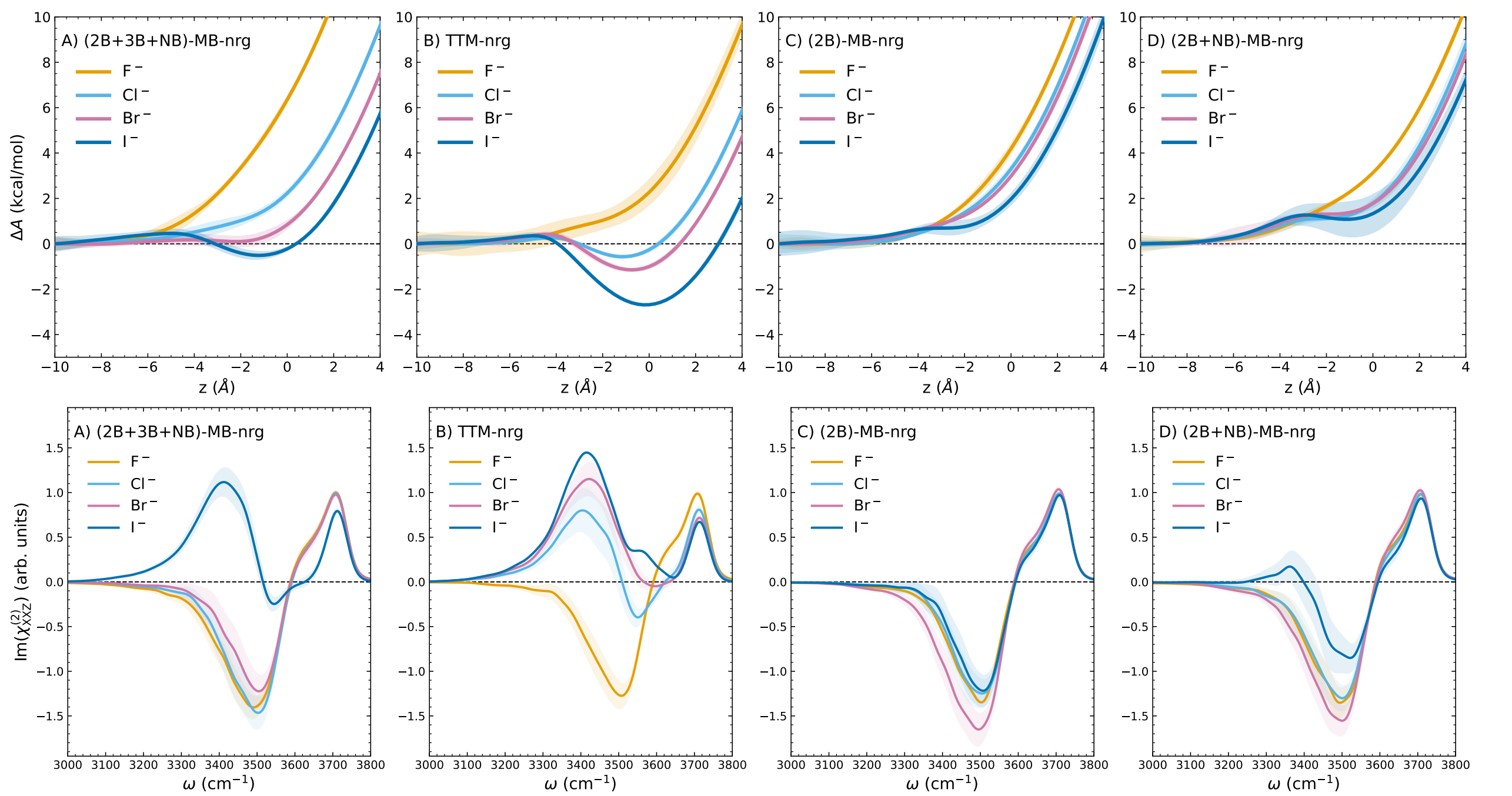

Supplementary Figure 17: Helmholtz free-energy profiles (upper panel) and HD-vSFG spectra in the XXZ polarization combination (lower panel) calculated for the single-halide air/water interface using different ion–water PEFs: (A) (2B+3B+NB)-MB-nrg, (B) TTM-nrg, (C) (2B)-MB-nrg, and (D) (2B+NB)-MB-nrg. The many-body dipole moment surface (MB-μ) and polarizability surface (MB-α) were used to compute the dipole moments and polarizability tensors.[32] The second-order susceptibility $\chi^{(2)}_{ijk}(\omega)$ was calculated from the Fourier transform of the dipole moment–polarizability time-auto-correlation function.[33] Nonresonant background contributions and Fresnel factors were not included. The spectra were red-shifted by 175 cm^{-1} to approximately account for nuclear quantum effects.[34] Each spectrum was weighted according to the corresponding PMF, such that configurations in different interfacial regions contribute in proportion to the equilibrium probability of finding the ion at that position. Shaded bands denote the 95% confidence intervals estimated from approximately 100 independent 50-ps trajectories propagated in the NVE ensemble.

Supplementary References

1. Nesbet, R. K. *Atomic Bethe-Goldstone Equations*, 1–34 (John Wiley & Sons, Ltd, 1969).

2. Hankins, D., Moskowitz, J. & Stillinger, F. Water molecule interactions. *J. Chem. Phys.* **53**, 4544–4554 (1970).

3. Babin, V., Leforestier, C. & Paesani, F. Development of a "first principles" water potential with flexible monomers: Dimer potential energy surface, VRT spectrum, and second virial coefficient. *J. Chem. Theory Comput.* **9**, 5395–5403 (2013). URL `http://pubs.acs.org/doi/10.1021/ct400863t`.

4. Babin, V., Medders, G. R. & Paesani, F. Development of a "first principles" water potential with flexible monomers. II: Trimer potential energy surface, third virial coefficient, and small clusters. *J. Chem. Theory Comput.* **10**, 1599–1607 (2014).

5. Medders, G. R., Babin, V. & Paesani, F. Development of a "first-principles" water potential with flexible monomers. III. Liquid phase properties. *J. Chem. Theory Comput.* **10**, 2906–2910 (2014).

6. Zhu, X., Riera, M., Bull-Vulpe, E. F. & Paesani, F. Mb-pol(2023): Sub-chemical accuracy for water simulations from the gas to the liquid phase. *J. Chem. Theory Comput.* **19**, 3551–3566 (2023).

7. Reddy, S. K. *et al.* On the accuracy of the MB-pol many-body potential for water: Interaction energies, vibrational frequencies, and classical thermodynamic and dynamical properties from clusters to liquid water and ice. *J. Chem. Phys.* **145**, 194504 (2016).

8. Palos, E. *et al.* Current status of the MB-pol data-driven many-body potential for predictive simulations of water across different phases. *J. Chem. Theory Comput.* **20**, 9269–9289 (2024).

9. Arismendi-Arrieta, D. J., Riera, M., Bajaj, P., Prosmiti, R. & Paesani, F. i-TTM model for ab initio-based ion–water interaction potentials. 1. Halide–water potential energy functions. *J. Phys. Chem. B* **120**, 1822–1832 (2016).

10. Riera, M., Götz, A. W. & Paesani, F. The i-TTM model for ab initio-based ion–water interaction potentials. II. Alkali metal ion–water potential energy functions. *Phys. Chem. Chem. Phys.* **18**, 30334–30343 (2016).

11. Bajaj, P., Götz, A. W. & Paesani, F. Toward chemical accuracy in the description of ion–water interactions through many-body representations. I. Halide–water dimer potential energy surfaces. *J. Chem. Theory Comput.* **12**, 2698–2705 (2016).

12. Riera, M., Mardirossian, N., Bajaj, P., Götz, A. W. & Paesani, F. Toward chemical accuracy in the description of ion–water interactions through many-body representations. Alkali–water dimer potential energy surfaces. *J. Chem. Phys.* **147**, 161715 (2017).

13. Thole, B. Molecular polarizabilities calculated with a modified dipole interaction. *Chem. Phys.* **59**, 341–350 (1981).

14. Burnham, C., Anick, D., Mankoo, P. & Reiter, G. The vibrational proton potential in bulk liquid water and ice. *J. Chem. Phys.* **128**, 154519 (2008).

15. Riera, M. *et al.* MBX: A many-body energy and force calculator for data-driven many-body simulations. *J. Chem. Phys.* **159**, 054802 (2023).

16. Gupta, S. *et al.* MBX v1.2: Accelerating data-driven many-body molecular dynamics simulations. *J. Chem. Theory Comput.* **21**, 1838–1849 (2025).

17. Becke, A. D. & Johnson, E. R. Exchange-hole dipole moment and the dispersion interaction. *J. Chem. Phys.* **122**, 154104 (2005).

18. Johnson, E. R. & Becke, A. D. A post-Hartree–Fock model of intermolecular interactions. *J. Chem. Phys.* **123**, 024101 (2005).

19. Johnson, E. R. & Becke, A. D. A post-Hartree–Fock model of intermolecular interactions: Inclusion of higher-order corrections. *J. Chem. Phys.* **124**, 174104 (2006).

20. Tang, K. & Toennies, J. P. An improved simple model for the van der Waals potential based on universal damping functions for the dispersion coefficients. *J. Chem. Phys.* **80**, 3726–3741 (1984).

21. Braams, B. J. & Bowman, J. M. Permutationally invariant potential energy surfaces in high dimensionality. *Int. Rev. Phys. Chem.* **28**, 577–606 (2009).

22. Thompson, A. P. *et al.* LAMMPS – A flexible simulation tool for particle-based materials modeling at the atomic, meso, and continuum scales. *Comput. Phys. Commun.* **271**, 108171 (2022).

23. Tribello, G. A., Bonomi, M., Branduardi, D., Camilloni, C. & Bussi, G. PLUMED 2: New feathers for an old bird. *Comp. Phys. Commun.* **185**, 604–613 (2014).

24. Invernizzi, M. & Parrinello, M. Rethinking metadynamics: From bias potentials to probability distributions. *J. Phys. Chem. Lett.* **11**, 2731–2736 (2020).

25. Willard, A. P. & Chandler, D. Instantaneous liquid interfaces. *J. Phys. Chem. B* **114**, 1954–1958 (2010).

26. Klug, J., Triguero, C., Del Pópolo, M. G. & Tribello, G. A. Using intrinsic surfaces to calculate the free-energy change when nanoparticles adsorb on membranes. *J. Phys. Chem. B* **122**, 6417–6422 (2018).

27. Ryckaert, J.-P., Ciccotti, G. & Berendsen, H. J. Numerical integration of the cartesian equations of motion of a system with constraints: Molecular dynamics of n-alkanes. *J. Comp. Phys.* **23**, 327–341 (1977).

28. Shinoda, W., Shiga, M. & Mikami, M. Rapid estimation of elastic constants by molecular dynamics simulation under constant stress. *Phys. Rev. B* **69**, 134103 (2004).

29. Martyna, G. J., Klein, M. L. & Tuckerman, M. Nosé–hoover chains: The canonical ensemble via continuous dynamics. *J. Chem. Phys.* **97**, 2635–2643 (1992).

30. Torrie, G. M. & Valleau, J. P. Nonphysical sampling distributions in monte carlo free-energy estimation: Umbrella sampling. *J. Comp. Phys.* **23**, 187–199 (1977).

31. Litman, Y., Chiang, K.-Y., Seki, T., Nagata, Y. & Bonn, M. Surface stratification determines the interfacial water structure of simple electrolyte solutions. *Nat. Chem.* **16**, 644–650 (2024).

32. Medders, G. R. & Paesani, F. Infrared and raman spectroscopy of liquid water through "first-principles" many-body molecular dynamics. *J. Chem. Theory Comput.* **11**, 1145–1154 (2015).

33. Moberg, D. R., Straight, S. C. & Paesani, F. Temperature dependence of the air/water interface revealed by polarization sensitive sum-frequency generation spectroscopy. *J. Phys. Chem. B* **122**, 4356–4365 (2018).

34. Medders, G. R. & Paesani, F. Dissecting the molecular structure of the air/water interface from quantum simulations of the sum-frequency generation spectrum. *J. Am. Chem. Soc.* **138**, 3912–3919 (2016).
