# Supplementary material for: Many-body interactions resolve halide distribution at the air/water interface
Source: Nat Commun. 2026 Aug 29;17:10313. doi: 10.1038/s41467-026-77205-9 (PMC13620159; doi:10.1038/s41467-026-77205-9)
Supplement: Supplementary file 3 [file 41467_2026_77205_MOESM3_ESM.pdf]

nature portfolio

Peer Review File

Many-Body Interactions Resolve Halide Distributions at the Air/Water Interface

Corresponding Author: Professor Francesco Paesani

This file contains all reviewer reports in order by version, followed by all author rebuttals in order by version.

Version 0:

Reviewer comments:

Reviewer #1

(Remarks to the Author)
The manuscript "Many-body Interactions Resolve Halide Distributions at the Air/Water Interface" by Agnew et al. uses molecular dynamics driven by many-body potentials to understand the affinity of halide ions to the air/water interface. The results reported here show that most ions considered (F-,Cl-,Br-) do not show an enhanced adsorption at the interface, except I-, which shows a very shallow minimum of the order of the thermal energy. It is also shown that introducing many-body (beyond two-body) effects is crucial to unravel these subtle effects. Furthermore, the manuscript explains this behavior through a careful analysis of the free energy curves based on decomposing it into ion-water and water-water energetic contributions, and entropic contributions.

I believe this manuscript is very relevant to the community working on water science and interface phenomena. The highly-accurate simulations shed light on a phenomenon which is hard to understand both with experiments or with computer simulations. Indeed, experiments and simulations seem to have reported conflicting results in the past, and the new data described here will help to understand them. The manuscript is well written and the analysis is thorough. The simulations appear to be expertly performed and the technical details seem sufficient to allow for reproducing them (except for minor points noted below). The conclusions are supported by the data which is presented.

For the reasons stated above, I believe this paper is suitable for Nature Communications. However, I have a few suggestions to improve the manuscript:
1) The methods section does not describe well the choice of collective variable used in the OPES simulations. Please specify in detail the definition of the collective variable. I believe the distance of the ion to the surface was used. However, the exact definition is not trivial due to the need to determine the position of the interface in a continuous and differentiable fashion with respect to the atomic coordinates. Also, I suggest including the PLUMED input in the supplementary material.
2) Please clarify if the trajectories of the OPES multiple walkers reversibly explore the regions shown in the free energy surfaces in the paper. This is essential to understand if the free energy surfaces are converged.
3) OPES does not have many parameters, but BARRIER typically has to be specified. Please report it to ensure reproducibility.
4) The accuracy of the potential with respect to the CCSD(T) reference is key to the conclusions. However, it is not clear how accurate the fitting procedure was. Please report errors in the fitting. Also, discuss how the errors in the fitting would translate to the predictions of the free energies of adsorption to the interface.

Reviewer #2

(Remarks to the Author)
The manuscript entitled “Many-Body Interactions Resolve Halide Distributions at the Air/Water Interface” presents a systematic study of the adsorption behavior of halide ions, specifically Cl-, Br-, and I-, at the air–water interface. In this work, the authors incorporate higher-order many-body interaction terms into the potential energy and force calculations, and demonstrate that these higher-order contributions qualitatively alter the adsorption mechanisms predicted by classical force fields and earlier versions of MB potentials that neglected such terms. In particular, the revised physical picture appears to affect Cl- and Br- more significantly.
Overall, the manuscript is reasonably well executed, and the conclusions are supported by generally sound arguments. That said, I do not believe the present work reaches the level of breakthrough and novelty typically expected for publication in

Nature Communications. In my opinion, the study would be more suitable for a specialized journal such as Scientific Reports. My comments are listed below:

1. The work reads primarily as an application of an incremental extension of the existing MB-potential framework based on semi-empirical many-body methods. In the present case, the authors demonstrate that higher-order many-body terms are important for describing the adsorption of halide ions at the air–water interface. As such, the work appears more as an application of an incremental improvement of the MB-pol methodology, rather than a fundamentally new conceptual advance.

2. From the perspective of materials and chemical physics, the analyses and discussions are not sufficiently deep or broad. Most of the presented results and analyses are restricted to adsorption-related properties, such as free-energy profiles and configurational energetics at the interface. A broader analysis of the underlying microscopic physics or implications for interfacial chemistry would strengthen the impact of the work.

3. More importantly, the simulation results are not supported by calculations of experimentally measurable spectroscopic observables, such as sum-frequency generation spectroscopy (SFG or vSFG). Simulations of such spectra would allow for more direct comparison with experiments and would substantially strengthen the credibility and significance of the conclusions.

4. One minor issue: in the terminology "2B+3B+NB-nrg", the meaning of the term "NB" does not appear to be clearly defined in the main text. The authors should clarify this notation upon first introduction.

Reviewer #3

(Remarks to the Author)
The authors use a data-driven many-body simulation potential to determine the interfacial thermodynamics of halide ions at the air-water interface. They find a weak adsorption of iodide, which by energy decomposition they show to be due to water cavity/reorganization contribution that compensates ion–water desolvation. The paper is nicely written, ion-specific effects at interfaces are important and still pose a number of questions, the numerical framework developed and used by the authors is sound, I therefore recommend publication of the paper and only have a few comments on the presentation.

- The authors write "In general, molecular dynamics (MD) simulations with non-polarizable force fields yield no surface affinity …" which is not really true. In the rather old review Ref. 5 non-polarizable force fields were shown to give rise to surface adsorption, in the more recent paper "Orientation-Induced Adsorption of Hydrated Protons at the Air–Water Interface", Mamatkulov et al, Angew. Chem. Int. Ed. 2017, 56, 15846 –15851, MD simulations with non-polarizable force fields for hydronium, hydroxide, sodium, chloride, that were optimized with respect to bulk ionic properties (solvation free energies and activities), were shown to yield pronounced adsorption for hydronium and to give rise to a surface tension for HCl that in fact decreases with increasing electrolyte concentration in quantitative agreement with experimental data.
- Related to this, the rather pronounced decrease of the HCl surface tension of about 0.25 mJ/m^2 per mol/l of added HCl is caused by a minimum in the PMF of hydronium of about 1 kBT. As the authors correctly note, the surface tension of e.g. NaI solutions increases with added electrolyte concentration but with a slope that is reduced compared to NaCl solutions: The increase of NaCl is about 1.5 mJ/m^2 per mol/l while the increase of NaI is about 1.0 mJ/m^2 per mol/l (from Jarvis Scheimann 1968). The depth of the free energy profile obtained for iodide by the authors in Fig. 2a is about 0.25 kcal/mol which converts to about 1 kJ/mol or 0.5 kBT. It seems that the minimum is too deep compared to experimental surface tension data for iodide electrolytes. Could the authors estimate the surface tension from the expected interfacial excess and compare with experimental data or at least comment on this?
- The authors in their discussion and interpretation repeat an old argument according to which the polarizability of ions furnishes a natural mechanism for interfacial propensity. I do not see how such a mechanism is obtained from basic dielectric theory: Firstly, the electronic polarizability of iodide is indeed larger than that of chloride, which is simply a size effect, since the polarizability of atoms scales with their volume. However, the low-frequency excess polarizability of an ion in an aqueous solution is negative, since an ion replaces water that has a much larger low-frequency polarizability due to its permanent dipole moment. Secondly, the polarizability of an object translates to an interfacial propensity only for a charged interface, but not to a neutral interface. The authors correctly point out that the interaction of an ion with an interface is dictated by solvation effects, this is supported by semi-quantitative modeling of ion-specific interfacial interaction using solvation theory in Ref. 5. I would adapt the wording slightly in the introduction.

Version 1:

Reviewer comments:

Reviewer #1

(Remarks to the Author)
The authors have replied to my comments and I believe the current version of the manuscript is suitable for publication in Nature Communications.

Reviewer #2

(Remarks to the Author)
In the response letter and the revised manuscript, I believe the authors have only partially addressed my previous comments and concerns. While I appreciate the additional analyses and discussions, I remain unconvinced that the manuscript meets the standard for publication in Nature Communications. In my opinion, the work does not provide a sufficient level of conceptual novelty, mechanistic insight, or experimental validation to justify publication in this journal. My detailed comments are as follows:

1. With regard to the novelty of the work, the authors have not provided sufficiently convincing arguments in their response. In fact, they explicitly acknowledge that "the methodological framework has already been developed and validated in previous work." This statement itself suggests that the present manuscript primarily represents an application of an existing computational methodology to a new system. While such applications can certainly be scientifically valuable, the current study does not provide sufficiently compelling evidence that the resulting theoretical predictions constitute a significant conceptual advance or that the proposed adsorption mechanism has been experimentally validated.

2. The manuscript does not provide direct experimental support for its central mechanistic conclusion. Although theoretical HD-vSFG spectra are presented in the revised manuscript, these spectra remain purely theoretical and are neither quantitatively compared with experimental measurements nor calculated under realistic experimental conditions. The simulations consider only a single halide ion at the air/water interface and neglect the presence of counter ions, finite electrolyte concentrations, ion pairing, and collective interfacial effects that are inevitably present in experimental systems. Consequently, the calculated spectra should be regarded as theoretical predictions rather than experimental validation of the proposed adsorption mechanism. Since the principal conclusion—that explicit short-range three-body quantum many-body interactions fundamentally suppress the adsorption of Cl^- and Br^- while leaving only a shallow adsorption minimum for I^-—has not been directly corroborated by experiment, the manuscript remains primarily a computational study. In my view, this substantially limits both the impact and the significance of the work, particularly for a journal such as Nature Communications, where strong experimental support or compelling validation of new theoretical predictions is generally expected.

3. One of the central claims of this manuscript is that explicit short-range three-body quantum many-body interactions qualitatively suppress the adsorption of Cl^- and Br^- while leaving only a shallow adsorption minimum for I^-. Although the simulations clearly demonstrate this change in the free-energy profiles, the manuscript does not provide a convincing microscopic explanation for the underlying physical mechanism. The analyses presented largely describe what changes after the inclusion of the three-body interactions, but they do not explain why these interactions fundamentally alter the adsorption behavior of different halide ions. In particular, the manuscript does not establish a clear causal relationship between the explicit three-body quantum effects (e.g., exchange repulsion, charge penetration, and charge transfer) and the resulting changes in water reorganization, hydrogen-bond structure, or interfacial thermodynamics. Consequently, the work remains largely descriptive and computational rather than providing new mechanistic insight into the origin of the revised adsorption behavior. In my opinion, this lack of physical understanding substantially limits the conceptual advance of the study and falls short of the level of mechanistic insight expected for publication in Nature Communications.

Reviewer #3

(Remarks to the Author)
The authors have adequately addressed all my comments and the paper is ready for publication.

Open Access This Peer Review File is licensed under a Creative Commons Attribution 4.0 International License, which permits use, sharing, adaptation, distribution and reproduction in any medium or format, as long as you give appropriate credit to the original author(s) and the source, provide a link to the Creative Commons license, and indicate if changes were made.
In cases where reviewers are anonymous, credit should be given to 'Anonymous Referee' and the source.
The images or other third party material in this Peer Review File are included in the article's Creative Commons license, unless indicated otherwise in a credit line to the material. If material is not included in the article's Creative Commons license and your intended use is not permitted by statutory regulation or exceeds the permitted use, you will need to obtain permission directly from the copyright holder.
To view a copy of this license, visit https://creativecommons.org/licenses/by/4.0/

Response to Reviewer 1

The manuscript “Many-body Interactions Resolve Halide Distributions at the Air/Water Interface” by Agnew et al. uses molecular dynamics driven by many-body potentials to understand the affinity of halide ions to the air/water interface. The results reported here show that most ions considered (F-,Cl-,Br-) do not show an enhanced adsorption at the interface, except I-, which shows a very shallow minimum of the order of the thermal energy. It is also shown that introducing many-body (beyond two-body) effects is crucial to unravel these subtle effects. Furthermore, the manuscript explains this behavior through a careful analysis of the free energy curves based on decomposing it into ion-water and water-water energetic contributions, and entropic contributions. I believe this manuscript is very relevant to the community working on water science and interface phenomena. The highly-accurate simulations shed light on a phenomenon which is hard to understand both with experiments or with computer simulations. Indeed, experiments and simulations seem to have reported conflicting results in the past, and the new data described here will help to understand them. The manuscript is well written and the analysis is thorough. The simulations appear to be expertly performed and the technical details seem sufficient to allow for reproducing them (except for minor points noted below). The conclusions are supported by the data which is presented. For the reasons stated above, I believe this paper is suitable for Nature Communications. However, I have a few suggestions to improve the manuscript:

We thank the Reviewer for their detailed assessment of our manuscript and for supporting its publication in Nature Communications.

1. The methods section does not describe well the choice of collective variable used in the OPES simulations. Please specify in detail the definition of the collective variable. I believe the distance of the ion to the surface was used. However, the exact definition is not trivial due to the need to determine the position of the interface in a continuous and differentiable fashion with respect to the atomic coordinates. Also, I suggest including the PLUMED input in the supplementary material.

We thank the Reviewer for this suggestion. The ion distance from Gibbs dividing surface was determined from the ion's position relative to the water center of mass and the average water slab thickness, which was approximately 38 Å. Since the water center of mass defines the midpoint of the slab, the ion distance from the Gibbs dividing surface was calculated as ($d_{\text{ion}-\text{GDS}} = d_{\text{ion}-\text{COM}} - 19$ Å), where 19 Å represents half of the average simulation slab thickness. The ion distance from the Willard-Chandler interface is instead computed using the DISTANCE_FROM_CONTOUR collective variable. The PLUMED input for these OPES simulations has been added to the Supplemental Information.

Revision: In the revised manuscript, additional descriptions about the collective variables and the PLUMED input used in the OPES simulations have been included in the Supplementary Information as Figure R1:

> The instantaneous Willard-Chandler interface[25] was calculated using the DISTANCE_FROM_CONTOUR implementation within PLUMED.[26] The PLUMED input used for the OPES_METAD is included as Supplementary Figure 1.

```
UNITS LENGTH=A ENERGY=kcal/mol TIME=ps

# w is the center of mass of bulk water
COM ATOMS=2-1534:3 LABEL=w NOPBC

# calculate distance for GDS
cl: GROUP ATOMS=1
dd: DISTANCE ATOMS=w,cl COMPONENTS NOPBC

md: OPES_METAD ARG=dd.z PACE=1000 BARRIER=20 TEMP=298 WALKERS_MPI
lw: LOWER_WALLS ARG=dd.z AT=9.0 KAPPA=5.0 EXP=2

# calculate contour for Willard-Chandler interface
id1: GROUP ATOMS=2-1534:3
dc: DISTANCE_FROM_CONTOUR POSITIONS=id1 ATOM=1 BANDWIDTH=2.4,2.4,2.4 DIR=z CONTOUR=0.016

bias: COMBINE ARG=*.bias PERIODIC=NO
PRINT STRIDE=50 ARG=dc.*,md.*,dd.z,bias,lw.bias FILE=COLVAR

FLUSH STRIDE=1000
```

Figure R1: PLUMED input used for the OPES simulations.

2. Please clarify if the trajectories of the OPES multiple walkers reversibly explore the regions shown in the free energy surfaces in the paper. This is essential to understand if the free energy surfaces are converged.

We thank the Reviewer for this feedback. The convergence was monitored by plotting the distribution of the walkers and ensuring that the walkers reversibly explored the free energy surface. In order to illustrate this coverage, we have added Figure R2 to the Supplementary Information (Supplementary Figure S2) that illustrates the distribution of walker distances from the GDS. The OPES multiple walkers do indeed reversibly explore the region of interest multiple times throughout the trajectory.

As an additional validation of the convergence, Supplementary Figure S3 also compares the results obtained using OPES simulations against those obtained with umbrella sampling (US) simulations for the (2B+3B+NB)-MB-nrg potential. The similarity of the PMFs calculated using OPES and US simulations provides support for the convergence of the calculated free energy surface.

Revision: In the revised manuscript, we have added a new figure to the Supplementary Information (Supplementary Figure S2) in order to illustrate the OPES multiple walker exploration.

> Supplementary Figure S2 shows the distribution of the ion positions of these walkers throughout the simulation trajectories, indicating that these walkers reversibly traverse the region of interest.

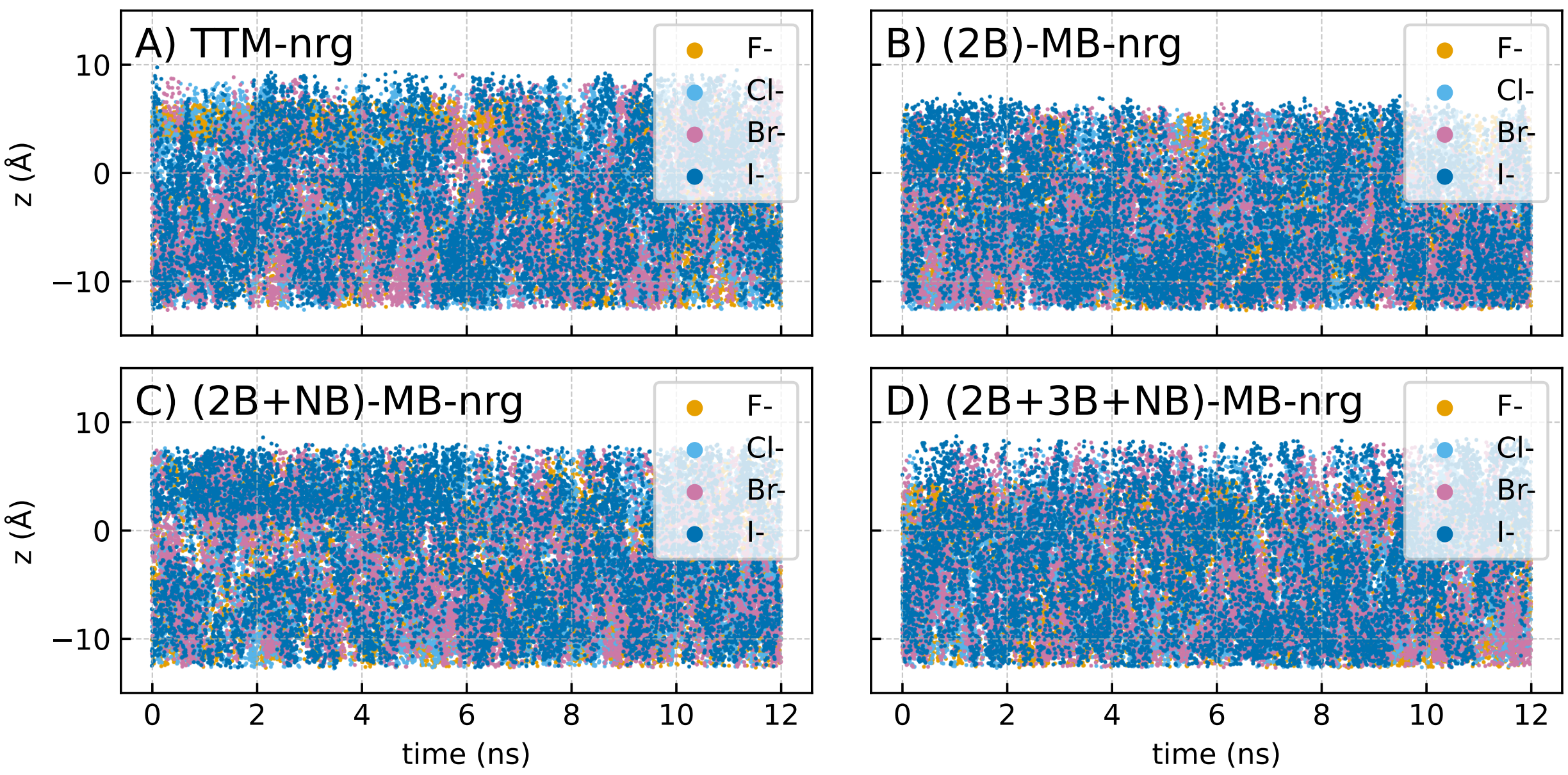

Figure R2: Distribution of ion positions relative to the GDS throughout the simulation trajectories obtained with the TTM-nrg (A), (2B)-MB-nrg (B), (2B+NB)-MB-nrg (C), and (2B+3B+NB)-MB-nrg (D) potentials.

3. OPES does not have many parameters, but BARRIER typically has to be specified. Please report it to ensure reproducibility.

We thank the Reviewer for this suggestion. We used BARRIER=20 kcal/mol so that we could adequately sample the repulsive region when the ion is above the surface. We have added this information about the PLUMED input to the Supplementary Information.

Revision: In the revised Supplementary Information, the BARRIER=20 parameter is explicitly mentioned. The BARRIER parameter is also included as part of the raw PLUMED input in Figure R1 (Supplementary Figure S1):

> The potentials of mean force (PMFs) were obtained from MD simulations performed using the on-the-fly probability enhanced sampling (OPES) method[77] with 8 walkers per simulation and a BARRIER of 20 kcal/mol.

4. The accuracy of the potential with respect to the CCSD(T) reference is key to the conclusions. However, it is not clear how accurate the fitting procedure was. Please report errors in the fitting. Also, discuss how the errors in the fitting would translate to the predictions of the free energies of adsorption to the interface.

We thank the Reviewer for this comment. The original MB-pol papers [J. Chem. Theory Comput. 9, 5395 (2013); J. Chem. Theory Comput. 10, 1599 (2014); J. Chem. Theory Comput. 10, 2906 (2014)] and MB-nrg papers [J. Chem. Theory Comput. 12, 2698 (2016); J. Chem. Phys. 147, 161715 (2017)] provide the full details of the training sets, fitting procedures, and functional forms used for the water and ion–water potentials used in this work.

In particular, MB-pol has been used by us and by the broader community to model water from small gas-phase clusters to the liquid phase and ice, consistently reproducing a wide range of experimental observables. These developments and validations have been reviewed in several articles [Chem. Rev. 116, 7501 (2016); Acc. Chem. Res. 49, 1844 (2016); J. Chem. Phys. 145, 194504 (2016); J. Chem. Theory Comput. 20, 9269 (2024); Annu. Rev. Phys. Chem. 77, 321 (2026)].

Among current molecular models of water, whether based on force fields or based on density functional theory (DFT), MB-pol is distinguished by its demonstrated ability to accurately reproduce structural, thermodynamic, dynamical, and spectroscopic properties of water from small clusters to liquid water and ice (see reviews listed above). As notable examples, MB-pol is currently the only model that accurately reproduces the phase diagram of water (Figure R3) [Nat. Commun. 14, 3349 (2023)] and has been used to predict the location of the liquid–liquid critical point [Nat. Phys. 21, 480 (2025)], which has recently been confirmed by experimental measurements [Science 391, 1318 (2026); Science 391, 1387 (2026)].

Directly relevant to the present study, MB-pol accurately reproduces vapor–liquid coexistence and interfacial properties, including the surface tension [J. Chem. Phys. 154, 211103 (2021)], as well as the vibrational sum-frequency generation (vSFG) spectrum of the air/water interface and its temperature and polarization

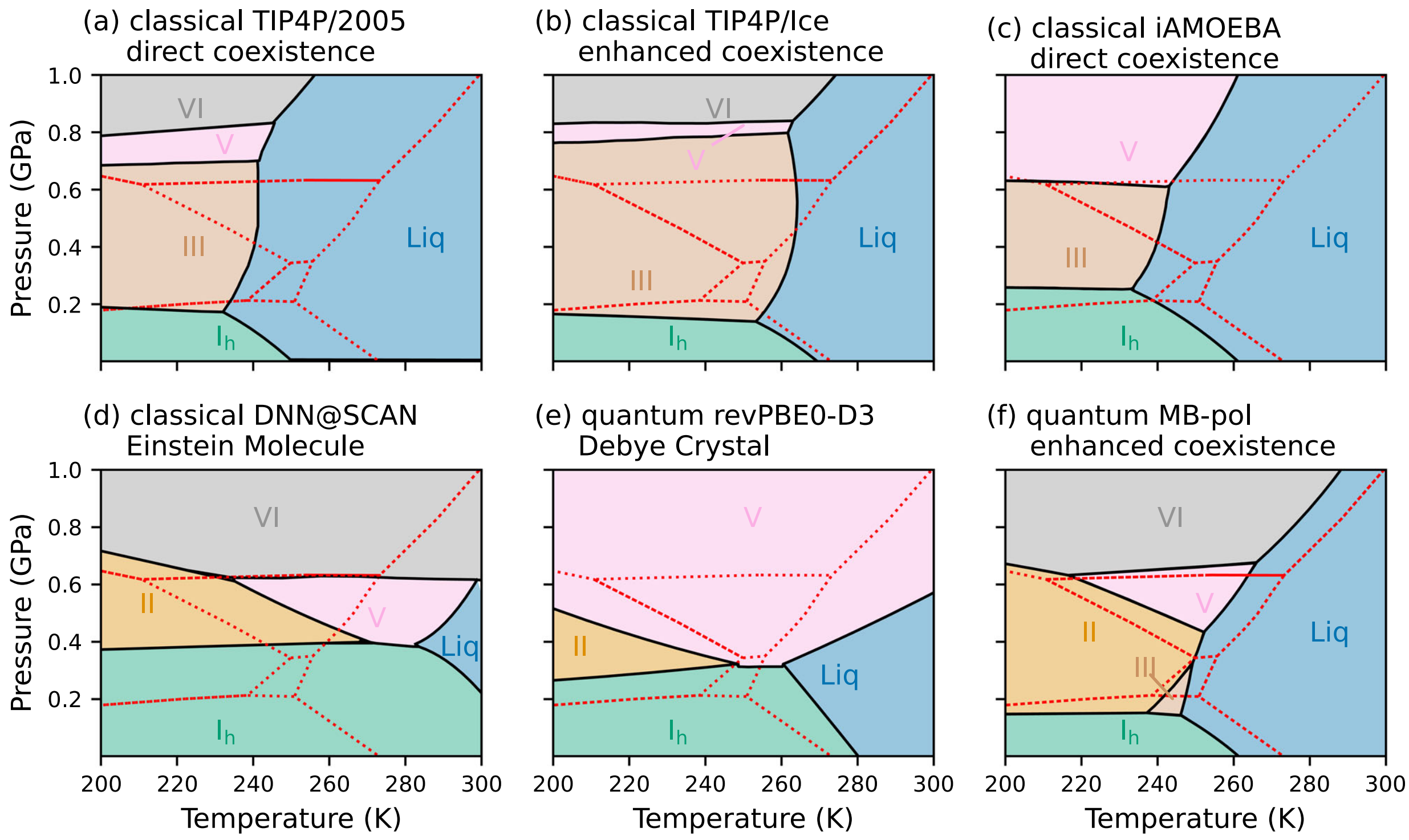

Figure R3: Phase diagrams of water calculated with different models: a) TIP4P/2005 (nonpolarizable force field), b) TIP4P/Ice (nonpolarizable force field), c) iAMOEBA (polarizable force field), d) DNN@SCAN (deep neural network trained data calculated with SCAN, a meta-GGA functional), e) revPBE0-D3 (hybrid density functional), and f) MB-pol. In each panel, the regions of stability for ice I_h, II, III, V, and VI and liquid water are shown as areas colored in green, orange, gray, brown, pink, and blue, respectively, and the experimental phase diagram is shown using dotted red lines. This comparison clearly shows that MB-pol is the only model that accurately predicts the phase behavior of water across a wide range of temperatures and pressures. Figure adapted from S.L. Bore and F. Paesani, *Nat. Commun.* 14, 3349 (2023); licensed under a Creative Commons Attribution (CC BY) license.

dependence [J. Am. Chem. Soc. 138, 3912 (2016); J. Phys. Chem. B 122, 4356 (2018); J. Phys. Chem. Lett. 9, 6744 (2018); Phys. Rev. Lett. 121, 246101 (2018)]. Recent work further showed that MB-pol accurately captures polarization-resolved signatures of free O–H groups and links them to orientation-dependent local electric fields, providing a realistic description of interfacial heterogeneity [J. Phys. Chem. Lett. 17, 3013 (2026)].

Finally, to further illustrate the accuracy and uniqueness of MB-pol, it should be noted that recent independent studies assessing DFT-based models of water have used MB-pol as a benchmark reference and found that it consistently outperforms existing DFT-based descriptions [https://doi.org/10.48550/arXiv.2512.23939; https://doi.org/10.48550/arXiv.2512.23940; https://doi.org/10.26434/chemrxiv.15000644/v1].

Regarding the MB-nrg models for halides in water, the original studies demonstrated that these models accurately reproduce the ion–water dimer potential energy surfaces at the CCSD(T) level [J. Chem. Theory Comput. 12, 2698 (2016); J. Chem. Phys. 147, 161715 (2017)]. In particular, the MB-nrg models were specifically shown to capture the highly directional, partially covalent F^-–H_2O interaction, as well as the softer and more diffuse interactions of the heavier halides [J. Chem. Theory Comput. 12, 2698 (2016)].

Systematic comparisons further demonstrated that MB-nrg accurately captures the balance of electrostatics, polarization, charge penetration, and charge transfer across different halide ions and hydration motifs [J. Comp. Theory. Comput. 15, 2983 (2019)]. In particular, a many-body analysis of halide–water dimers and trimers showed that MB-nrg quantitatively reproduces individual 2-body and 3-body CCSD(T) reference energies for $X^-(H_2O)_n$ (n = 1–4; X^- = F^-, Cl^-, Br^-, I^-), whereas both polarizable force fields and common DFT models exhibit significant deviations in the short-range regime where intermolecular orbital overlap becomes important [J. Comp. Theory. Comput. 15, 2983 (2019); Adv. Phys. X 4, 1631212 (2019)]. This is precisely the regime in which charge penetration and charge transfer become essential, and it is therefore directly relevant to the present analysis of interfacial ion hydration.

The MB-nrg models for halide ions were subsequently validated across a wide range of environments, from gas-phase clusters to aqueous solution, demonstrating that their transferability is not assumed but established *a posteriori*. In gas-phase ion–water clusters, full-dimensional quantum calculations of the vibrational spectra of $X^-(H_2O)$ and $X^-(D_2O)$ (X^- = F^-, Cl^-, Br^-, I^-) showed excellent agreement with experiment, including the correct OH-stretch redshifts and isotopic trends [J. Chem. Phys. 148, 102321 (2018)]. For halide–dihydrate complexes, ring-polymer instanton (RPI) and path-integral molecular dynamics (PIMD) simulations revealed ion-specific tunneling pathways and hydrogen-bond rearrangements that depend sensitively on isotopic substitution and temperature [Nat. Chem. 11, 367 (2019); J. Phys. Chem. Lett. 10, 2823 (2019)]. For larger $X^-(H_2O)_n$ clusters ($n = 3-6$), replica-exchange path-integral molecular dynamics (RE-PIMD) simulations further showed that the MB-nrg models accurately reproduce the structures, energetics, and hydrogen-bonding patterns across the halide series [J. Phys. Chem. A 123, 2843 (2019)].

In solution, classical MD and quantum PIMD simulations with MB-nrg models accurately reproduce the EXAFS spectra and hydration structure of Cl^-, Br^-, and I^-, in quantitative agreement with experiment [J. Chem. Phys. 155, 064502 (2021); J. Phys. Chem. B. 126, 8266 (2022)]. Spectroscopic signatures of halide hydration in bulk water were analyzed using both classical and quantum molecular dynamics: strong hydrogen bonding in fluoride hydration was shown to produce large OH-stretch redshifts and bifurcated librational bands [J. Phys. Chem. B. 129, 7301 (2025)], whereas weaker and more diffuse perturbations were found for Cl^-, Br^-, and I^- [J. Phys. Chem. B. 129, 6584 (2025)]. These results reinforce that the MB-nrg models consistently capture both energetic and spectroscopic manifestations of ion-specific hydration across environments. More recently, the MB-nrg models have also been shown to accurately predict hydration free energies for all halide ions, further strengthening the case that they provide a transferable description of halide hydration from isolated clusters to the condensed phase [J. Chem. Theory Comput. 22, 4688 (2026)].

In summary, the MB-pol and MB-nrg models are considered by the community as the most realistic and extensively validated molecular models currently available for water and halide–water systems. Their demonstrated accuracy across gas-phase, bulk, and interfacial properties provides a strong basis for applying them to investigating halide ion propensity for the air/water interface.

Response to Reviewer 2

The manuscript entitled "Many-Body Interactions Resolve Halide Distributions at the Air/Water Interface" presents a systematic study of the adsorption behavior of halide ions, specifically Cl-, Br-, and I-, at the air–water interface. In this work, the authors incorporate higher-order many-body interaction terms into the potential energy and force calculations, and demonstrate that these higher-order contributions qualitatively alter the adsorption mechanisms predicted by classical force fields and earlier versions of MB potentials that neglected such terms. In particular, the revised physical picture appears to affect Cl- and Br- more significantly. Overall, the manuscript is reasonably well executed, and the conclusions are supported by generally sound arguments. That said, I do not believe the present work reaches the level of breakthrough and novelty typically expected for publication in Nature Communications. In my opinion, the study would be more suitable for a specialized journal such as Scientific Reports. My comments are listed below:

We thank the Reviewer for their detailed assessment of our manuscript and for recognizing that the study is well executed and that the conclusions are supported by sound arguments. We respectfully disagree, however, with the view that the present work lacks the novelty and significance appropriate for publication in *Nature Communications*.

The central advance of this work is not simply that higher-order many-body terms modify previously reported adsorption profiles. Rather, the novelty lies in establishing a quantitatively resolved microscopic picture of halide surface propensity at the air/water interface using a chemically accurate, systematically controlled many-body hierarchy. This hierarchy resolves a long-standing ambiguity in the field by showing how the limiting pictures provided by classical image-charge theory and empirical polarizable force fields emerge from different approximations to the interaction Hamiltonian and why neither limit alone provides the correct molecular description.

Equally important, the present analysis goes beyond reporting potentials of mean force. It provides a quantitative decomposition of the interfacial thermodynamics into ion–water desolvation, short-range quantum-mechanical many-body effects, water cavity/reorganization, and entropic contributions. To our knowledge, such a decomposition has not previously been reported for the full halide series at the air–water interface. It is also not accessible from conventional empirical force fields, which fold different physical effects into effective parameters, or from DFT-based simulations, which cannot isolate the relevant physical ingredients in the same controlled manner.

We therefore believe that the present manuscript provides an important advance for the fields of aqueous interfaces, ion specificity, and molecular simulation. It explains why Cl^- and Br^- lose the interfacial minima predicted by polarizable models, identifies the finely balanced many-body mechanism that leaves only I^- with weak surface activity, and shows that halide surface propensity is not governed by ionic size or polarizability alone.

1. The work reads primarily as an application of an incremental extension of the existing MB-potential framework based on semi-empirical many-body methods. In the present case, the authors demonstrate that higher-order many-body terms are important for describing the adsorption of halide ions at the air–water interface. As such, the work appears more as an application of an incremental improvement of the MB-pol methodology, rather than a fundamentally new conceptual advance.

We thank the Reviewer for this comment. We respectfully clarify, however, that the MB-nrg potentials used in this work are not semi-empirical many-body models. Rather, they are data-driven many-body potential energy functions in which the short-range 2-body and 3-body ion–water terms are machine learned representations that are combined with physics-based permanent electrostatics, dispersion, and many-body polarization to provide a size-extensive, CCSD(T)-level representation of the underlying many-body potential energy surface from gas-phase clusters to condensed-phase environments. Importantly, the MB-nrg potentials were not parameterized to reproduce interfacial adsorption free energies, vibrational spectra, hydration free energies, or other experimentally accessible observables or thermodynamic quantities. These properties are instead out-of-sample predictions that consistently emerge from the same CCSD(T)-anchored MB-nrg many-body representation of the underlying potential energy surface. Specifically, their transferability has been established in the literature through comparisons with gas-phase cluster energetics and spectra [J. Chem. Theory Comput. 12, 2698 (2016); J. Chem. Phys. 148, 102321 (2018); J. Comp. Theory. Comput. 15, 2983 (2019); Adv. Phys. X 4, 1631212 (2019); Nat. Chem. 11, 367 (2019); J. Phys. Chem. Lett. 10, 2823 (2019); J. Phys. Chem. A 123, 2843 (2019)], hydration structure and EXAFS spectra [J. Chem. Phys. 155, 064502 (2021); J. Phys. Chem. B. 126, 8266 (2022)], vibrational spectra [J. Phys. Chem. B. 129, 7301 (2025); J. Phys. Chem. B. 129, 6584 (2025)], and hydration free energies [J. Chem. Theory Comput. 22, 4688 (2026)]. The MB-pol and MB-nrg potentials are considered by the community as the most realistic and extensively validated molecular models currently available for water and halide–water systems. Their demonstrated accuracy across gas-phase, bulk, and interfacial properties provides a strong basis for applying them to investigating halide ion propensity for the air/water interface.

We also respectfully disagree that the present work is primarily an incremental application of an improved MB-pol/MB-nrg methodology. As discussed above, the methodological framework has already been developed and validated in previous work; the purpose of the present manuscript is different. Here, we use the MB-nrg hierarchy as a controlled physical probe to resolve the molecular origin of halide surface propensity at the air/water interface. This hierarchy allows us to selectively examine pairwise ion–water interactions, classical many-body polarization, and explicit short-range quantum-mechanical many-body effects within a single internally consistent Hamiltonian. This controlled quantitative analysis is uniquely enabled by the MB-nrg many-body formalism and is not available in the same internally consistent manner from conven-

tional force fields or from DFT-based models.

The conceptual advance is therefore not the observation that higher-order many-body terms are numerically important, but the physical conclusion that the traditional interpretation of halide adsorption in terms of ionic size and polarizability alone is incomplete. Classical many-body polarization by itself over-stabilizes the heavier halides, producing the strong I^- adsorption and non-negligible Br^- and Cl^- adsorption typical of empirical polarizable force fields. In contrast, the full many-body MB-nrg description suppresses the Br^- and Cl^- minima and reduces iodide adsorption to a weak, near-thermal effect.

This result changes the physical picture of halide adsorption at the air/water interface. The interface is not described correctly either by generic image-charge exclusion for all ions or by polarizability-driven adsorption of the heavier halides. Instead, the full many-body description yields an intermediate regime in which F^-, Cl^-, and Br^- remain thermodynamically bulk-favored, while only I^- retains weak surface activity.

2. From the perspective of materials and chemical physics, the analyses and discussions are not sufficiently deep or broad. Most of the presented results and analyses are restricted to adsorption-related properties, such as free-energy profiles and configurational energetics at the interface. A broader analysis of the underlying microscopic physics or implications for interfacial chemistry would strengthen the impact of the work. More importantly, the simulation results are not supported by calculations of experimentally measurable spectroscopic observables, such as sum-frequency generation spectroscopy (SFG or vSFG). Simulations of such spectra would allow for more direct comparison with experiments and would substantially strengthen the credibility and significance of the conclusions.

We thank the Reviewer for this helpful suggestion. We agree that connecting the free-energy and energetic analyses to an experimentally accessible interfacial observable provides a broader and more complete physical picture. At the same time, we emphasize that the primary objective of the present work is to address the fundamental single-ion question at the heart of the classical theories of Wagner and Onsager–Samaras, and of much of the experimental and simulation literature on halides at aqueous interfaces over the past two decades: does an individual halide ion have an intrinsic thermodynamic propensity for the air–water interface, and what molecular mechanisms and thermodynamic balance determine that propensity? Accordingly, our simulations deliberately target the dilute single-ion limit, in which the halide response can be isolated from counterion effects, finite-concentration interfacial restructuring, ion–ion correlations, and ion pairing.

Nevertheless, we agree with the Reviewer that a spectroscopic analysis can provide an important complementary test of the physical picture developed from the PMFs. In response, we have calculated the heterodyne-detected vibrational sum-frequency generation (HD-vSFG) spectra in the XXZ polarization combination for the air/water interface containing a single halide ion using all four ion–water potentials considered in the manuscript: TTM-nrg, (2B)-MB-nrg, (2B+NB)-MB-nrg, and (2B+3B+NB)-MB-nrg. The resulting spectra are shown in Figure R4 and have been added to the Supporting Information as Supplementary Note 8.

These calculated spectra provide a direct spectroscopic signature of the same many-body hierarchy observed in the Helmholtz free-energy profiles. For neat water, the positive feature near 3700 cm^{-1} is associated with free O–H groups, whereas the negative band in the 3200–3600 cm^{-1} region is associated with hydrogen-bonded O–H groups. When a halide ion resides near the interface, the local hydration environment becomes asymmetric: water molecules remain predominantly on the liquid side of the ion, with O–H groups oriented toward both the halide ion and the vapor phase, without a compensating layer of oppositely oriented water molecules on the air side. This orientational imbalance decreases the free O–H response and produces, or enhances, a positive contribution in the hydrogen-bonded O–H stretching region. Thus, while these spectra are not meant to reproduce finite-concentration electrolyte measurements quantitatively, they provide a useful qualitative vibrational marker of ion population near the interface.

The computed spectra closely follow the free-energy trends in Figure 1 of the main text. With TTM-nrg, which predicts pronounced interfacial stabilization for I^- and smaller minima for Br^- and Cl^-, the spectra show a positive hydrogen-bonded O–H feature near 3400 cm^{-1} together with a reduced free O–H peak for I^-, Br^-, and Cl^-. By contrast, with the full (2B+3B+NB)-MB-nrg potential, an appreciable surface-specific response is observed only for I^-, consistent with the shallow iodide minimum in the corresponding PMF. The spectra obtained with the purely pairwise-additive (2B)-MB-nrg potential do not show a positive hydrogen-bonded O–H feature for any halide ion, again consistent with the repulsive PMFs. Finally, the

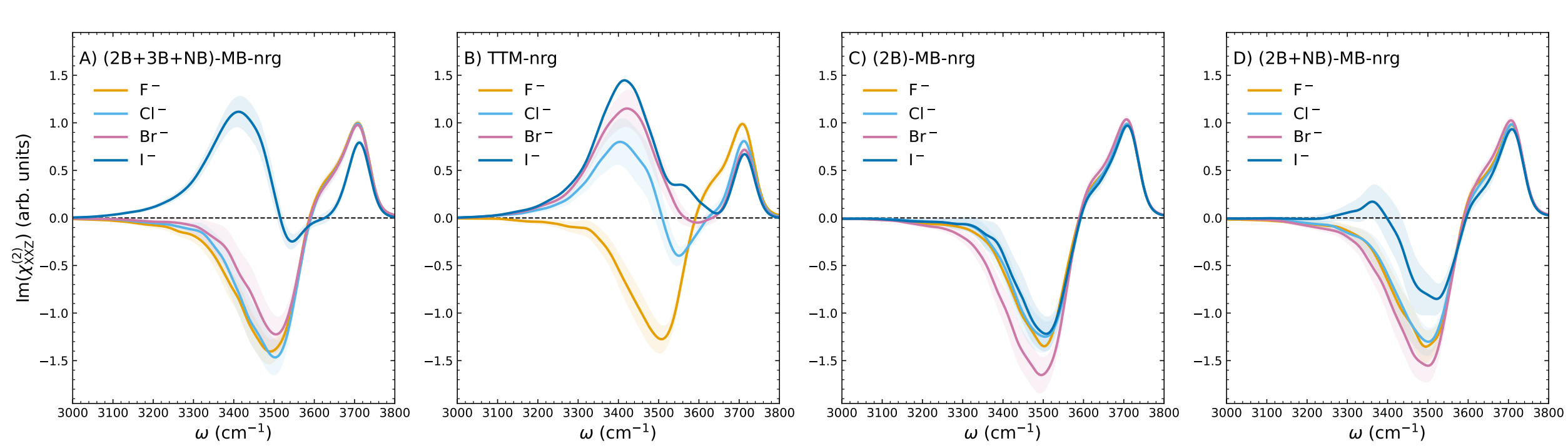

Figure R4: HD-vSFG spectra in the XXZ polarization combination calculated for the single-halide air/water interface using the same panel order as the PMFs in Figure 1 of the manuscript: (A) (2B+3B+NB)-MB-nrg, (B) TTM-nrg, (C) (2B)-MB-nrg, and (D) (2B+NB)-MB-nrg.

spectra computed with (2B+NB)-MB-nrg show only a weak positive feature near 3400 cm^{-1} for I^- within statistical uncertainty, in line with the shallow local minima in the corresponding free-energy profiles. These results demonstrate that the hierarchy of ion surface propensity inferred from the PMFs is also reflected in the interfacial O–H stretch vibrational response.

We also note that the full (2B+3B+NB)-MB-nrg spectra do not support the extreme-propensity interpretation proposed in Ref. 14, in which all halides except F^- were inferred to exhibit strong surface propensity. Instead, they are qualitatively consistent with the recent HD-vSFG measurements of finite-concentration electrolyte solutions reported in Ref. 15, in the sense that they do not show spectral signatures of strong halide accumulation in the topmost interfacial layer. Our interpretation, however, is necessarily different because the present simulations were deliberately designed to isolate the intrinsic single-ion limit. Thus, the spectra reported here should be viewed as a spectroscopic consistency check of the PMF-derived single-ion propensities, rather than as a direct test of finite-concentration stratification. Finite-concentration solutions include counterions, ion–ion correlations, ion pairing or association, and concentration-dependent water restructuring, all of which introduce additional many-body effects and can consequently modify the vSFG response beyond the scope of the present study. Indeed, our preliminary simulations of finite-concentration electrolyte solutions suggest that these systems involve additional physics that deserves a dedicated investigation in its own right. Future work with MB-nrg potentials will therefore address finite-concentration electrolyte solutions both in bulk water and at the air/water interface, including explicit counterions and concentration-dependent interfacial structure.

Revision: In the revised manuscript, we have added a brief discussion of the computed HD-vSFG spectra to the main text. The spectra are included in the Supporting Information as Supplementary Figure S17, with methodological details and additional discussion provided in Supplementary Note 8:

> To provide a complementary spectroscopic signature of the many-body hierarchy observed in the Helmholtz free-energy profiles, we calculated the imaginary component of the resonant heterodyne-detected vSFG (HD-vSFG) response, $\mathrm{Im}\,\chi^{(2)}_{XXZ}(\omega)$, for the same single-halide air/water interfaces and ion–water potentials used in Figure 1. The calculated HD-vSFG spectra, shown in Supplementary Figure S17, are not intended for direct quantitative comparison with finite-concentration vSFG measurements of electrolyte solutions[11,12,14,15] because the present simulations probe the dilute single-ion limit and therefore exclude counterions, ion–ion correlations, ion pairing, and concentration-dependent interfacial restructuring. Nevertheless, the full (2B+3B+NB)-MB-nrg spectra do not support the extreme-propensity interpretation proposed in Ref. 14, according to which the heavier halides exhibit strong surface propensity. Instead, they are qualitatively consistent with the more recent HD-vSFG measurements of electrolyte solutions reported in Ref. 15, in the sense that they do not show spectral signatures of strong halide accumulation in the topmost interfacial layer, but only an iodide-specific response. The interpretation, however, is different: in the present single-ion limit, the spectra reflect the intrinsic thermodynamic propensity of each halide for the air/water interface, whereas finite-concentration experiments may include additional effects from counterions, ion–ion correlations, and concentration-dependent stratification.
>
> The calculated spectra closely follow the free-energy hierarchy. Consistent with Figure 1, TTM-nrg produces a positive hydrogen-bonded O–H feature near 3400 cm^{-1}, accompanied by a decrease in the free O–H peak near 3700 cm^{-1}, for I^-, Br^-, and Cl^-, reflecting the enhanced surface propensity predicted by the polarizable baseline. In contrast, the full (2B+3B+NB)-MB-nrg potential yields an appreciable surface-specific hydrogen-bonded O–H response only for I^-. The (2B)-MB-nrg spectra show no positive hydrogen-bonded O–H feature for any halide ion, whereas the (2B+NB)-MB-nrg spectra show only a weak positive feature near 3400 cm^{-1} for I^- within statistical uncertainty. Thus, the interfacial hydrogen-bonded O–H stretch response mirrors the same many-body hierarchy obtained from the PMFs, providing an independent spectroscopic consistency check of the predicted halide surface propensities.

3. One minor issue: in the terminology "2B+3B+NB-nrg", the meaning of the term "NB" does not appear to be clearly defined in the main text. The authors should clarify this notation upon first introduction.

We thank the Reviewer for pointing this out. We agree that the notation should be defined more clearly upon first use. In the MB-nrg notation used here and in previous studies with MB-nrg potentials, "2B" denotes explicit 2-body ion–water terms, "3B" denotes the explicit short-range 3-body ion–water–water term, and "NB" denotes the classical N-body, or many-body, polarization contribution represented through the extended Thole scheme. We have revised the main text accordingly when the model hierarchy is first introduced.

Revision: In the revised manuscript, we have defined the 2B/3B/NB notation when the MB-nrg hierarchy is first introduced in the Results and Discussion section. The revised text now reads:

> The MB-nrg potentials refine ion–water interactions using the established 2B/3B/NB notation: "2B" denotes explicit 2-body ion–water terms, "3B" denotes the explicit short-range 3-body ion–water–water term, and "NB" denotes the classical N-body, or many-body, polarization contribution represented through the extended Thole scheme. Accordingly, (i) (2B)-MB-nrg includes a machine-learned short-range 2-body ion–water term, together with permanent electrostatics and pairwise dispersion of the same functional forms used in TTM-nrg; (ii) (2B+NB)-MB-nrg augments (2B)-MB-nrg with the NB many-body polarization term; and (iii) (2B+3B+NB)-MB-nrg further adds the explicit machine-learned 3-body ion–water–water term that captures short-range nonadditive quantum effects.

Response to Reviewer 3

The authors use a data-driven many-body simulation potential to determine the interfacial thermodynamics of halide ions at the air-water interface. They find a weak adsorption of iodide, which by energy decomposition they show to be due to water cavity/reorganization contribution that compensates ion–water desolvation. The paper is nicely written, ion-specific effects at interfaces are important and still pose a number of questions, the numerical framework developed and used by the authors is sound, I therefore recommend publication of the paper and only have a few comments on the presentation.

We thank the Reviewer for their thoughtful assessment of our manuscript and for supporting its publication in *Nature Communications*.

We have addressed the comments below by clarifying the scope of the single-ion simulations, refining the discussion of non-polarizable force fields and ionic polarizability, and adding comments on the relationship between the calculated single-ion PMFs and finite-concentration surface-tension measurements.

1. The authors write "In general, molecular dynamics (MD) simulations with non-polarizable force fields yield no surface affinity ..." which is not really true. In the rather old review Ref. 5 non-polarizable force fields were shown to give rise to surface adsorption, in the more recent paper "Orientation-Induced Adsorption of Hydrated Protons at the Air–Water Interface", Mamatkulov et al, Angew. Chem. Int. Ed. 2017, 56, 15846 –15851, MD simulations with non-polarizable force fields for hydronium, hydroxide, sodium, chloride, that were optimized with respect to bulk ionic properties (solvation free energies and activities), were shown to yield pronounced adsorption for hydronium and to give rise to a surface tension for HCl that in fact decreases with increasing electrolyte concentration in quantitative agreement with experimental data.

We thank the Reviewer for pointing this out. We agree that the original wording was too broad and could be read as applying to all non-polarizable force fields and to all ions. Our intended statement referred specifically to the behavior of standard fixed-charge, non-polarizable models for simple alkali-halide salts, which generally predict little or no halide surface affinity compared with empirical polarizable force fields. We have revised the manuscript to make this point more precise.

The work cited by the Reviewer is valuable and complementary, but it addresses a different physical question. Mamatkulov *et al.* studied finite-concentration acid/base/salt solutions using thermodynamically optimized non-polarizable force fields for H_3O^+, OH^-, Na^+, and Cl^- in SPC/E water. In that study, the pronounced interfacial adsorption was specific to H_3O^+ and was attributed to the orientational coupling of the hydronium dipole to the interfacial electric field; OH^-, Cl^-, and Na^+ were instead primarily repelled from the interface. This distinction is important because H_3O^+ is a molecular ion with a permanent dipole and orientational degrees of freedom, whereas the present work focuses on the intrinsic surface propensity of monatomic halide anions. Indeed, the Cl^- result in that study is consistent with the standard fixed-charge picture for monatomic halides, in which non-polarizable force fields generally predict little or no halide surface affinity.

There is also an important distinction in modeling philosophy. The force fields used in the cited work were optimized to reproduce selected bulk thermodynamic properties, including solvation free energies and activities of $H_3O^+Cl^-$ and Na^+OH^- solutions. Such thermodynamically optimized non-polarizable models can be highly useful for reproducing macroscopic observables such as surface tensions. However, because missing electronic polarization and many-body effects are necessarily absorbed into effective fixed-charge and Lennard-Jones parameters, this type of model does not provide a unique molecular decomposition of the physical contributions that determine ion adsorption. By contrast, the goal of the present study is to use the MB-nrg hierarchy as a controlled many-body probe of the intrinsic surface propensity of halide ions, separating pairwise ion–water interactions, classical many-body polarization, explicit short-range quantum-mechanical many-body effects, water cavity/reorganization, and entropy within a single internally consistent Hamiltonian.

We therefore agree with the Reviewer that non-polarizable force fields can, depending on their parametrization and on the ion considered, produce interfacial adsorption. We have revised the Introduction accordingly and have included the cited paper in the relevant discussion because its Cl^- result is consistent with the general trend observed with conventional non-polarizable models for monatomic halide ions (i.e., little or no halide surface affinity).

Revision: In the revised manuscript, we have modified the relevant sentence in the Introduction to avoid implying that all non-polarizable force fields predict no ion adsorption. The revised text now reads:

> Molecular dynamics (MD) simulations have yielded model-dependent predictions for halides at the air/water interface. For simple alkali-halide salts, conventional fixed-charge, non-polarizable force fields generally predict little or no halide surface affinity, whereas empirical polarizable models often stabilize the larger, softer anions in the interfacial region.[2,16–22]

2. Related to this, the rather pronounced decrease of the HCl surface tension of about 0.25 mJ/m^2 per mol/l of added HCl is caused by a minimum in the PMF of hydronium of about 1 k_BT. As the authors correctly note, the surface tension of e.g. NaI solutions increases with added electrolyte concentration but with a slope that is reduced compared to NaCl solutions: The increase of NaCl is about 1.5 mJ/m^2 per mol/l while the increase of NaI is about 1.0 mJ/m^2 per mol/l (from Jarvis Scheimann 1968). The depth of the free energy profile obtained for iodide by the authors in Fig. 2a is about 0.25 kcal/mol which converts to about 1 kJ/mol or 0.5 k_BT. It seems that the minimum is too deep compared to experimental surface tension data

for iodide electrolytes. Could the authors estimate the surface tension from the expected interfacial excess and compare with experimental data or at least comment on this?

We thank the Reviewer for raising this important point. We agree that surface-tension measurements provide an essential macroscopic benchmark for electrolyte interfaces. At the same time, we respectfully note that the present single-ion PMF cannot be converted directly into the surface-tension increment of a finite-concentration NaI solution. The surface tension of an electrolyte solution is a thermodynamic property of an electroneutral finite-concentration system and, through the Gibbs adsorption equation, depends on the surface excess of the neutral electrolyte under the appropriate thermodynamic constraints. It therefore depends on both cation and anion distributions, counterion effects, ion–ion correlations, possible ion pairing or association, and concentration-dependent interfacial restructuring. These effects are deliberately excluded from the present simulations, which isolate the intrinsic propensity of a single halide ion at the air/water interface in the absence of counterion-specific contributions.

For this reason, the iodide minimum in our PMF should not be interpreted as implying that NaI should reduce the surface tension of water. Rather, it indicates that, relative to less surface-active halides such as Cl^-, I^- has a weak intrinsic tendency to sample the interfacial region. This is qualitatively consistent with the experimental observation noted by the Reviewer: NaI solutions still increase the surface tension with concentration, but with a smaller slope than NaCl solutions. In other words, weak iodide surface activity can reduce the magnitude of the electrolyte-induced surface-tension increase relative to NaCl without reversing its sign.

A simple order-of-magnitude estimate also shows why a shallow single-ion PMF minimum is not necessarily inconsistent with the surface-tension slopes. In an ideal dilute single-particle picture, an interfacial stabilization of order 0.5 $k_B T$ extending over a molecular length scale of approximately 1 Å would correspond to an adsorption length of order one molecular diameter or less. Through the Gibbs adsorption relation, such a molecular-scale excess would modify the surface-tension slope by only a few tenths of mJ/m^2 per mol/L, i.e., on the same scale as the difference between the NaCl and NaI slopes quoted by the Reviewer. This estimate is only illustrative because it neglects the Na^+ contribution and electroneutrality constraints, but it supports the interpretation that the iodide PMF minimum represents weak intrinsic surface activity rather than strong electrolyte adsorption.

We have added a short comment to the manuscript clarifying that the present single-ion PMFs should not be converted directly into finite-concentration surface-tension increments, as the finite-concentration electrolyte simulations with explicit counterions will be the subject of future work.

Revision: In the revised manuscript, we have added the following clarification:

> Because the present calculations probe the dilute single-ion limit, they are not directly comparable to finite-concentration measurements and simulations, nor can the resulting PMFs be directly converted into surface-tension increments for finite-concentration electrolyte solutions. Through the Gibbs adsorption equation, electrolyte surface tensions depend on the surface excess of the electroneutral salt and therefore require both cation and anion distributions, ion–ion correlations, possible ion pairing, and concentration-dependent interfacial restructuring. The shallow iodide minimum found here should therefore be interpreted as an intrinsic single-ion propensity that can reduce the surface-tension increment of alkali-metal iodide solutions relative to corresponding salts of less surface-active halides, rather than as evidence for a negative surface-tension increment of alkali-metal iodide solutions. Consistent with this interpretation, NaI solutions experimentally increase the surface tension with concentration, but less strongly than NaCl solutions.[74] Within this single-ion scope, the present results do not indicate either the extreme surface propensity suggested by early polarizable-model simulations[2] and inferred from some vSFG measurements,[14] or the ion-depleted topmost interfacial layer inferred in more recent stratification analyses.[15] Instead, they are most consistent with modest iodide surface activity, while Br^- and Cl^- remain thermodynamically bulk-favored. In this sense, the present findings remain broadly consistent with theoretical descriptions that incorporate ionic polarizability and cavitation[28,29] and are qualitatively compatible with photoelectron evidence for near-surface enrichment of the heavier halides.[13]

3. The authors in their discussion and interpretation repeat an old argument according to which the polarizability of ions furnishes a natural mechanism for interfacial propensity. I do not see how such a mechanism is obtained from basic dielectric theory: Firstly, the electronic polarizability of iodide is indeed larger than that of chloride, which is simply a size effect, since the polarizability of atoms scales with their volume. However, the low-frequency excess polarizability of an ion in an aqueous solution is negative, since an ion replaces water that has a much larger low-frequency polarizability due to its permanent dipole moment. Secondly, the polarizability of an object translates to an interfacial propensity only for a charged interface, but not to a neutral interface. The authors correctly point out that the interaction of an ion with an interface is dictated by solvation effects, this is supported by semi-quantitative modeling of ion-specific interfacial interaction using solvation theory in Ref. 5. I would adapt the wording slightly in the introduction.

We thank the Reviewer for this insightful comment. We agree with the Reviewer that ionic polarizability alone should not be presented as a complete or generic mechanism for adsorption at a neutral air/water interface. Our intention was not to claim that electronic polarizability by itself drives halide surface propensity. Rather, we used the term "polarizable models" to refer to a broad class of empirical force fields and continuum theories in which ionic polarizability is coupled to other physical ingredients, including hydration, cavitation, dispersion, excluded-volume effects, and interfacial solvation. This distinction is important

because continuum theories such as those of Levin and co-workers do not invoke polarizability in isolation, but treat it together with solvation/cavitation effects to describe ion distributions and surface tensions at electrolyte interfaces.

Indeed, one of the central conclusions of the present work is that polarizability alone is insufficient. In our model hierarchy, adding classical many-body polarization to conventional pairwise repulsion and dispersion, as in TTM-nrg, over-stabilizes the heavier halides, yielding pronounced interfacial minima for I^- and smaller minima for Br^- and Cl^-. Only when explicit short-range quantum-mechanical many-body ion–water interactions are included does the intermediate regime emerge in which Cl^- and Br^- remain thermodynamically bulk-favored and only I^- retains weak surface activity. Thus, the present results are consistent with the Reviewer's broader point that halide surface propensity is governed by interfacial solvation and water reorganization, not by ionic polarizability alone.

We have revised the Introduction and the discussion of continuum models to avoid language that could be read as assigning adsorption to polarizability alone. The revised wording now emphasizes that prior polarizable models and continuum theories invoke polarizability together with cavitation and interfacial solvation effects, whereas the present MB-nrg results show that the final surface propensity is determined by a many-body balance among ion–water desolvation, water cavity/reorganization, short-range quantum-mechanical interactions, classical polarization, and entropy.

Revision: In the revised manuscript, we have modified the relevant wording in the Introduction as follows:

> Several theoretical models have also been proposed, including dielectric-continuum descriptions of polarizable anions[28] and subsequent extensions that combine ionic polarizability with cavitation and interfacial solvation effects,[29] as well as fluctuation-based descriptions that emphasize interfacial density and electric-field fluctuations,[30,31] offering complementary perspectives on how molecular-scale structure informs macroscopic observables.

We have also revised the discussion of continuum theories:

> The model hierarchy also clarifies connections to classical and continuum theories. The purely pairwise-additive (2B)-MB-nrg description reproduces the generic exclusion predicted by Wagner and by Onsager–Samaras at a dielectric discontinuity. By contrast, combining many-body polarization with conventional pairwise-additive repulsion and dispersion, as in TTM-nrg, yields a pronounced interfacial minimum for I^- and smaller minima for Br^- and Cl^-, qualitatively consistent with the original dielectric-continuum treatment of polarizable anions, in which charge redistribution within a polarizable ion lowers the electrostatic penalty for approaching the dielectric interface.[28] When many-body polarization is integrated with an explicit 2-body ion–water term, as in (2B+NB)-MB-nrg, the resulting profiles are everywhere non-negative, with only shallow near-surface local minima, in qualitative agreement with the extended continuum theory in which ionic polarizability is combined with cavitation and interfacial solvation effects.[29] In this sense, the full (2B+3B+NB)-MB-nrg potential provides a microscopic, many-body realization of the cavity/reorganization physics that enters continuum descriptions phenomenologically: the explicit short-range 2-body and 3-body ion–water terms do not constitute a cavitation term per se, but instead determine how the surrounding water environment reorganizes and thereby modulate the effective cavity/reorganization contribution. The fact that (2B+NB)-MB-nrg already resembles the extended continuum picture indicates that accurate short-range 2-body ion–water physics captures much of the same net effect represented phenomenologically as cavitation, while the further changes induced by explicit 3-body interactions show that this cavity/reorganization physics is itself modulated by higher-order many-body effects absent from continuum models. Importantly, the disappearance of interfacial minima for Cl^- and Br^- upon inclusion of explicit 3-body interactions highlights a key limitation of continuum approaches, namely the absence of quantum-mechanical many-body effects that ultimately modulate halide surface propensity.

Response to Reviewer 1

The authors have replied to my comments and I believe the current version of the manuscript is suitable for publication in Nature Communications.

We thank the Reviewer for the careful evaluation of our manuscript and for supporting publication of the revised version in Nature Communications. We appreciate the Reviewer's constructive comments, which helped us improve the clarity, reproducibility, and presentation of the work.

Response to Reviewer 2

In the response letter and the revised manuscript, I believe the authors have only partially addressed my previous comments and concerns. While I appreciate the additional analyses and discussions, I remain unconvinced that the manuscript meets the standard for publication in Nature Communications. In my opinion, the work does not provide a sufficient level of conceptual novelty, mechanistic insight, or experimental validation to justify publication in this journal. My detailed comments are as follows:

We thank the Reviewer for the continued evaluation of our manuscript. We respectfully disagree with the conclusion that the revised work lacks the conceptual novelty, validation, or mechanistic depth appropriate for *Nature Communications*. The Reviewer's remaining comments do not identify a technical error in the simulations or a conclusion that is inconsistent with the reported data. Rather, they reflect differing judgments regarding the significance of the scientific question, whether a theoretical study must be accompanied by a directly corresponding experiment, and the form that a mechanistic explanation should take.

Our manuscript addresses each of these issues explicitly. It establishes a controlled connection among classical image-charge theory, empirical polarizable models, continuum descriptions incorporating polarizability and cavitation, and a chemically accurate molecular many-body representation. It quantitatively resolves the contributions governing the free energy across the complete halide series and provides an independent HD-vSFG spectroscopic signature of the predicted hierarchy. We respond to the specific points below.

1. With regard to the novelty of the work, the authors have not provided sufficiently convincing arguments in their response. In fact, they explicitly acknowledge that "the methodological framework has already been developed and validated in previous work." This statement itself suggests that the present manuscript primarily represents an application of an existing computational methodology to a new system. While such applications can certainly be scientifically valuable, the current study does not provide sufficiently compelling evidence that the resulting theoretical predictions constitute a significant conceptual advance or that the proposed adsorption mechanism has been experimentally validated.

We respectfully disagree that the prior development of the MB-nrg methodology diminishes the conceptual novelty of the present study. Scientific novelty resides not only in the development of new methods, but also in the new knowledge and mechanistic insight that established, rigorously validated methods make possible. Indeed, established theoretical frameworks such as molecular dynamics and density functional theory routinely enable conceptually novel discoveries when applied to unresolved scientific questions. In the present work, the validated MB-nrg framework is used as a controlled molecular probe to resolve a century-old question concerning intrinsic halide distributions at the air/water interface and to establish how classical image-charge exclusion, polarizable-model adsorption, and chemically accurate many-body behavior emerge from distinct approximations to the interaction Hamiltonian. The novelty of the present work is therefore not the development of MB-nrg itself, but the first systematic molecular resolution of these previously incompatible theoretical pictures across the complete halide series. The prior establishment of the computational framework does not diminish the novelty of the scientific discovery; rather, its extensive independent validation is what makes the resulting conceptual conclusion credible.

The question addressed by our study is fundamental: does an individual halide ion possess an intrinsic thermodynamic propensity for the air/water interface, and which interactions determine that propensity? This question originates in the foundational image-charge treatments of Wagner and Onsager–Samaras and has been revisited for more than a century through continuum theories, force field simulations, electronic structure calculations, and surface-sensitive measurements. The persistence of mutually incompatible pictures, including universal image-charge exclusion, strong polarizability-driven adsorption, extreme halide surface enhancement, and an ion-depleted topmost layer, demonstrates that the problem has remained unresolved. Indeed, experimental studies using similar techniques have reached completely opposite conclusions, inferring surface ion concentrations several times larger than the bulk in some cases and an ion-depleted outermost layer in other cases.

The advance enabled by the present many-body hierarchy is that it unifies these previously disconnected pictures within a single, internally consistent molecular framework. Specifically:

1. the pairwise limit reproduces classical image-charge exclusion;
2. conventional repulsion and dispersion combined with classical many-body polarization produce the pronounced adsorption of the heavier halides characteristic of empirical polarizable models;
3. introducing a chemically accurate short-range 2-body term reduces this adsorption to shallow local minima that remain above the bulk reference;
4. including explicit short-range 3-body quantum-mechanical interactions removes the Cl^- and Br^- minima and leaves only weak, near-thermal I^- surface activity.

This is not simply an application of an existing model to another system. It establishes, for the first time, how the principal theoretical descriptions of halide adsorption arise from specific approximations to the molecular interaction Hamiltonian and why none of the lower-order descriptions provides the complete physical picture. These results revise the conventional interpretation that halide surface propensity follows primarily from ionic size or softness and replace it with a quantitative many-body mechanism involving ion–water desolvation, short-range quantum interactions, classical polarization, water cavity/reorganization, and entropy.

We therefore respectfully maintain that the work constitutes a significant conceptual advance in aqueous interfacial chemistry, enabled by the prior development and extensive validation of the computational framework.

2. The manuscript does not provide direct experimental support for its central mechanistic conclusion. Although theoretical HD-vSFG spectra are presented in the revised manuscript, these spectra remain purely theoretical and are neither quantitatively compared with experimental measurements nor calculated under realistic experimental conditions. The simulations consider only a single halide ion at the air/water interface and neglect the presence of counter ions, finite electrolyte concentrations, ion pairing, and collective interfacial effects that are inevitably present in experimental systems. Consequently, the calculated spectra should be regarded as theoretical predictions rather than experimental validation of the proposed adsorption mechanism. Since the principal conclusion — that explicit short-range three-body quantum many-body interactions fundamentally suppress the adsorption of Cl^- and Br^- while leaving only a shallow adsorption minimum for I^- — has not been directly corroborated by experiment, the manuscript remains primarily a computational study. In my view, this substantially limits both the impact and the significance of the work, particularly for a journal such as Nature Communications, where strong experimental support or compelling validation of new theoretical predictions is generally expected.

We agree that the calculated HD-vSFG spectra are theoretical predictions and have deliberately described them as an independent spectroscopic consistency check, not as direct experimental measurements of the single-ion PMFs. We have made this distinction explicit throughout the revised manuscript.

A one-to-one experimental validation of the central quantity examined here cannot be performed because the intrinsic free-energy profile of a single isolated halide ion relative to the air/water interface is not experimentally accessible. Experiments necessarily probe electroneutral, finite-concentration electrolyte solutions and therefore include counterion contributions, ion–ion correlations, possible ion pairing, and concentration-dependent interfacial restructuring. Simulating those systems would address a related but different thermodynamic question. The single-ion limit was chosen deliberately to isolate the intrinsic propensity at the heart of the Wagner and Onsager–Samaras theories, without these additional effects. Requiring a finite-concentration electrolyte measurement to validate the isolated-ion PMF would therefore conflate two physically distinct problems.

This distinction is particularly important because the experimental literature has not converged on a unique microscopic interpretation. Two contemporaneous vSFG studies of aqueous sodium halide interfaces reported different spectral signatures and emphasized different manifestations of ion-specific interfacial perturbation [J. Phys. Chem. B 108, 2252–2260 (2004); J. Phys. Chem. B 108, 5051–5059 (2004)]. Subsequent studies involving the Bonn group reached sharply contrasting microscopic pictures, ranging from extreme surface enrichment, with inferred interfacial ion concentrations several times those in the bulk [Nat. Commun. 5, 4083 (2014)], to an ion-depleted outermost layer with ion enrichment confined to the subsurface [Nat. Chem. 16, 644–650 (2024)]. The latter study itself notes that experimental data alone are insufficient to disentangle the broad spectral components of aqueous electrolyte interfaces unambiguously, underscoring the need for molecular simulations. These divergent interpretations demonstrate the difficulty of inferring microscopic ion distributions indirectly from the vibrational response of interfacial water and motivate a clean, controlled determination of the intrinsic single-ion free-energy profile, which is precisely what the present study provides. Establishing this reference is a necessary foundation for subsequent extensions to finite-concentration electrolyte solutions.

The HD-vSFG calculations provide a strong connection to experimental measurements reported in the literature and, importantly, already establish clear constraints on the range of possible interpretations. They were performed for the same systems and the same four-level interaction hierarchy as the PMFs. The spectra reproduce the free-energy ordering: the polarizable baseline yields hydrogen-bonded O–H signatures for I^-, Br^-, and Cl^-; the pairwise model produces no corresponding positive feature; and the full many-body model yields an appreciable response only for I^-. Thus, the thermodynamic prediction has an independent spectroscopic consequence rather than being inferred from the PMFs alone.

The absence of a directly measurable isolated-ion PMF does not make the theoretical result qualitative or unvalidated. MB-pol and MB-nrg have already been extensively validated against cluster energetics and spectra, bulk hydration structure, EXAFS spectra, vibrational spectra, hydration free energies, vapor–liquid properties, and interfacial spectroscopy before being applied here. The present PMFs and HD-vSFG spectra are therefore genuine out-of-sample predictions of a broadly validated Hamiltonian. The absence of a paired measurement of an experimentally inaccessible single-ion quantity does not constitute a validation gap.

We agree that the HD-vSFG spectra are theoretical predictions rather than direct measurements of the single-ion adsorption free energy. We disagree, however, that this limits the significance of the work or leaves its central conclusion unsupported. The single-ion calculation addresses a fundamental quantity that experiments cannot isolate, while the HD-vSFG response provides an independent connection to experimentally observable interfacial water structure.

3. One of the central claims of this manuscript is that explicit short-range three-body quantum many-body interactions qualitatively suppress the adsorption of Cl^- and Br^- while leaving only a shallow adsorption minimum for I^-. Although the simulations clearly demonstrate this change in the free-energy profiles, the manuscript does not provide a convincing microscopic explanation for the underlying physical mechanism. The analyses presented largely describe what changes after the inclusion of the three-body interactions, but they do not explain why these interactions fundamentally alter the adsorption behavior of different halide

ions. In particular, the manuscript does not establish a clear causal relationship between the explicit three-body quantum effects (e.g., exchange repulsion, charge penetration, and charge transfer) and the resulting changes in water reorganization, hydrogen-bond structure, or interfacial thermodynamics. Consequently, the work remains largely descriptive and computational rather than providing new mechanistic insight into the origin of the revised adsorption behavior. In my opinion, this lack of physical understanding substantially limits the conceptual advance of the study and falls short of the level of mechanistic insight expected for publication in Nature Communications.

We respectfully disagree that the analysis is merely descriptive. The model hierarchy constitutes a controlled intervention on the interaction Hamiltonian: the water model, system geometry, sampling procedure, collective variable, and thermodynamic conditions are held fixed, while specific physical terms are selectively introduced. This provides a direct causal test of their effect on the adsorption thermodynamics, rather than a post hoc correlation.

In particular:

- comparing the pairwise (2B)-MB-nrg and (2B+NB)-MB-nrg models isolates the effect of classical many-body polarization
- comparing TTM-nrg and (2B+NB)-MB-nrg reveals the effect of replacing conventional empirical short-range repulsion and dispersion with a CCSD(T)-trained 2-body representation
- comparing (2B+NB)-MB-nrg and (2B+3B+NB)-MB-nrg isolates the consequences of explicit short-range 3-body ion–water–water interactions
- decomposing the full Helmholtz free energy quantifies how these changes propagate into ion–water desolvation, water cavity/reorganization, and entropy

The resulting mechanism is explicit. Pairwise interactions alone yield image-charge-like exclusion. Classical many-body polarization stabilizes the heavier halides too strongly. Accurate short-range 2-body physics substantially reduces this stabilization, while explicit 3-body interactions alter both the direct ion–water energetics and the solvent configurations sampled near the interface. These changes eliminate the Cl^- and Br^- minima and leave only a finely balanced iodide state in which a localized water cavity/reorganization minimum offsets ion–water desolvation but is opposed by entropy. The same hierarchy is independently reflected in the HD-vSFG response.

This analysis is quantitative rather than qualitative. The manuscript reports the complete PMFs, total configurational and entropic contributions, ion–water and water cavity/reorganization energies, explicit 2-body and 3-body contributions, permanent and induced electrostatics, and the resulting spectroscopic response. A single local descriptor, such as a hydrogen-bond count or orientational order parameter, would not uniquely capture this mechanism because the effect is intrinsically collective and many-body: modifying the 3-body ion–water term changes both the local interaction energy and the ensemble of interfacial solvent configurations through polarization and reorganization.

The mechanistic insight therefore does not rely on a post hoc phenomenological interpretation. It follows directly from controlled changes to the interaction Hamiltonian and from the corresponding quantitative thermodynamic response. This controlled resolution is enabled by the modular MB-nrg hierarchy and is not available in the same internally consistent manner from conventional force fields or from DFT-based simulations. We therefore respectfully maintain that the controlled many-body hierarchy provides a mechanistically resolved and quantitatively testable explanation of why lower-order descriptions fail and why only iodide retains weak surface activity.

Response to Reviewer 3

The authors have adequately addressed all my comments and the paper is ready for publication.

We thank the Reviewer for the careful evaluation of our manuscript and for supporting publication of the revised version in Nature Communications. We appreciate the Reviewer's constructive comments, which helped us improve the clarity, reproducibility, and presentation of the work.
