# Supplementary material for: Many-body interactions resolve halide distribution at the air/water interface
Source: Nat Commun. 2026 Aug 29;17:10313. doi: 10.1038/s41467-026-77205-9 (PMC13620159; doi:10.1038/s41467-026-77205-9)
Supplement: Supplementary file 4 [file 41467_2026_77205_MOESM4_ESM.zip › source_data/pdf/figureS10.pdf]

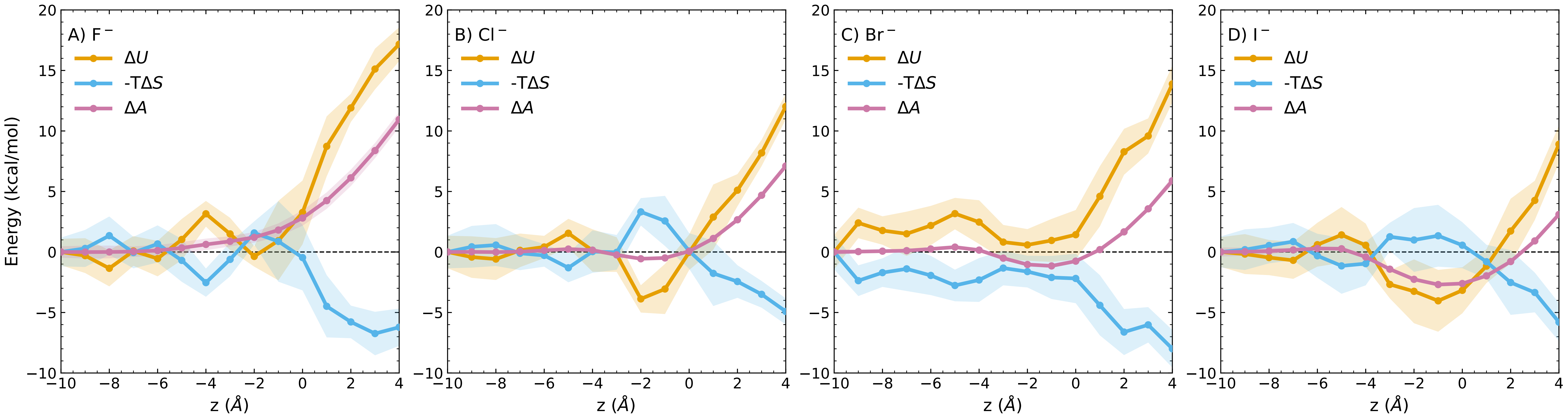

A) F$^-$
B) Cl$^-$
C) Br$^-$
D) I$^-$
ΔU
-TΔS
ΔA
Energy (kcal/mol)
z (Å)
20
15
10
5
0
−5
−10
−8
−6
−4
−2
2
4
