# Supplementary material for: Many-body interactions resolve halide distribution at the air/water interface
Source: Nat Commun. 2026 Aug 29;17:10313. doi: 10.1038/s41467-026-77205-9 (PMC13620159; doi:10.1038/s41467-026-77205-9)
Supplement: Supplementary file 4 [file 41467_2026_77205_MOESM4_ESM.zip › source_data/pdf/figure2.pdf]

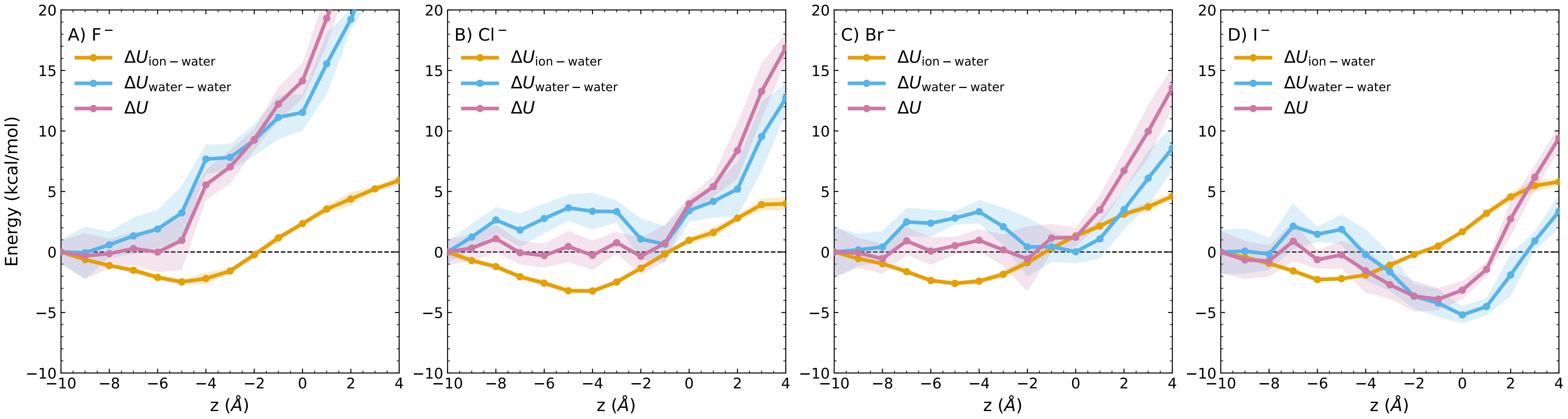

A) F⁻
B) Cl⁻
C) Br⁻
D) I⁻
$\Delta U_{\mathrm{ion-water}}$
$\Delta U_{\mathrm{water-water}}$
ΔU
Energy (kcal/mol)
z (Å)
