# Supplementary material for: Many-body interactions resolve halide distribution at the air/water interface
Source: Nat Commun. 2026 Aug 29;17:10313. doi: 10.1038/s41467-026-77205-9 (PMC13620159; doi:10.1038/s41467-026-77205-9)
Supplement: Supplementary file 4 [file 41467_2026_77205_MOESM4_ESM.zip › source_data/pdf/figureS4.pdf]

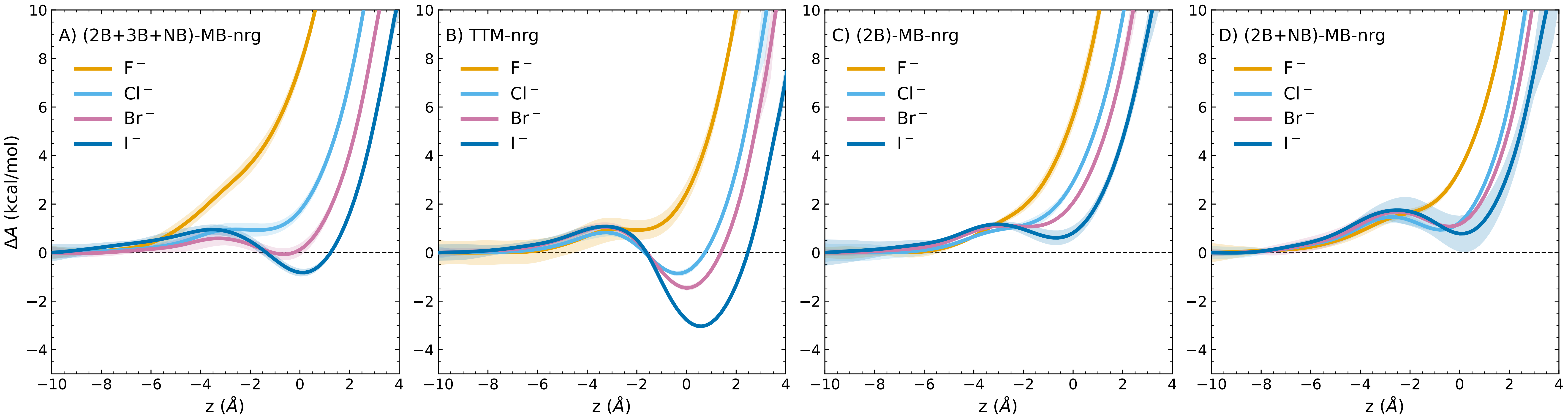

A) (2B+3B+NB)-MB-nrg
B) TTM-nrg
C) (2B)-MB-nrg
D) (2B+NB)-MB-nrg
F⁻
Cl⁻
Br⁻
I⁻
ΔA (kcal/mol)
z (Å)
