# Supplementary material for: Many-body interactions resolve halide distribution at the air/water interface
Source: Nat Commun. 2026 Aug 29;17:10313. doi: 10.1038/s41467-026-77205-9 (PMC13620159; doi:10.1038/s41467-026-77205-9)
Supplement: Supplementary file 4 [file 41467_2026_77205_MOESM4_ESM.zip › source_data/pdf/figureS7.pdf]

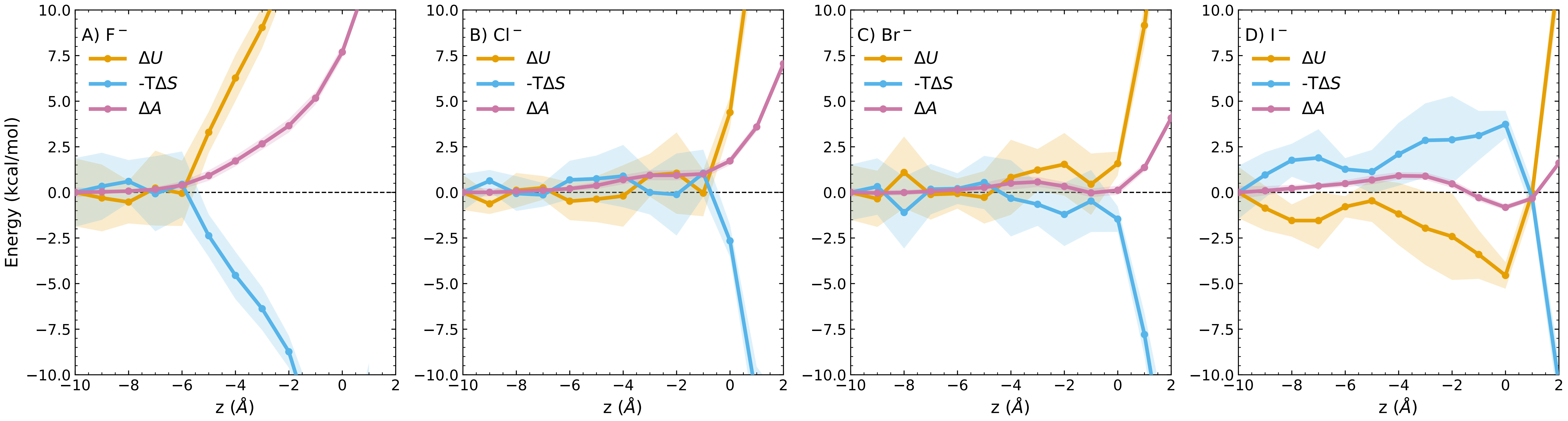

A) F⁻
B) Cl⁻
C) Br⁻
D) I⁻
ΔU
-TΔS
ΔA
Energy (kcal/mol)
z (Å)
10.0
7.5
5.0
2.5
0.0
−2.5
−5.0
−7.5
−10.0
−10
−8
−6
−4
−2
0
2
