# Supplementary material for: Many-body interactions resolve halide distribution at the air/water interface
Source: Nat Commun. 2026 Aug 29;17:10313. doi: 10.1038/s41467-026-77205-9 (PMC13620159; doi:10.1038/s41467-026-77205-9)
Supplement: Supplementary file 4 [file 41467_2026_77205_MOESM4_ESM.zip › source_data/pdf/figureS6.pdf]

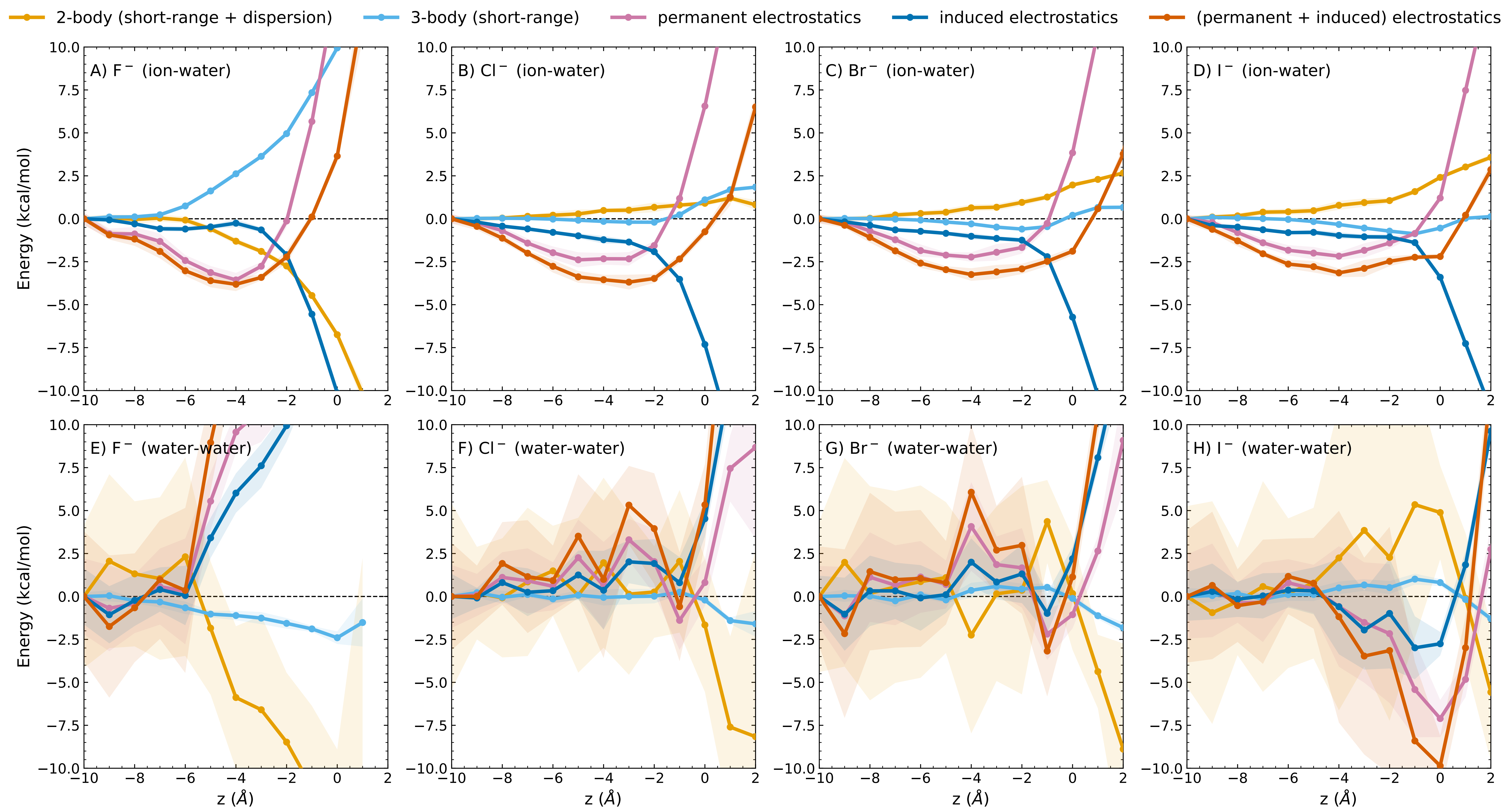

2-body (short-range + dispersion)
3-body (short-range)
permanent electrostatics
induced electrostatics
(permanent + induced) electrostatics
A) F⁻ (ion-water)
B) Cl⁻ (ion-water)
C) Br⁻ (ion-water)
D) I⁻ (ion-water)
E) F⁻ (water-water)
F) Cl⁻ (water-water)
G) Br⁻ (water-water)
H) I⁻ (water-water)
Energy (kcal/mol)
z (Å)
